# SMC-like Wadjet system prevents plasmid transfer into *Clostridium cellulovorans*

**DOI:** 10.1007/s00253-025-13551-w

**Published:** 2025-07-23

**Authors:** Aline I. Schöllkopf, Armin Ehrenreich, Wolfgang Liebl

**Affiliations:** Chair of Microbiology, Technical University of Munich, TUM School of Life Sciences, Emil-Ramann-Str. 4, Freising, 85354 Germany

**Keywords:** *Clostridium cellulovorans*, Wadjet system, Transconjugation, Plasmid defense system, CRISPR-Cas, Restriction-modification system

## Abstract

**Abstract:**

This study demonstrates the impact of a Structure Maintenance of Chromosome (SMC)-like Wadjet system on the horizontal gene transfer of plasmids by conjugation to a recipient that naturally containing such a system for the first time. A *Clostridium cellulovorans* mutant with dramatically improved efficiency to receive plasmid DNA by conjugation was isolated and sequenced. Three spontaneous chromosomal deletions included a type II restriction-modification system, a putative CRISPR system, and a cluster of ORFs named *jetABCD* encoding a putative Wadjet system. Since nearly nothing is known about the role of naturally occurring Wadjet systems in their native host bacteria, markerless chromosomal deletion of *jetABCD* in the *C. cellulovorans* wildtype strain 743B was achieved and the effect on conjugative plasmid uptake was studied. The transconjugation frequency of the *jetABCD* mutant was increased by about five orders of magnitude compared to wildtype *C. cellulovorans* recipient cells. Bioinformatic analysis of genome sequences of the *Bacillota* phylum revealed near-complete mutually exclusive possession of either plasmids < 40 kb or *jetABCD* genes, indicating high efficiency of Wadjet systems in small plasmid prevention in bacteria. Importantly, the implications of this study go beyond the case of *C. cellulovorans*. Our study demonstrates that the eradication of Wadjet systems can dramatically improve the uptake of recombinant plasmids and thereby enhance genetic engineering of bacterial strains of interest for biotechnological applications.

**Key points:**

• Native Wadjet system inhibits plasmid transfer by conjugation in *C. cellulovorans*

• Deleting *jetABCD* increased plasmid uptake by about five orders of magnitude

• Possession of Wadjet systems efficiently block plasmid maintenance in *Bacillota*

**Graphical Abstract:**

**Figure:**
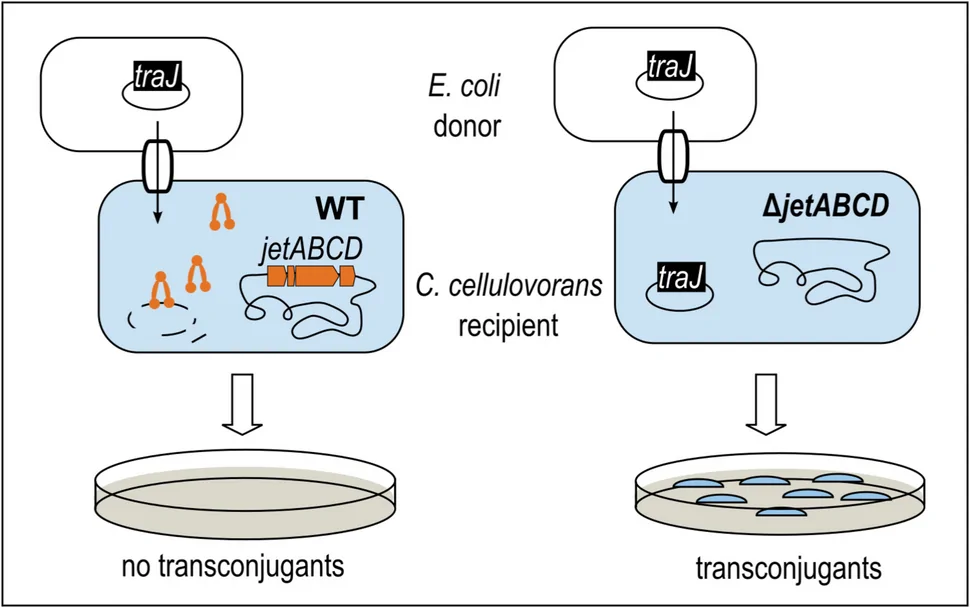

**Supplementary Information:**

The online version contains supplementary material available at 10.1007/s00253-025-13551-w.

## Introduction

In their natural habitats, bacteria are permanently exposed to various threats by intruding selfish genetic information from conjugative plasmids, DNA transfer by natural transformation, and from phage infections. Throughout evolution bacteria have evolved a broad spectrum of mechanisms to evade hostile genetic information. Some widely spread strategies of bacteria to counteract the intrusion of foreign nucleic acids like phage DNA and RNA, self-transmissible and mobilizable plasmids, and other mobile elements have been studied in detail. Traditionally, restriction-modification (RM) and abortive infection (Abi) systems, as well as CRISPR (Clustered Regularly Interspaced Short Palindromic Repeats)-Cas systems have been in the focus of research (reviewed by Hampton et al. 2020). In addition, systematic studies based on an above-random genomic co-localization of known defense systems with certain genes of unknown function lately suggested over 50 previously unknown putative defense systems often organized in so-called defense islands (Makarova et al. 2011; Doron et al. 2018; Gao et al. 2020; Millman et al. 2022; Jaskólska et al. 2022). Bacterial genomes often encode several different defense systems, with an average of 5.2 to 7.1 of putative defense systems per genome (Millman et al. 2022; Georjon et al. 2023). Although most defense systems appear to be antiphage systems, some, including type IV CRISPR-Cas, RM, SMC-family Wadjet, DISARM (defense island system associated with restriction-modification), Lamassu (DdmABC), or DdmDE act against plasmids (Makarova et al. 2011; Doron et al. 2018; Gao et al. 2020; Millman et al. 2022; Bravo 2024; Jaskólska et al. 2022).

Defense systems that have evolved in most bacterial lineages to counteract the intrusion of foreign DNA also are important obstacles for the genetic engineering of host bacteria for biotechnology. For example, the genetic accessibility of *Clostridium cellulovorans* (Sleat et al. 1984), a cellulolytic, strictly anaerobic bacterium with great potential for the sustainable production of bulk chemicals from (hemi-)cellulosic waste material, was reported to be strongly limited due to RM systems (Yang et al. 2016). This is in good agreement with the observation that RM systems are the most common defense mechanism in bacteria found in approximately 70–80% of the bacterial genomes (Oliveira et al. 2014; Millman et al. 2022; Tesson et al. 2022). RM systems have also been described to interfere with the transfer of extrachromosomal DNA in other clostridia (Minton et al. 2016). Recently, we improved the genetic accessibility of *C. cellulovorans* by targeted chromosomal deletion of either the type I, the type II, or the type III restriction endonucleases of the respective RM systems. These studies reported the first markerless chromosomal deletion in *C. cellulovorans*, which were conducted using Cas9 or *codBA* counterselection strategies (Schöllkopf et al. 2025; Almeida et al. 2025). Although the introduction of recombinant DNA into *C. cellulovorans* was significantly enhanced by these measures, the efficiency of introduction of recombinant DNA remained relatively low, possibly because of other defense systems still in place after inactivation of the RM systems and the general underdevelopment of genetic tools for this low guanine-cytosine (GC) anaerobe.

While RM systems act as innate immune systems that detect foreign DNA based on the absence of specific methylation patterns determined by the cognate methyltransferases, CRISPR-Cas systems work in analogy to an adaptive immune response in bacteria and prevent re-infection by previously encountered invasive elements (Hille et al. 2018). Such systems have been detected in about 40% of the fully sequenced bacterial genomes (Tesson et al. 2022; Zaayman and Wheatley 2022; Millman et al. 2022). In general, CRISPR-Cas systems consist of various *cas* genes and a CRISPR array of short, direct repeats separated by variable DNA spacer sequences (Koonin et al. 2017). Homologies between spacers and plasmids indicate that endogenous CRISPR-Cas systems are capable of interfering not only with phage infection, but also with plasmid transfer via transconjugation (Bolotin et al. 2005; Mojica et al. 2005). Thus, like RM systems they represent a potential barrier to genetic manipulation.

Beside these well-established systems interfering with foreign nucleic acid uptake, a new class of relatively rare (found in < 10% of bacterial genomes) defense systems was discovered, which is in vitro active against extrachromosomal circular DNA molecules. The Wadjet system (named after the ancient Egyptian tutelary goddess Wadjet) utilizes a structural maintenance of chromosomes (SMC)-like complex (Doron et al. 2018). SMC and related bacterial Smc-ScpAB, MukBEF, or MksBEF systems are involved in critical processes in chromosome organization, genome segregation, transcriptional control, but also in cellular defense (Panas et al. 2014; Jaskólska et al. 2022; Liu et al. 2022; Deep et al. 2022; Jeppsson 2025). In general, SMC-like systems are arranged in three-gene operons consisting of the genes for a SMC ATPase subunit, a kleisin-family protein, and a kleisin interacting tandem winged-helix element of SMC complexes (KITE) protein. Wadjet operons in addition contain a fourth ORF encoding JetD, a toprim domain-containing nuclease responsible for DNA-cleavage (Deep et al. 2022; Liu et al. 2023). Current models embrace that the shape and size of the DNA is sensed by the SMC complex (JetABC), which entraps circular DNA and performs ATP-dependent loop extrusion. Plasmid recognition is initiated by the encounter of the two SMC motor units of a dimeric JetABCD complex, which does not occur on linear DNA fragments and rarely on large circular DNA molecules such as chromosomes. The SMC-like complexes after complete extrusion of the circular plasmid are blocked in their movement and enclose a U-shaped DNA segment, which is kinked and ultimately cleaved by the binding of JetD (Roisné-Hamelin et al. 2024). The estimated plasmid cut-off size for recognition as a target for cleavage by JetD is about > 50–100 kb (Liu et al. 2023). Despite a large gain of knowledge in the last few years about how Wadjet systems may recognize and destroy small circular plasmids at the molecular level (Deep et al. 2022; Liu et al. 2023; Weiß et al. 2023; Roisné-Hamelin et al. 2024), information is very scarce about their function in vivo in bacteria naturally equipped with such systems. In *Mycobacterium smegmatis*, a null mutation in EptABCD, the JetABCD-homologous system of this organism, displayed a phenotype of increased plasmid electroporation efficiency, and EptC was found to impair maintenance of foreign plasmids (Panas et al. 2014). The type I Wadjet system of *Corynebacterium glutamicum* (called MksBEFG) was found to have an effect on plasmid copy number and to be localized to the cell poles in this bacterium (Böhm et al. 2020; Weiß et al. 2023). The effect of Wadjet systems on DNA uptake via conjugation, a mechanism of horizontal gene transfer (HGT) highly relevant, e.g., for the spreading of transmissible antibiotic resistance determinants, in bacteria naturally equipped with such systems has not been studied before.

In this work, we analyzed a spontaneous mutant of *C. cellulovorans* with a dramatically improved transconjugation efficiency when used as the recipient strain in mating experiments with *E. coli* as donor. Transconjugation or conjugation is defined as a horizontal gene transfer process in which the donor bacterium transfers plasmid DNA to another species as a recipient cell via direct cell–cell contact, resulting in a plasmid-bearing recipient cell named transconjugant. In this study, we define transconjugation frequency as the ratio of plasmid-bearing recipient cells in comparison to plasmid-free recipient cells after transconjugation. Genomic sequencing of the spontaneous mutant revealed the deletion of the type II RM system we have characterized previously (Schöllkopf et al. 2025), the partial deletion of a putative CRISPR-Cas locus, and the deletion of putative Wadjet genes. In order to single out among the observed deletions, which effect specifically the Wadjet deletion had on the efficiency of *C. cellulovorans* to act as the recipient during transconjugation, we decided to delete this system from the wildtype *C. cellulovorans* chromosome, using a Cas9 nickase system for markerless chromosomal deletion. The results presented in this study aim to show that the putative Wadjet system in the *C. cellulovorans* 743B wildtype strain functions as an efficient anti-plasmid system and that it thus represents a significant barrier for the genetic accessibility in *C. cellulovorans.* An additional goal of the deletion of the Wadjet system was to obtain a rationally improved *C. cellulovorans* strain for facilitated metabolic engineering in the future.

## Material and methods

### Growth conditions

*C. cellulovorans* 743B was purchased from the Deutsche Sammlung von Mikroorganismen und Zellkulturen GmbH (DSMZ, Braunschweig, Germany) and grown anaerobically in DMSZ 520 medium with 5 g L^−1^ cellobiose or glucose at 37 °C. Yeast extract (2 g L^−1^, 2YE) was substituted by tryptone (2 g L^−1^, 2T) in selection and induction medium, and the medium was solidified with 1% (w/v) agar (all Carl Roth GmbH, Karlsruhe, Germany). 10 mM or 100 mM HEPES (10H or 100H, respectively) were supplemented as indicated. For long-time storage, the culture was supplemented with 25% glycerol (v/v) and stored at − 80 °C. *E. coli* strains (New England Biolabs (NEB), Ipswich, MA, USA) were grown aerobically at 37 °C with agitation at 180 rpm in liquid lysogenic broth (LB) medium or on solid LB agar (1.5% (w/v)). The cell growth was controlled photometrically via optical density (OD_600_) or in McFarland units (McF) by use of a McFarland densitometer (DEN-1B Densitometer; Grant Instruments, Royston, United Kingdom). Appropriate antibiotics and supplements were added at the following concentrations: tetracycline, 10 µg mL^−1^; chloramphenicol, 34 µg mL^−1^; thiamphenicol, 10 µg mL^−1^; theophylline, 5 mM. All strains used in this study are listed in Table 1.

**Table 1:** List of strains used in this study

Stain	Characteristic	Source
*Clostridium cellulovorans* 743B	Wildtype	DSMZ
*Clostridium cellulovorans* Δ*engE*/2	743B with deletion of Clocel_3559 and spontaneous deletion of Clocel_3950-4071, Clocel_2327-2351, and Clocel_0831-0854	This study
*Clostridium cellulovorans* Δ*jetABCD*	743B with deletion of Clocel_0831-Clocel_0834	This study
*Escherichia coli* NEB10B	*Δ(ara-leu) 7697 araD139 fhuA ΔlacX74 galK16 galE15 e14- ϕ80dlacZΔM15 recA1 relA1 endA1 nupG rpsL (Str*^*R*^*) rph spoT1 Δ(mrr-hsdRMS-mcrBC)*	NEB
*E. coli* CA434	R702 plasmid	(Purdy et al. 2002)

### DNA techniques and genetic manipulation

Routine molecular biological procedures were performed according to the manufacturer’s protocols. PCR fragments were amplified using Q5 High-Fidelity DNA Polymerase (New England Biolabs, Ipswich, MA, USA) for molecular cloning or Phire II Hot Start DNA Polymerase (Thermo Fisher Scientific, Waltham, MA, USA) for analytical reactions. Enzymes were purchased from New England Biolabs (Ipswich, MA, USA) or Thermo Fisher Scientific (Waltham, MA, USA), and oligonucleotides were purchased from Sigma*-*Aldrich (Taufkirchen, Germany) and listed in Table 2. The NucleoSpin® PCR clean-up gel extraction and Plasmid EasyPure kits (Macherey–Nagel, Düren, Germany) were used for DNA purification.

**Table 2:** Oligonucleotides used in this study. Restriction enzyme recognition sites are underlined

Name	Sequence (5′—> 3′)	Application
AIS1_rv	AGGTGACTGATCTAGAGCCGGTCATAGCTGTTTCCTG	pMTL83151
AIS2_fw	CTCGAGATAAGGCCTAATGGCGCGCCGCCATTATTTTTTTGAACAATTGACAATTCATTTC	pMTL83151, XhoI, AscI
AIS3_fw	GCTATGACCGGCTCTAGATCAGTCACCTCCTAGCTGAC	*cas9*
AIS4_rv	GGCACAAATAGCGTCGGATGG	*cas9*
AIS5_fw	ATCCGACGCTATTTGTGCCGATAGCTAAG	Artificial construct
AIS6_rv	AATGGCGGCGCGCCATTAGGCCTTATCTCGAGATTATACCTAGGACTGAGCTAGCTGTCAAACTGG	Artificial construct, AscI, XhoI
AIS7_fw	AATCTCGAGGAAACTTGACTGGTACAGGTGTTTTAGAGCTAGAAATAGCAAGTTAAAATAAGGCTAGTCCGTTATCAACTTGAAAAAGTGGCACCGAGTCGGTGCTTTTTTT	sgRNA, XhoI
AIS8_rv	AATAAAAGACGTCCATAAAAATAAGAAGCCTGCAAATGCAGGCTTCTTATTTTTATAAAAAAAGCACCGACTCGGTGCCACTTTTTCAAGTTG	sgRNA, AatII
AIS10_fw	TTCTTATTTTTATGGACGTCTTTTATTTCCAAAAGAACTTATTACTGTTTCTAAATCAATCC	gDNA, HR *engE*
AIS11_rv	TACAAAAAGGAGGATTTATCACTGAGATACTTATCAATTACATAATAAAACATGAAGG	gDNA, HR *engE*
AIS12_fw	TAATTGATAAGTATCTCAGTGATAAATCCTCCTTTTTGTATATTTCC	gDNA, HR *engE*
AIS13_rv	AAAAAATAATGGCGGCGCGCCGCAAATGGTGTTTATGGTATGAGTGTTATTACTGC	gDNA, HR *engE*, AscI
AIS14_fw	ATGGACGTCTGATTTTTGAAACTAATTGGGTGG	gDNA, HR jetABCD, AatII
AIS15_rv	TAAGATATCAAATTTAAATCACCACTTTCTGTTTC	gDNA, HR *jetABCD*
AIS16_fw	CTCATTTTGAAATTTTATTAAGATATCAAATTTAAATC	gDNA, HR *jetABCD*
AIS17_rv	TGGCGGCGCGCCTAGTGCTCTTTCCACATACAAATTTGC	gDNA, HR jetABCD, AscI
AIS18_fw	ATAATCTCGAGGCATTTAAGTTTCCTAATATTGTTTTAGAGCTAGAAATAGCAAGTTAAAATAAGG	sgRNA exchange, jetD target, XhoI
AIS19_rv	CTCGAGATTATACCTAGGACTGAGCTAGC	sgRNA exchange, XhoI
D1_ fw	TTACCAAGACAGATATGAAACTAGG	Check for deletion of *jetABCD*
D2_rv	TTTATCTTCACACATCTTTGACC	Check for deletion of *jetABCD*
D3_fw	ATGGATAAGAAATACTCAATAGGCTTAGATATCG	Check for plasmid, *cas9* gene
D4_rv	CAGTCACCTCCTAGCTGACTC	Check for plasmid, *cas9* gene
D5_fw	ACCTATTTCCACCAAATAATCAGC	Check for gDNA integrity, *cas* locus
D6_rv	AATTACAACTGCAATACAAGTGG	Check for gDNA integrity, *cas* locus
D7_fw	CACGAGCTCGAAATGAGGATAGTTTTATGAGTAAGAAATTTAC	Check for gDNA integrity, Clocel_4007-4008 locus
D8_rv	CACCTCGAGGATACACGGACTACACCTCC	Check for gDNA integrity, Clocel_4007-4008 locus

### Vector construction

The pNick deletion vector series was constructed using oligonucleotide primers AIS1-AIS13 and NEBuilder® HiFi DNA Assembly (New England Biolabs, Ipswich, MA, USA). Briefly, the *cas9* gene of *Streptococcus pyogenes* was amplified with simultaneous introduction of a D10A mutation for the inactivation of the RuvC domain. The theophylline-responding riboswitch E* (Topp et al. 2010; Cañadas et al. 2019) was modified with a NdeI site upstream of the start codon, and its transcription was designed to be driven by the *fdx* promoter region of *Clostridium saccharobutylicum* (Schöllkopf et al. 2025) omitting the native ribosome-binding site, and the J23119 promoter were derived from an artificial construct (Synthetic DNA fragment S1). The sgRNA and the *fdx* terminator of *Clostridium acetobutylicum* were generated by self-priming using AIS7 and AIS8. The upper and lower homologous recombination (HR) regions were amplified from genomic DNA. All fragments were ligated into a pMTL83151 backbone (Heap et al. 2009).

The deletion vector pNick-delJetABCD-sg1 was constructed by exchanging the HR regions at the AatII and an AscI restriction recognition site (AIS14-AIS17) using restriction enzyme-based cloning procedures, while the sgRNA seed region was modified in a second step by PCR amplification using primers bearing the new seed (AIS18 and AIS19), and subsequent XhoI digest-mediated ligation of the PCR product. All vectors used in this study are listed in Table 3.

**Table 3:** Plasmids used in this study

Plasmid	Relevant features	Reference
placORMI	P15A, tetR, P(lac), Clocel_4007 & Clocel_4008	(Schöllkopf et al. 2025)
pMTL83151	pCB102 replicon, ColE1, oriT, catP, MCS	(Heap et al. 2009)
pNick-delEngE	P_Csa-fdx-RS_, cas9 (D10A), P_J23119_, sgRNA, seed 1, 1 kb HR arms, pCB102 replicon, catP, ColE1, oriT	This study
pNick-delJetABCD	P_Csa-fdx-RS_, cas9 (D10A), P_J23119_, sgRNA, seed 1, 1 kb HR arms, pCB102 replicon, catP, ColE1, oriT	This study

### Triparental conjugation of *C. cellulovorans*

DNA transfer was performed by triparental conjugation with *E. coli* CA434 (Purdy et al. 2002) as helper strain and the *E. coli* NEB10B donor bearing the pMTL83151-based mobilizable plasmid of interest and the methylation plasmid placORMI described elsewhere (Schöllkopf et al. 2025). Briefly, *E. coli* donor and helper strains were grown until mid-exponential phase (McF = 3.5). Cells from 1 mL culture were washed two times in phosphate-buffered saline (PBS) (pH 7.4) and re-suspended in 0.25 mL early-exponential *C. cellulovorans* culture (McF = 2.5). The cell mixture (40 µL) was spotted onto a 2YE-100H mating plate containing 5 g L^−1^ glucose as substrate and 100 mM HEPES (pH 7.0). After 20 h incubation, the cell mass was re-suspended in 500 µL PBS, spread onto 2T-10H selection plates (typtone, cellobiose, 10 mM HEPES, pH 7.0) supplemented with 10 µg mL^−1^ thiamphenicol and incubated anaerobically for 2–3 days at 37 °C.

### Generation of chromosomal mutants

*C. cellulovorans* transconjugants were cultivated overnight in 2T cellobiose medium supplemented with 10 µg mL^−1^ thiamphenicol (2T_Thia_). A serial dilution was spread on induction plates containing 5 mM theophylline and 10 µg mL^−1^ thiamphenicol. When needed, the obtained colonies were re-streaked onto a theophylline-containing plate. The genotype was analyzed by amplifying the target region from colony cell mass.

Plasmid-cured deletion strains were obtained by cultivating clones with a chromosomal deletion in liquid medium without antibiotic pressure. To achieve this, plasmid-carrying deletion strains were inoculated into liquid 2T_Thia_ medium and subsequently transferred twice a day in antibiotic-free 2T medium. Dilution streaks of the cultures were done on 2T plates without antibiotics, respectively. Single colonies were replica-streaked on 2T plates with and without 10 µg mL^−1^ thiamphenicol. The plasmid loss of antibiotic-sensitive colonies was verified using a PCR assay targeting the *cas9* gene. Additionally, the colony was inoculated into liquid 2T medium with and without thiamphenicol to check for antibiotic sensitivity.

### Conjugation efficiency determination

Mating experiments were conducted using C. *cellulovorans* Δ*jetABCD* as recipient, *E. coli* CA434 as helper strain and *E. coli* NEB10B-pMTL85131 or *E. coli* NEB10B-pMTL85131/placORMI as donor. After the mating, the cell mass was collected and resuspended in 500 µL PBS. Subsequently, a ten-fold dilution series was prepared in PBS and spread onto 2T plates in the absence and the presence 10 µg mL^−1^ thiamphenicol by plating or applying 10 µL onto a plate in duplicates followed by application of the track-dilution technique. After 2 days of incubation at 37 °C, the colonies were counted and the conjugation efficiency was calculated as transconjugants per recipient cells.

### DNA sequencing and bioinformatic analysis

Genomic DNA was isolated from *C. cellulovorans* overnight cultures and sequenced with Oxford Nanopore Technology (ONT) Sequencing—Bacterial Genome Sequencing Analysis Pipeline v1.0 (Eurofins Genomics, Ebersberg, Germany) or Illumina NovaSeq 2 × 150 technology (Azenta/GENEWIZ Germany GmbH, Leipzig, Germany).

Sequence similarity analysis of ORFs and amino acid sequences of proteins were performing using BLAST (NCBI tool: https://blast.ncbi.nlm.nih.gov/Blast.cgi). Multiple sequences were aligned with Jalview’s Muscle algorithm using default settings (Waterhouse et al. 2009). Analysis and data bank queries were conducted in MATLAB 2024a (The MathWorks, Inc., Natick, MA, USA).

## Results

### Isolation of a *C. cellulovorans* mutant with increased conjugation efficiency

**Fig. 1:**
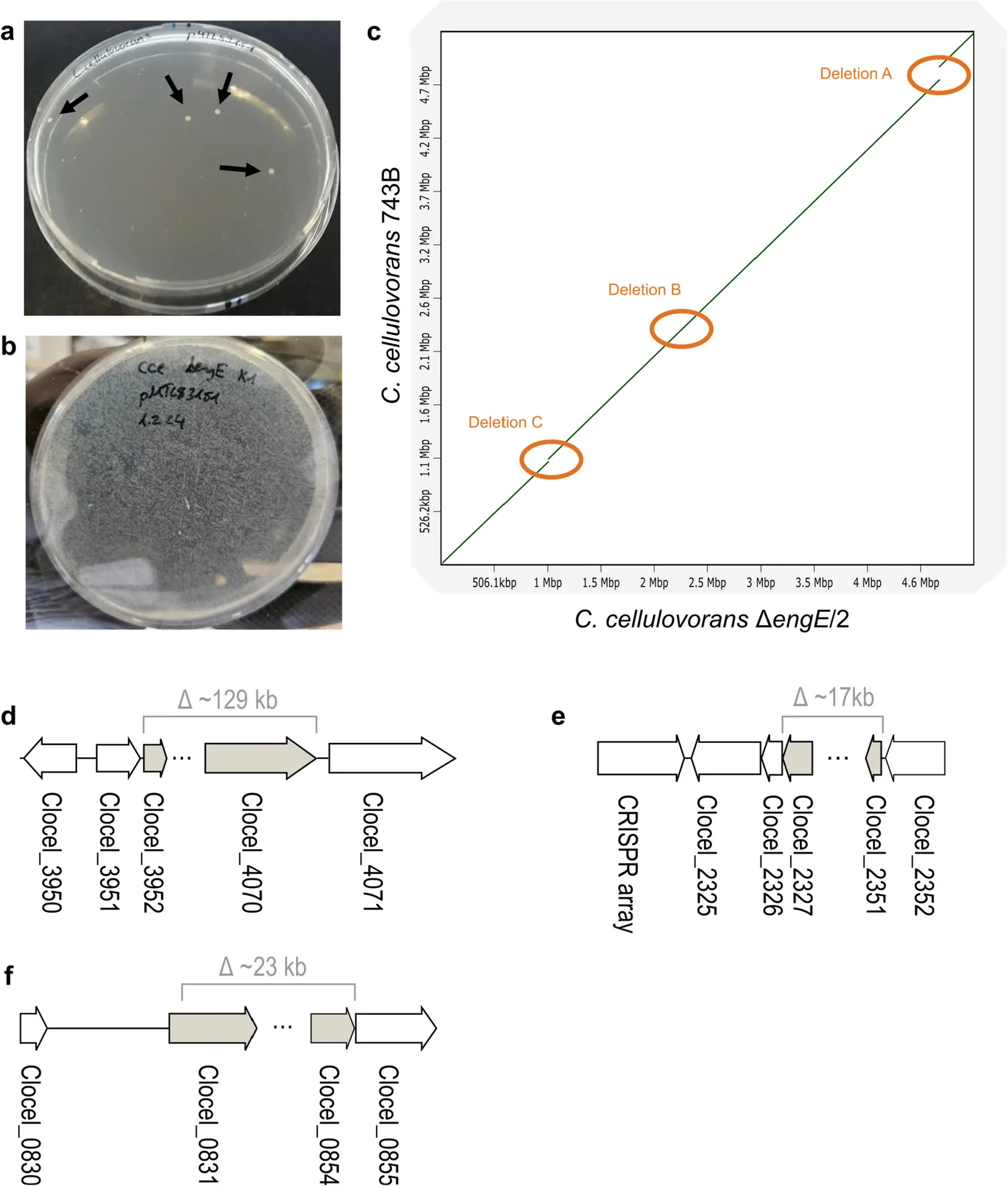
Improvement of transconjugation efficiency in a spontaneous mutant of *C. cellulovorans*. **a** Selection plate with merely few *C. cellulovorans* transconjugant colonies (marked with arrows) after a representative mating experiment with *E. coli* NEB10B-pMTL85131/placORMI as donor and the *C. cellulovorans* 743B wildtype strain as recipient. **b** Selection plate after mating with *E. coli* NEB10B-pMTL85131/placORMI as donor and the *C. cellulovorans* mutant strain Δ*engE*/2 as recipient. **c** Comparison of the genome sequence of *C. cellulovorans* 743B WT and the mutant strain Δ*engE*/2 by dot plot analysis (Cabanettes and Klopp 2018). The three deletion regions A, B and C that were found to be missing in the chromosome of the Δ*engE*/2 mutant strain are marked with orange ellipses. **d** Chromosomal location of deletion A. **e** Chromosomal location of deletion B. **f** Chromosomal location of deletion C

In previous work on the genetic engineering of the strictly anaerobic cellulose-degrading bacterium *C. cellulovorans*, we reported an efficiency of transconjugation from *E. coli* donor cells of about 10^−8^–10^−7^ transconjugants per recipient cell for the *C. cellulovorans* 743B wildtype strain (Schöllkopf et al. 2025). Applying in vivo methylation in *E. coli* with a *C. cellulovorans* methylase to protect the transconjugated DNA from restriction by the type II restriction endonuclease present in *C. cellulovorans* enables the transfer of recombinant plasmids, but the conjugation efficiency remained poor (Fig. 1a). Interestingly, the genetic accessibility was greatly improved in a strain that arose spontaneously during genetic engineering experiments, where we aimed to delete the *engE* gene to study the role of EngE, a cellulosomal endoglucanase, for cell surface attachment of the cellulosome. The strain was designated as *C. cellulovorans* Δ*engE*/2. The conjugation efficiency with this strain as recipient was approximately 10^−3^ transconjugants per recipient cell (Fig. 1b) compared to about 10^−7^ for the wildtype. Thus, we sequenced the chromosomal DNA of the mutant using Nanopore technology to determine potential genetic changes that could be responsible for the strain’s improved ability for plasmid uptake by conjugation. Three major chromosomal deletions were identified in strain Δ*engE*/2 (Fig. 1c–f): Deletion A (~ 129 kb), which ranged from Clocel_3950 to Clocel_4071, included the type II RM system operon that we have characterized before by generating a type II restriction deficient strain. The targeted deletion of this type II RM system resulted in a transconjugation efficiency of approximately 10^−6^ transconjugants per recipient cell (Schöllkopf et al. 2025). In a second deletion region (deletion B, ~ 17 kb), ranging from Clocel_2327 to Clocel_2351, a *devR*/*cas7* ortholog (Clocel_2327) and a *cas8* ortholog (Clocel_2328) of a putative endogenous CRISPR-Cas system were no longer present. The third deletion (deletion C, ~ 23 kb) involved the genes Clocel_0831 to Clocel_0854, including genes belonging to a putative Wadjet operon.

### Deletion of the putative *devR* and *cas8* genes readily occurs spontaneously

The putative CRISPR locus of *C. cellulovorans* contains a CRISPR array of 35 spacers, Clocel_2325 (49.27% similarity to a CRISPR-associated helicase Cas3 of *Anoxybacillus tepidamans*), Clocel_2326 (55.26% similarity to a hypothetical protein in *Clostridium butyricum*, and 39.56% to a type I-A Cas5a in *Bacillus thuringiensis*), Clocel_2327 (64.09% similarity to DevR of *C. butyricum*), and Clocel_2328 (34.18% similarity to a hypothetical protein of *C. butyricum*). Genome annotation of this region of the *C. cellulovorans* chromosome indicated *cas3*, *cas5*, *devR*/*cas7* and *cas8b*/*cas8a2* genes, representing part of a putative type I-B CRISPR system (Schwengers et al. 2021; Alkhnbashi et al. 2021).

**Fig. 2:**
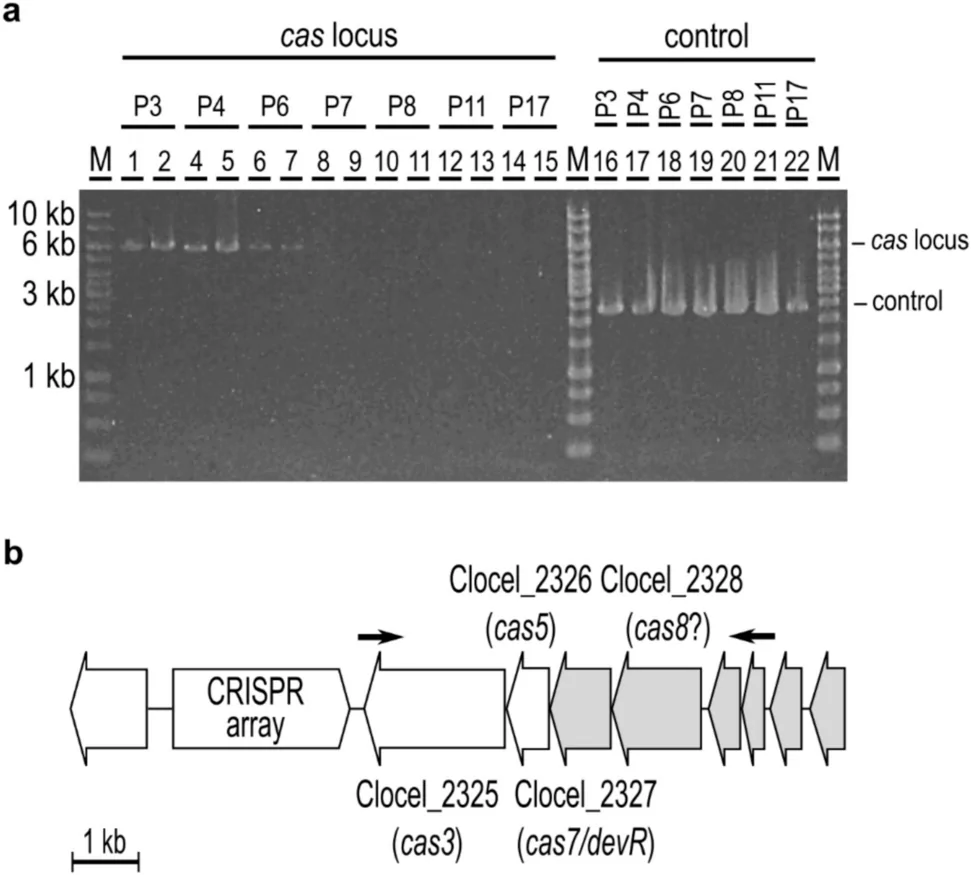
Spontaneous deletion of the *cas* locus. **a** The gDNA of wildtype *C. cellulovorans* 743B was isolated after 3 to 17 consecutive sub-cultivations after the strain was obtained from DSMZ (P3, P4, P6, P7, P8, P11, and P17). The *cas* locus was amplified using primers D5 and D6, resulting in a 6 kb product, while no product was expected when the locus was spontaneously deleted (lanes 1–15, the PCR was performed in technical duplicates). The integrity of the gDNA was controlled using primers D7 and D8 to amplify the Clocel_4007-4008 locus (lanes 16–22). **b** *C. cellulovorans* 743B chromosomal *cas* locus. The identity of the ORF *cas8* is hypothetical and requires further confirmation. The binding sites of the check primers Del5 and Del6 are indicated with arrows. The confirmed deletion of *C. cellulovorans* Δ*engE*/2 (see Fig. 1e) is shown in grey

Analysis of the *cas* ORFs, coding for the CRISPR locus indicated the spontaneous deletion of the putative *cas* ORFs in various independent cultures of *C. cellulovorans*. To confirm the nucleotide sequence of the chromosomal region carrying the *cas* ORFs in the wildtype strain, a glycerol stock from an early culture frozen away after the strain had been obtained from the DSMZ culture collection in 2020 was reactivated and inoculated into DMSZ 520 medium with 5 g L^−1^ cellobiose at 37 °C and the integrity of the chromosomal *cas* region was monitored over time. The *cas* region’s presence was confirmed in early cultures by PCR, but the region was spontaneously lost after around six consecutive sub-cultivations (Fig. 2).

To determine if these spontaneous *cas* deletion mutations appearing after repeated sub-cultivation were the same as observed in the three-fold deletion strain Δ*engE*/2, genome resequencing of four cultures, which were inoculated from different glycerol stocks, was carried out and identified deletions in the Clocel_2327 gene downstream of the sequence 5′-…TTGCC ACTAT CACTT A-3′ and upstream of 5′-ACTTA TATGT AAAGT T…−3′ in Clocel_2352. Apparently, a fusion of the “ACTTA” sequence motif which is present in both the Clocel_2327 and the Clocel_2352 genes had occurred.

The CRISPR spacer sequences were investigated by BLASTn similarity search against the core_nt database to identify potential targets in plasmids or invasive DNA elements like phages. However, no potential target of the CRISPR spacers was identified; thus, their origin and the functionality of the *C. cellulovorans* 743B CRISPR-Cas system remain obscure.

### Similarity of the Jet proteins of *C. cellulovorans* with the Jet proteins of *Bacillus cereus* and other putative Jet protein orthologs

**Fig. 3:**
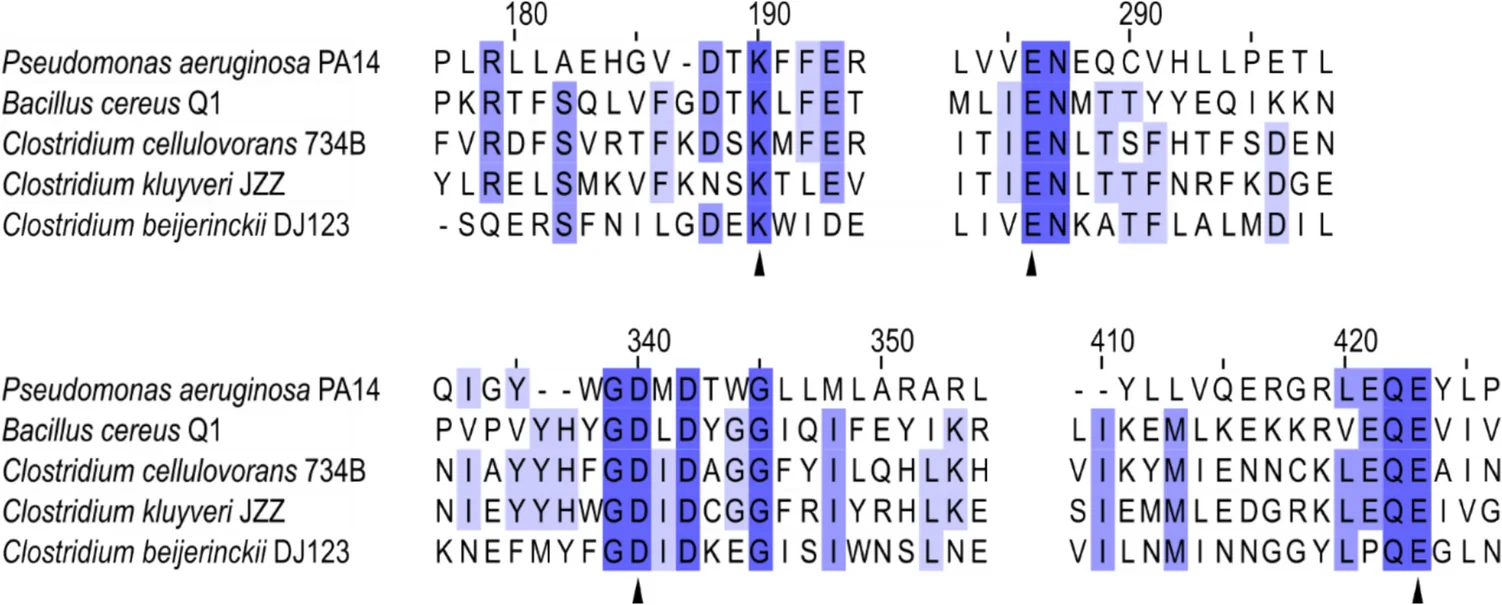
Alignment of JetD amino acid sequence segments. The protein sequences of JetD proteins from *Bacillus cereus* Q1, *Pseudomonas aeruginosa* PA14, *C. cellulovorans* 734B, *Clostridium kluyveri* JZZ, and *Clostridium beijerincki* DJ123 were aligned using Jalview’s Muscle algorithm with default settings. Conserved amino acids are shown in blue, and the experimentally confirmed essential residues of the JetD enzymes from *Bacillus cereus* Q1 and *Pseudomonas aeruginosa* PA14 are indicated with arrows

Previous work led to discovery of the Wadjet system of *Bacillus cereus* Q1 by introduction of the corresponding genes comprising this system into a heterologous host, *B. subtilis* (Doron et al. 2018). Comparative sequence analysis of the putative *C. cellulovorans* Wadjet proteins encoded by ORFs Clocel_0831 to Clocel_0834 revealed about 25.16%, 26.53%, 35.68%, and 26.62% similarity with *Bacillus cereus* Q1 JetA, JetB, JetC, and JetD, respectively. Next, the putative nuclease JetD, which is the signature protein of the Wadjet operon, was analyzed in detail. Previous reports revealed four conserved and catalytic active amino acid residues in JetD from *Bacillus cereus* Q1 and *Pseudomonas aeruginosa* PA14 (Deep et al. 2022). We carried out an amino sequence alignment of the putative JetD proteins of *C. cellulovorans* 743B, *Clostridium beijerincki* DJ123 and *Clostridium kluyveri* JZZ to identify conserved residues. *C. beijerincki* DJ123 served as an example for clostridia without a reported plasmid, while *C. kluyveri* JZZ carries a 58.6 kb plasmid. The alignment shown in Fig. 3 confirms the conservation of the essential amino acid residues in all JetD primary structures. This observation reinforces the assumption of a functional Wadjet system in *C. cellulovorans*. An AlphaFold prediction of the three-dimensional fold and positions of the active site residues of *C. cellulovorans* JetD, as well as a structural comparison with other predicted or experimentally confirmed JetD structures, is shown in supplemental Figure S2.

### Deletion of the chromosomal *jetABCD* genes in *C. cellulovorans* 743B and its effect on transconjugation efficiency

The functionality of the putative Wadjet system of *C. cellulovorans* was experimentally evaluated by markerless deletion of the *jetABCD* genes from the *C. cellulovorans* chromosome. Flanking regions, covering 1 kb each up- and downstream of the putative Wadjet system-encoding genes (*jetABCD*, ORFs Clocel_0831, Clocel_0832, Clocel_0833, and Clocel_0834), were designed to retain the start of Clocel_0831 and stop codon of Clocel_0834. The fused flanks were inserted into the vector pNick, and the seed sequence of the guide RNA was designed to target the Clocel_0834 (*jetD*) locus.

**Fig. 4:**
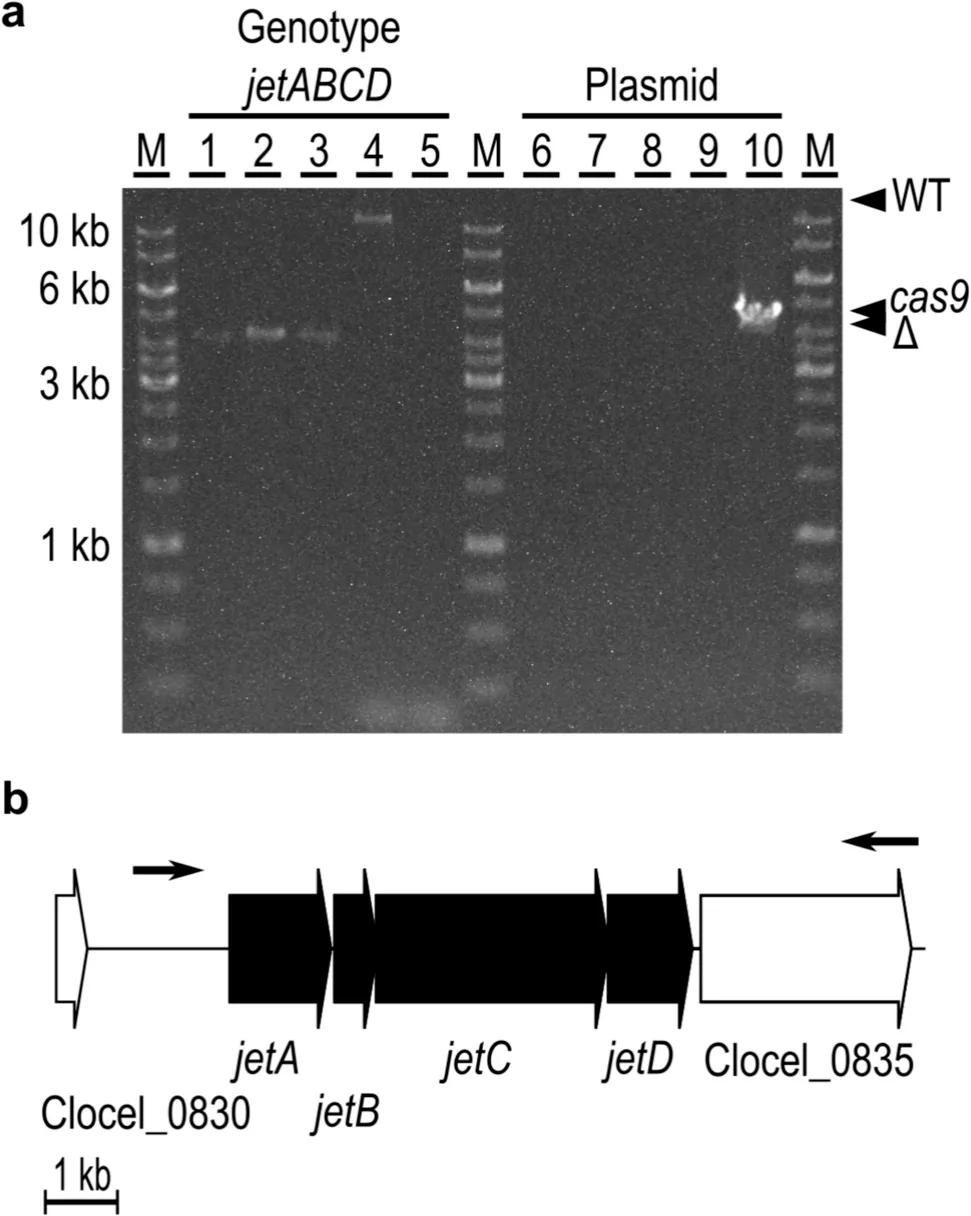
Deletion of the putative Wadjet gene cluster *jetABCD* in the *C. cellulovorans* 743B wildtype strain. **a** PCR analysis with primers D1 and D2 either confirmed the chromosomal deletion of *jetABCD* (4.3 kb) or showed the wildtype genotype (10.7 kb) (lanes 1–5). Confirmation of plasmid curing was done by PCR with primers D3 and D4 targeting *cas9* (4.1 kb) (lanes 6–10). Lanes 1 and 6, *C. cellulovorans* Δ*jetABCD* K1; lanes 2 and 7, *C. cellulovorans* Δ*jetABCD* K2; lanes 3 and 8, *C. cellulovorans* Δ*jetABCD* K4; lanes 4 and 9, *C. cellulovorans* 743B wildtype; lanes 5 and 10, pNick_delJetABCD-sg1 plasmid. **b** *C. cellulovorans* 743B chromosomal Wadjet locus. The check primers Del1 and Del2 are indicated with arrows. The confirmed deletion of *C. cellulovorans* Δ*jetABCD* is shown in black

After transconjugation, two colonies obtained were cultivated overnight in selection medium, and subsequently spread onto induction plates containing 10 µg mL^−1^ thiamphenicol for plasmid maintenance and 5 mM theophylline for induction of the *cas9* nickase. After incubation, eight colonies appeared, all with wildtype *jet* genotype according to PCR analysis. Subsequently, these colonies were re-streaked onto induction plates containing thiamphenicol and theophylline, and one clone with a mixed genotype (as determined with PCR using primer pairs resulting in products of different length for the wildtype and the anticipated *jet* mutant genotypes) was obtained. A third round of re-streaking onto induction plates finally resulted in clones with the desired deletion genotype. Further analysis by PCR confirmed the chromosomal deletion of the ORFs Clocel_0831 (*jetA*), Clocel_0832 (*jetB*), Clocel_0833 (*jetC*), Clocel_0834 (*jetD*), which resulted in a 4.3 kb long PCR product for the clean deletion mutant in comparison to the 10.7 kb long fragment for the wildtype. Curing of the deletion plasmid was successful after repeating five times in succession inoculation and growth in medium without antibiotic pressure (Fig. 4). Compared with the wildtype, the growth behavior and cell morphology of the Δ*jetABCD* strain were not affected. Lastly, the deletion was confirmed by Illumina whole-genome sequencing.

**Fig. 5:**
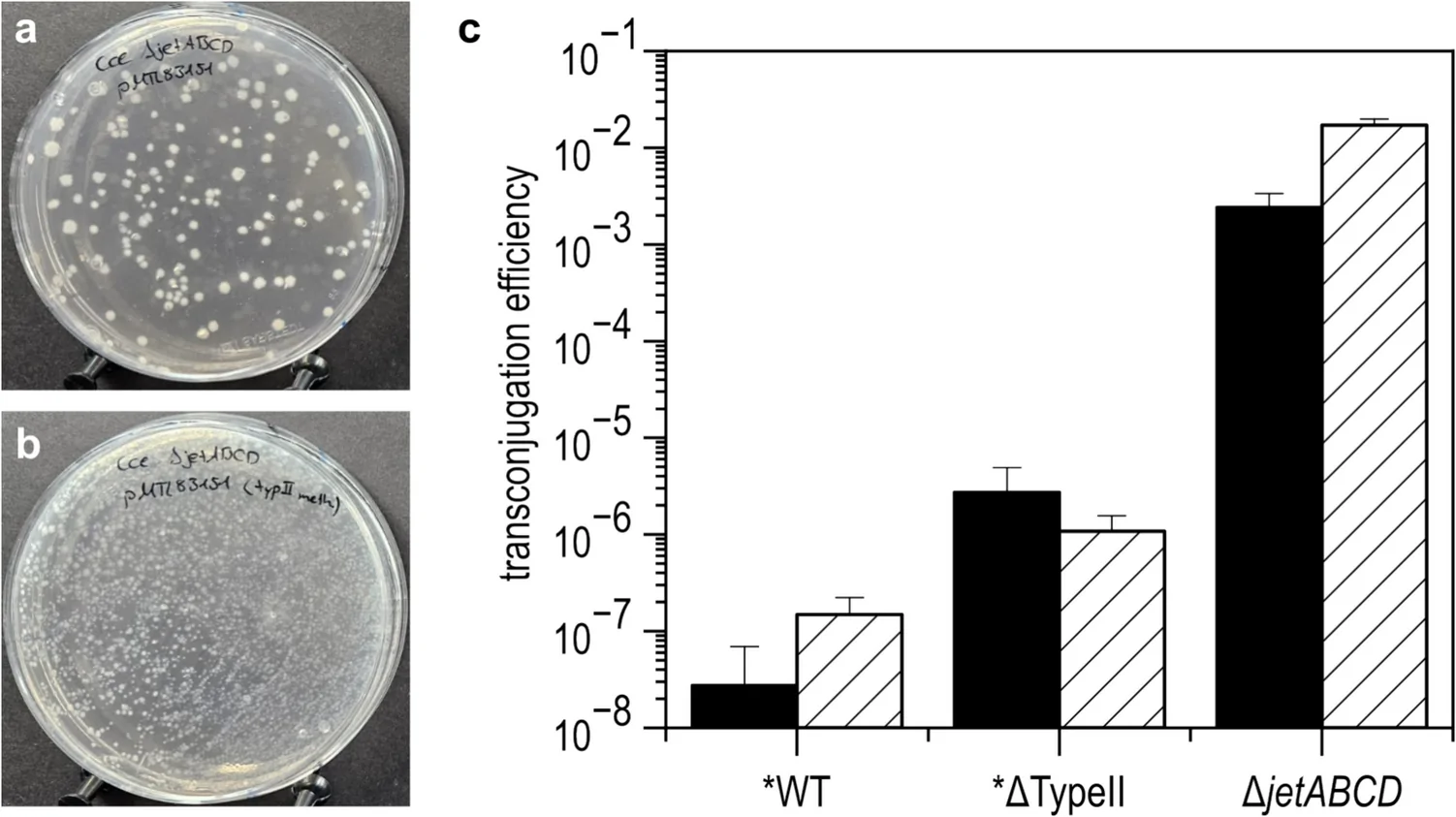
Conjugation efficiency of plasmid pMTL83151 into *C. cellulovorans* Δ*jetABCD*. Panels **a** and **b** show selection plates after mating of pMTL83151-bearing *E. coli* donor strains with *C. cellulovorans* Δ*jetABCD* as recipient. **a** Mating experiment using *E. coli* NEB10B-pMTL85131 (unmethylated plasmid) as donor and *C. cellulovorans* Δ*jetABCD* as recipient. **b** Mating experiments were performed using *E. coli* NEB10B-pMTL85131/placORMI (plasmid methylated in vivo via expression of a *C. cellulovorans-*born methyltransferase of a type II RM system) as donor and *C. cellulovorans* Δ*jetABCD* as recipient. **c** Comparison of the conjugation efficiency of different *C. cellulovorans* strains using unmethylated plasmid (black bar) and methylated plasmid (striped bar). The efficiency values are given as number of transconjugants per recipient cell with standard deviation of five independent experiments for Δ*jetABCD* (*n* = 5, mean ± SD). *Data for WT and ΔTypeII are taken from Schöllkopf et al. (2025). The experimental conditions (see Materials and Methods) were the same in all experiments

The function of the Wadjet system as barrier against incoming plasmid DNA in *C. cellulovorans* was investigated by evaluation of the impact of the *jetABCD* deletion on transconjugation of a recombinant plasmid from *E. coli* donor cells. We have previously shown that the transconjugation efficiency was substantially improved by in vivo methylation in *E. coli* of the shuttle plasmids via expression of a *C. cellulovorans*-born methyltransferase from a type II RM system (Schöllkopf et al. 2025). Therefore, we tested transconjugation of the un-methylated and the in vivo methylated pMTL83151 plasmid (4.5 kb) from the respective *E. coli* donor strains, *E. coli* NEB10B-pMTL85131, or *E. coli* NEB10B-pMTL85131/placORMI, respectively (Fig. 5a and b). A transfer frequency of 2.42 ± 0.94 × 10^−3^ transconjugants per Δ*jetABCD* recipient cell was obtained without previous in vivo methylation (donor strain *E. coli* NEB10B-pMTL85131), corresponding to an increase by approximately five orders of magnitude compared to conjugation of the same plasmid from the *E. coli* donor to wildtype *C. cellulovorans* 743B recipient cells. By employing in vivo methylation (donor strain *E. coli* NEB10B-pMTL85131/placORMI), the transconjugation frequency was further increased to 1.71 ± 0.27 × 10^−2^ transconjugants per Δ*jetABCD* recipient cell (*n* = 5, mean ± SD) (Fig. 5c).

### Wadjet systems in other *Bacillota*

Since Wadjet systems act against the presence of plasmid DNA, the distribution of putative Wadjet system-encoding genes and at the same time the occurrence of natural plasmids in the phylum *Bacillota* (formerly *Firmicutes*) was analyzed. For this purpose, the coding sequences of the JetA, JetB, JetC, and JetD proteins of *Bacillus cereus* Q1 were blasted with tBLASTn against the “RefSeq Genome Database (refseq_genomes)” including “Bacillus/Clostridium group (taxid: 1239),” without “Models” or “uncultured/environmental sample sequences” (data acquisition: August 2024).

**Fig. 6:**
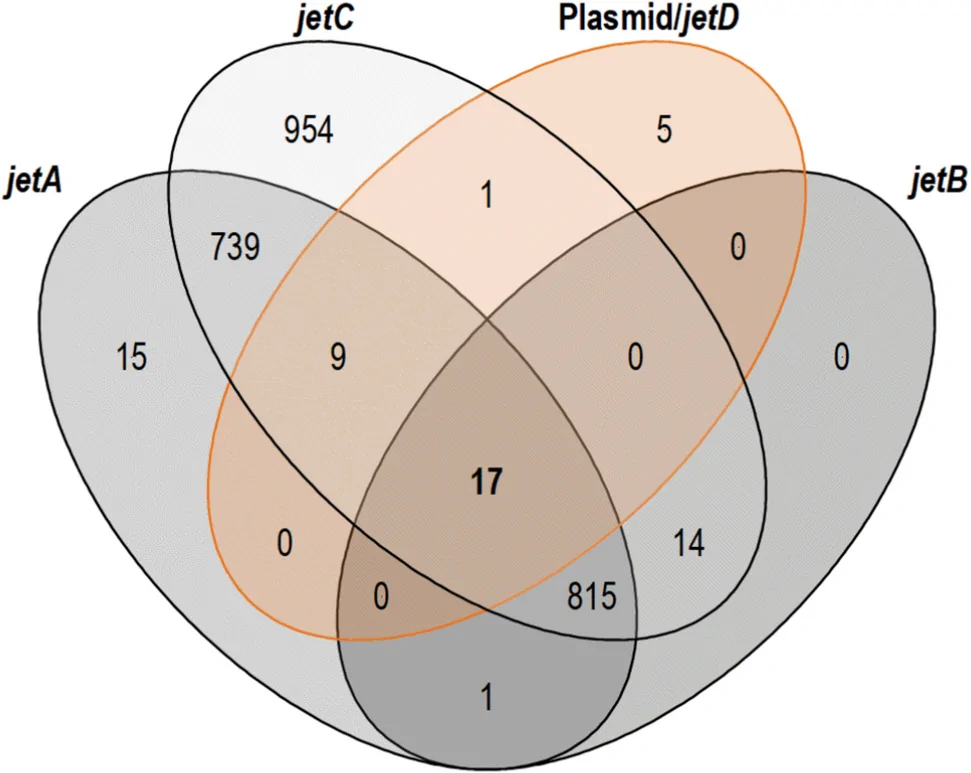
Co-occurrence of *jetA*, *jetB*, *jetC* orthologs and plasmid/*jetD* ortholog-bearing *Bacillota* strains with sequenced genomes

As mentioned before, *jetD* of *Bacillus cereus* Q1 encodes a nuclease essential for the Wadjet-catalyzed plasmid cleavage, so further work was focused on this protein. About 1,442 strains were identified to putatively possess orthologues of *jetD*, while plasmids were identified in only 32 (2.2%) of these strains (supplementary data S3 to S5). Among these plasmid-bearing strains, the plasmid sizes were between 500 kb and 50 kb, i. e. above the suggested Wadjet threshold size of 50–100 kb (Liu et al. 2023), in 16 cases; five *Enterocloster bolteae* strains with a > 40 kb plasmid also carried *jetABCD* genes. Nine of the strains potentially bear plasmids of < 50 kb but lack at least one *jet* gene ortholog. The co-occurrence of the four *jet* genes and plasmids is visualized in Fig. 6, revealing 17 strains bearing *jetABCD* and a plasmid, which are listed in Table S3. *Selenomonas* sp. oral taxon 136 F0591 (NZ_CP014239.1) and *Anaerostipes* sp. PC18 (NZ_CP121164.1) appear to carry relatively small plasmids of 15.6 kb and 1.3 kb, as well as putative *jetABCD* genes. However, in these cases the sequence data was retrieved from metagenomic samples of supragingival dental plaque of a molar tooth, and the intestinal contents of *Mus musculus.* Because these genome sequences were not obtained from axenic clonal isolates, uncertainty remains if these uncultivated strains indeed simultaneously carry chromosomal *jetABCD* genes and small-sized plasmids.

## Discussion

During our genetic engineering work with *C. cellulovorans* 743B we identified a spontaneously occurring mutant strain, *C. cellulovorans* Δ*engE*/2, which had a dramatically improved ability to accept recombinant plasmid DNA from *E. coli* donor strains via conjugation. The occurrence of (usually undesired) mutations like SNPs (single nucleotide polymorphisms), indels, or deletions has been described in different organisms during laboratory work and genetic engineering. Repeated cultivation on artificial, often nutrition-rich media can result in physiological and genetic changes of laboratory strains. Mutations during cultivation can become manifested and detected by sequence analysis when clonal populations are isolated from cultures, e.g., for strain purification or through evolutionary advantages a mutant may have under the given culture conditions. For example, the re-sequencing of a *C. acetobutylicum* ATCC 824 laboratory strain revealed the presence of 177 SNPs, 49 indels, and a 5 kb deletion (Ehsaan et al. 2016). Also, the transposon-mediated disruption of the cellulosomal scaffold gene *cipA* of *C. thermocellum* (Zverlov et al. 2008) or an acquired thymidine auxotrophy caused by SNPs in *C. botulinum* Group II strain Beluga (Selby et al. 2025) were reported under standard cultivation conditions. On the other hand, powerful laboratory methods have been developed that exploit the ability of spontaneous adaptation of organisms of interest under controlled conditions in bioreactors and allow the isolation of mutants with favorable properties by the targeted application of appropriate selective conditions. Recently, this strategy called adaptive laboratory evolution (ALE) was applied to a genetically engineered *C. cellulovorans* strain to improve its butanol tolerance (Wen et al. 2019).

Spontaneous mutations can lead to unexpected outcomes, like in our case where strain construction experiments using a Cas9-based chromosome editing approach to generate a “clean” deletion strain lacking the ORF Clocel_3559. This experiment led to the discovery of a mutant strain with strongly improved genetic accessibility. Genome sequence analysis of *C. cellulovorans* Δ*engE*/2 revealed three large chromosomal deletions with sizes between about 17 kb and 129 kb*.* Presumably very significant selective pressure was necessary to accumulate three individual deletions at vastly different locations of the chromosome (Fig. 1). Different factors may have contributed to selection of the genotype observed in *C. cellulovorans* Δ*engE*/2: (1) forced plasmid maintenance by the selection pressure by antibiotic resistant marker, (2) isolating a single colony growing after the transconjugation event, (3) cultivation in nutrition-rich medium for fast cell growth, and (4) using a Cas9-based genome editing approach which is known for targeted strand breaks, but also unwanted off-target effects during the chromosomal editing process (Richter et al. 2017). Additionally, the expression of Cas9 remained ongoing (while due to Cas9 toxicity presumably aiding in plasmid loss) throughout the time needed for plasmid curing to finalize the chromosome editing procedure.

Interestingly, the three chromosomal deletions with sizes of about 17 kb, 23 kb, and 129 kb found in strain Δ*engE*/2 encompassed genes putatively connected with a CRISPR-Cas system, a Wadjet system, and a type II RM system, respectively. All of these mutations could contribute to the strain’s phenotype of enhanced acceptor potency for DNA transconjugation from *E. coli.* The largest deletion (deletion A in Fig. 1) included the loss of a type II RM system. Clearly, RM systems in general protect bacterial cells from incoming foreign DNA (Minton et al. 2016). Thus, the spontaneous deletion of the type II RM system in strain *C. cellulovorans* Δ*engE*/2 could have facilitated the conjugative transfer of the Cas9 vector used in the chromosome editing procedure after which the Δ*engE*/2 strain was identified. In different experiments carried out previous to and independent of this study, we have recently characterized this type II RM system (Schöllkopf et al. 2025). In that study, we found that a *C. cellulovorans* 743B variant that lacked only this type II RM system displayed a 7.3-fold improved plasmid uptake frequency compared with the wildtype when the plasmid was not methylated in the *E. coli* donor in a *C. cellulovorans* 743B-specific way, and an about 110-fold improved uptake frequency when *C. cellulovorans-*specifically methylated plasmid was transferred (Schöllkopf et al. 2025). On its own, however, the deletion of this RM system cannot account for the massive increase in transconjugation efficiency with recipient strain *C. cellulovorans* Δ*engE*/2 as seen in Fig. 1b.

Apart from the deleted type II RM system, another spontaneous chromosomal deletion of 17 kb (deletion B in Fig. 1) resulted in the loss of Clocel_2327 and Clocel_2328 of the putative CRISPR system, another defense system directed against foreign DNA. This deletion was probably acquired independently from the other two deletions A and C. As demonstrated by PCR-based monitoring of the presence of this chromosomal locus during a series of repeated consecutive subcultures, this region of the *C. cellulovorans* 743B chromosome appears to be genetically unstable, at least under the cultivation conditions used here. The *cas* region’s presence was confirmed in early cultures, starting with a culture that had been stored away at − 80 °C after having been received from the DSMZ culture collection, but after around six consecutive sub-cultivations the *cas* region was spontaneously lost (Fig. 2). Recent studies revealed and discussed the occurrence of self-targeting spacers in natural CRISPR-Cas systems, which are expected to target the cell’s own DNA but on the other hand also could have a beneficial effect for the host’s evolution (reviewed by Wimmer & Beisel 2019). In some cases, cells may survive a self-targeting spacer-induced auto-immune reaction by disruption of the CRISPR-Cas locus, but large deletions up to 40–100 kb can be the consequence (Vercoe et al. 2013). Because the targets of the CRISPR array spacers in *C. cellulovorans* are not clear, this effect cannot be ruled out. Interestingly, the deleted *cas* region in Δ*engE*/2 is flanked by 5 bp direct repeat sequences. In a 5 kb deletion found by resequencing of the *C. acetobutylicum* ATCC 824 genome, a 10 bp repetitive pattern was identified that was proposed to have mediated the deletion (Ehsaan et al. 2016). Due to the rapid spontaneous loss of the *cas* locus, it was not possible to investigate in detail the impact of the putative CRISPR system on the genetic accessibility of *C. cellulovorans* 743B.

The third large deletion found by sequencing of the *C. cellulovorans* Δ*engE*/2 genome, a 23 kb large chromosomal region (deletion C in Fig. 1), included the four clustered genes *jetABCD* comprising a putative Wadjet system. Interestingly, already some years ago, a *Mycobacterium smegmatis* strain with improved plasmid uptake property (non-natural uptake via electroporation) was reported to have a mutation in EptC or EptABCD (MksBEFG) (Panas et al. 2014), which is homolog to JetABCD (Liu et al. 2023). Markerless deletion of only the *jetABCD* genes on the *C. cellulovorans* 743B chromosome revealed a huge effect of this putative Wadjet gene cluster on the efficiency of plasmid DNA uptake via conjugation (Fig. 5). Our results demonstrate that of all three regions and their respective genes missing in the *C. cellulovorans* Δ*engE*/2 chromosome, the loss of the Wadjet-encoding genes *jetABCD* within deletion C in particular was decisive to effect an increase in transconjugation efficiency by several orders of magnitude.

Horizontal gene transfer (HGT) is a major driver in evolution of prokaryotes. The effect of the *jetABCD* genes on plasmid transconjugation from *E. coli* to *C. cellulovorans* (about 5-log lower conjugation frequency in the presence of *jetABCD* than without) was much larger than the effect of the *jet* genes on a different type of HGT, natural transformation in *B. subtilis* with plasmid DNA. In this artificial system based on a *B. subtilis* strain heterologously expressing foreign *jetABCD* genes, the transformation efficiency for the uptake of externally provided plasmid DNA by *B. subtilis* BEST7003 was about 2 orders of magnitude lower in the presence of the *B. cereus* Q1 *jetABCD* genes than without (Doron et al. 2018). This difference in apparent efficiency of the Wadjet systems in *C. cellulovorans* and recombinant *B. subtilis* may have various reasons. The apparently lower efficiency in *B. subtilis* may be due to sub-optimal expression of the heterologous *B. cereus jetABCD* genes in the study with *B. subtilis* as the host. Moreover, natural transformation is generally known to be less effective for plasmid uptake in *B. subtilis*, which may also play a role (Jeong et al. 2022). Furthermore, it must be considered that different plasmids were used in both studies. In addition, the mechanisms by which circular double-stranded DNA plasmids are established upon single-stranded DNA uptake, either by conjugation or natural transformation, may differ. These differences could influence how effectively the Wadjet system defends against invasive plasmid DNA. It may be rewarding in future to study the impact of having a Wadjet system on the uptake of plasmid DNA by both mechanisms of HGT in the same host organism and using the same plasmid backbone and same copy number. *C. cellulovorans* is not suited for such studies because it is not naturally transformable.

In silico analysis of the available genome sequence data of low-GC-Gram-positive bacteria revealed that the co-occurrence of the Wadjet genes (*jetABCD*) and plasmids in the phylum *Bacillota* (formerly *Firmicutes*) is restricted to only some strains carrying large plasmids (see supplementary information). Merely 2 of 1,442 genomes suggest the co-existence of all four Wadjet-encoding genes and small plasmids (below 16 kb in size), but in these instances the genomes were not from pure cultures and therefore may be questionable. In those strains having a complete set of Wadjet genes and simultaneously large plasmids, the apparent plasmid size cut-off was about 40 kb. In contrast, results from biochemical experiments about the function of the Wadjet system from *Bacillus cereus* or *Bacillus thuringiensis* on circular plasmids estimated a plasmid cut-off size about > 50–100 kb (Liu et al. 2023). Irrespective of the precise cut-off size, the absence of small plasmids in the vast majority of Wadjet-encoding *Bacillota* genomes indicates a high efficiency of Wadjet systems in shielding off invasive small-sized plasmids.

Small plasmids are widespread in bacteria and can carry genes with beneficial properties, for example conferring antibiotic or heavy metal resistance, and thereby can provide a selection advantage under hostile environmental conditions. On the other hand, all extrachromosomal DNA, if beneficial or not, replicates by use of the cellular enzyme machinery, metabolites and energy, which imposes a chronic metabolic burden upon cells. The fitness cost of maintaining plasmids can lead to their rapid loss in the absence of selective pressure and may limit the size of the plasmid-containing population (Bravo 2024). Presumably, the highly variable conditions and in consequence the selective constraints in place in different habitats ultimately determine if small plasmids with their gene content are beneficial, or not, or even a risk or an intolerable burden, in which case the possession of a Wadjet system may be of advantage.

The near-complete absence of small- and medium-sized plasmids in Wadjet system-containing *Bacillota* strains suggests a very high stringency of Wadjet defense systems with regard to their efficiency in preventing the acquisition and maintenance of such plasmids. The low number or even absence of small plasmids in Wadjet system-containing *Bacillota* strains indicates that evasion mechanisms against these systems may be scarce. In the case of RM defense systems, internalized foreign plasmids can evade restriction if they do not contain the endonuclease recognition sequences specific for the RM system, if they are methylated before restriction takes place, or if an anti-restriction protein is present. Wadjet systems in contrast to RM systems do not recognize specific nucleotide sequences (Liu et al. 2023), and to date, no evasion mechanisms are known how plasmids could avoid cleavage by Wadjet–apart from, importantly, plasmid size.

Wadjet systems were initially discovered through JetABCD expression in a heterologous host and in vitro biochemical analysis revealed their molecular mode of action. However, prior to this study, very little was known about their in vivo function in bacteria that naturally equipped with Wadjet systems, in particular, how effectively they protect against invasive small- or medium-sized circular DNA in their native cellular background. Our study now for the first time demonstrates how extraordinarily efficient a Wadjet system in its native host *C. cellulovorans* can block the establishment of a plasmid transferred into its cells via conjugation. To this end, inactivation of the Wadjet system in this study had a much larger effect on improving transconjugation than the RM systems also present in *C. cellulovorans* 743B (Almeida et al. 2025; Schöllkopf et al. 2025). In conclusion, this work should embolden to think beyond familiar defense systems. In addition to RM systems, Wadjet-type anti-plasmid systems could represent important targets for inactivation in order to improve plasmid uptake in the course of “domestication” of bacterial strains for their use in future biotechnological applications. The in silico identification of the genes for such systems in the genome and their subsequent removal appears to be a good strategy to obtain improved strains which are genetically more easily tractable for further metabolic engineering.

## Supplementary Information

Below is the link to the electronic supplementary material.

ESM 1 (DOCX 1.13 MB)

## Data Availability

The data are given in the text and the supplemented files.
