# Supplementary material for: SMC-like Wadjet system prevents plasmid transfer into Clostridium cellulovorans
Source: Appl Microbiol Biotechnol. 2025 Jul 23;109:170. doi: 10.1007/s00253-025-13551-w (PMC12287153; doi:10.1007/s00253-025-13551-w)
Supplement: Supplementary file 1 [file 253_2025_13551_MOESM1_ESM.docx]

**Supplementary information**

**S1** - Synthetic DNA fragment

CACCGCCCATCCGACGCTATTTGTGCCGATAGCTAAGCCTATTGAGTATTTCTTATCCATATGGTTGCCTCCTTAGCAGGGTGCTGCCAAGGGCATCAAGACGATGCTGGTATCACCGGTACCAATAAATTGTGGTAATTAACCAACTCTATTTTATCATGTATTCCAATGTTTTTAAAGTATTTTGTGATATGCAAATGCGGCCGCGTAAAACGACGGCCAGTTTGACAGCTAGCTCAGTCCTAGGTATAATACTAGTGAAACTTGACTGGTACAGGTTGGTTAGAGCTAGAAATAGCAAGTTAAAATAAGGCTAGTCCGTTATCAACTTGAAAAAGTGGCACCGAGTCGGTGCTTTTTTTCTCGAGATAAAA

**S2** - Alignment of JetD amino acid sequence segments. (a) The protein sequences of JetD proteins from *Bacillus cereus* Q1, *Pseudomonas aeruginosa* PA14, *C. cellulovorans* 734B, *Clostridium kluyveri* JZZ, and *Clostridium beijerincki* DJ123 were aligned using Jalview's Muscle algorithm with default settings. Conserved amino acids are shown in blue, and the experimentally confirmed essential residues of the JetD enzymes from *Bacillus cereus* Q1 and *Pseudomonas aeruginosa* PA14 are indicated with arrows and labeled with the respective amino acid residue from *C. cellulovorans*. (b) AlphaFold prediction of JetD of *C. cellulovorans*. The degree of amino acid sequence conservation is indicated by a color gradient from blue (low) to red (high). (c) Putative catalytic center of JetD from *C. cellulovorans*. (d) Structural alignment of AlphaFold-predicted JetD orthologs from *C. cellulovorans* (blue), *Clostridium kluyveri* (green), and *Bacillus cereus* (orange). (e) Structural alignment of AlphaFold-predicted JetD from C. cellulovorans (blue) with the cryo-EM structure of the JetD homodimer from Pseudomonas aeruginosa (PDB ID: 7TIL, Deep et al., 2022). Chain A and Chain B of the homodimer are shown in orange and grey, respectively. It should be noted that current structural prediction tools appear to be inefficient regarding multimeric proteins

**S3** - List of *jetABCD* ortholog-bearing strains and sizes of their plasmids. The data were compiled from Table S3 to provide a condensed overview of *Bacillota* genomes with co-existence of all four jet orthologs and plasmids, and their plasmid sizes

| **Accession no.** | **Organism** | **Strain** | **Plasmid size** |  |
| --- | --- | --- | --- | --- |
|  |  |  |  |  |
| NZ_CP036057.1 | *Bacillus mycoides* | JAS06/1 | 487 kb |  |
| NZ_CP072061.1 | *Bacillus mycoides* | JAS06/1 | 487 kb |  |
| NZ_CP101135.1 | *Bacillus paranthracis* | Bt C4 | 292 kb |  |
| NZ_CP085413.1 | *Bacillus paranthracis* | KF11 | 286 kb |  |
| NZ_CABKQO010000005.1 | *Enterocloster clostridioformis* | isolate MGYG-HGUT-01386 | 192 kb |  |
| NZ_CP051681.1 | *Cohnella herbarum* | MFER-1 | 174 kb |  |
| NZ_LZZC01000007.1 | *Clostridium beijerinckii* | DSM 791 | 73 kb |  |
| NZ_CP073653.1 | *Clostridium beijerinckii* | DSM 791 | 73 kb |  |
| NZ_CP015607.1 | *Bacillus safensis* | U14-5 | 58 kb |  |
| NZ_CP053229.1 | *Enterocloster bolteae* | CBBP-2 | 51 kb |  |
| NZ_DS480690.1 | *Enterocloster bolteae* | ATCC BAA-613 | 43 kb |  |
| NZ_CABKUK010000001.1 | *Enterocloster bolteae* | isolate MGYG-HGUT-01493 | 43 kb |  |
| NZ_PUEM01000006.1 | *Enterocloster bolteae* | ATCC BAA-613 | 43 kb |  |
| NZ_CP022464.2 | *Enterocloster bolteae* | ATCC BAA-613 | 43 kb |  |
| NZ_CP069520.1 | *Enterocloster bolteae* | FDAARGOS_1231 | 43 kb |  |
| NZ_CP014239.1 | *Selenomonas* sp. oral taxon 136 | F0591 | 15 kb |  |
| NZ_CP121164.1 | *Anaerostipes* sp. | PC18 | 13 kb |  |

**S4** – List of strains from the phylum *Bacillota* with available genome sequences, which carry a *jetD* ortholog and a plasmid. The plasmid size is given in base pairs (bp), and the presence/absence of *jetA*, *jetB*, *jetC* and *jetD* orthologs is indicated (+/-).

| **Accession no.** | **Organism** | **Strain** | **Plasmid**, (+/-) | **Plasmid** **size**, bp |  | **Jet orthologs**, (+/-) | | | |
| --- | --- | --- | --- | --- | --- | --- | --- | --- | --- |
|  |  |  |  |  |  | **JetA** | **JetB** | **JetC** | **JetD** |
| NZ_CP036057.1 | *Bacillus mycoides* | JAS06/1 | + | 487389 |  | + | + | + | + |
| NZ_CP072061.1 | *Bacillus mycoides* | JAS06/1 | + | 487389 |  | + | + | + | + |
| NC_014389.1 | *Butyrivibrio proteoclasticus* | B316 | + | 361399 |  | - | - | + | + |
| NZ_CP101135.1 | *Bacillus paranthracis* | Bt C4 | + | 292347 |  | + | + | + | + |
| NZ_CP085413.1 | *Bacillus paranthracis* | KF11 | + | 286462 |  | + | + | + | + |
| NZ_JAJCHR010000017.1 | *Agathobacter rectalis* | DFI.5.28 | + | 251725 |  | - | - | - | + |
| NZ_CP143947.1 | *Agathobacter rectalis* | DFI.5.28 | + | 251725 |  | - | - | - | + |
| NZ_CP011512.1 | *Paenibacillus peoriae* | HS311 | + | 213282 |  | - | - | - | + |
| NZ_CABKQO010000005.1 | *Enterocloster clostridioformis* |  | + | 192394 |  | + | + | + | + |
| NZ_CP051681.1 | *Cohnella herbarum* | MFER-1 | + | 174538 |  | + | + | + | + |
| NZ_CP066339.1 | *Alicyclobacillus* sp. | SO9 | + | 90030 |  | + | - | + | + |
| NZ_LZZC01000007.1 | *Clostridium beijerinckii* | DSM 791 | + | 73345 |  | + | + | + | + |
| NZ_CP073653.1 | *Clostridium beijerinckii* | DSM 791 | + | 73345 |  | + | + | + | + |
| NZ_CP015607.1 | *Bacillus safensis* | U14-5 | + | 58536 |  | + | + | + | + |
| NZ_CP157265.1 | *Paenibacillus peoriae* | MHJL1 | + | 52026 |  | - | - | - | + |
| NZ_CP053229.1 | *Enterocloster bolteae* | CBBP-2 | + | 51398 |  | + | + | + | + |
| NZ_CP071182.1 | *Alicyclobacillus mengziensis* | S30H14 | + | 47863 |  | + | - | + | + |
| NZ_DS480690.1 | *Enterocloster bolteae* | ATCC BAA-613 | + | 43860 |  | + | + | + | + |
| NZ_CABKUK010000001.1 | *Enterocloster bolteae* |  | + | 43860 |  | + | + | + | + |
| NZ_PUEM01000006.1 | *Enterocloster bolteae* | ATCC BAA-613 | + | 43860 |  | + | + | + | + |
| NZ_CP022464.2 | *Enterocloster bolteae* | ATCC BAA-613 | + | 43860 |  | + | + | + | + |
| NZ_CP069520.1 | *Enterocloster bolteae* | FDAARGOS_1231 | + | 43772 |  | + | + | + | + |
| NZ_CP082837.1 | *Sporolactobacillus terrae* | EFEL7003 | + | 17307 |  | - | - | - | + |
| NZ_CP144221.1 | *Natranaerobius thermophilus* | JW/NM-WN-LF | + | 17207 |  | + | - | + | + |
| NC_010718.1 | *Natranaerobius thermophilus* | JW/NM-WN-LF | + | 17207 |  | + | - | + | + |
| NZ_CP014239.1 | *Selenomonas* sp. oral taxon 136 | F0591 | + | 15626 |  | + | + | + | + |
| NZ_CABHNE010000053.1 | *Mediterraneibacter gnavus* |  | + | 6945 |  | + | - | + | + |
| NZ_CABKQB010000017.1 | *Mediterraneibacter gnavus* |  | + | 6945 |  | + | - | + | + |
| NZ_BAABSA010000015.1 | *Mediterraneibacter gnavus* | NBRC 114413 | + | 6945 |  | + | - | + | + |
| NZ_BSQR01000015.1 | *Mediterraneibacter gnavus* | NBRC 114413 | + | 6945 |  | + | - | + | + |
| NZ_CP084014.1 | *Mediterraneibacter gnavus* | NBRC 114413 | + | 6945 |  | + | - | + | + |
| NZ_CP121164.1 | *Anaerostipes* sp. | PC18 | + | 1337 |  | + | + | + | + |
| NZ_LEBY01000070.1 | *Bacillus paranthracis* | AH827 | - | 0 |  | + | + | + | + |

**S5 –** List of strains from the phylum *Bacillota* with available genome sequences, which carry a *jetD* ortholog but no plasmid.

| **Accession no.** | **Organism** | **Strain** |
| --- | --- | --- |
| NZ_CP102272.1 | *[Clostridium] asparagiforme* DSM 15981 | DSM 15981 |
| NZ_GG657592.1 | *[Clostridium] asparagiforme* DSM 15981 | DSM 15981 |
| NZ_JH376874.1 | *[Clostridium] clostridioforme* 2_1_49FAA | 2_1_49FAA |
| NZ_KB851112.1 | *[Clostridium] clostridioforme 90A1* | 90A1 |
| NZ_KB851056.1 | *[Clostridium] clostridioforme 90A3* | 90A3 |
| NZ_KB851074.1 | *[Clostridium] clostridioforme 90A4* | 90A4 |
| NZ_KB851023.1 | *[Clostridium] clostridioforme 90A6* | 90A6 |
| NZ_KB851019.1 | *[Clostridium] clostridioforme 90A8* | 90A8 |
| NZ_KB850969.1 | *[Clostridium] clostridioforme 90B1* | 90B1 |
| NZ_AUJR01000031.1 | *[Clostridium] clostridioforme AGR2157* | AGR2157 |
| NZ_KB850940.1 | *[Clostridium] clostridioforme CM201* | CM201 |
| NZ_KQ235921.1 | *[Clostridium] clostridioforme WAL-7855* | WAL-7855 |
| NZ_FOHN01000028.1 | *[Clostridium] polysaccharolyticum* | DSM 1801 |
| NZ_AP024846.1 | *[Clostridium] scindens* | G10 |
| NZ_AP025569.1 | *[Clostridium] scindens* | CE91-St59 |
| NZ_AP025570.1 | *[Clostridium] scindens* | CE91-St60 |
| NZ_CABKNF010000030.1 | *[Clostridium] scindens* |  |
| NZ_CP045695.1 | *[Clostridium] scindens* | BL389WT3D |
| NZ_CP069444.1 | *[Clostridium] scindens* | FDAARGOS_1227 |
| NZ_CP137819.1 | *[Clostridium] scindens* | I10 |
| NZ_JAAISO010000027.1 | *[Clostridium] scindens* | MSK.1.26 |
| NZ_JAAISP010000026.1 | *[Clostridium] scindens* | MSK.1.16 |
| NZ_JABFCG010000027.1 | *[Clostridium] scindens* | GGCC_0168 |
| NZ_JAQDIV010000025.1 | *[Clostridium] scindens* | AM05-22 |
| NZ_JAQDJC010000026.1 | *[Clostridium] scindens* | AM07-30 |
| NZ_JBBNPN010000012.1 | *[Clostridium] scindens* | CLA-SR-H017 |
| NZ_JBFQGJ010000001.1 | *[Clostridium] scindens* | S077 |
| NZ_JBFQGK010000001.1 | *[Clostridium] scindens* | JCM10422 (TH-82) |
| NZ_VUMB01000031.1 | *[Clostridium] scindens* | BL-389-WT-3D |
| NZ_CP036170.1 | *[Clostridium] scindens ATCC 35704* | ATCC 35704 |
| NZ_DS499689.1 | *[Clostridium] scindens ATCC 35704* | ATCC 35704 |
| NZ_JAAISL010000058.1 | *[Clostridium] symbiosum* | MSK.7.21 |
| NZ_JAJCJH010000011.1 | *[Clostridium] symbiosum* | DFI.7.73 |
| NZ_JAJCMB010000027.1 | *[Clostridium] symbiosum* | DFI.5.64 |
| NZ_QSDB01000055.1 | *[Clostridium] symbiosum* | OF01-1AC |
| NZ_CABKOA010000025.1 | *[Ruminococcus] lactaris* |  |
| NZ_JADMSD010000025.1 | *[Ruminococcus] lactaris* | D43t1_170807_H11 |
| NZ_JAJBME010000020.1 | *[Ruminococcus] lactaris* | MSK.16.42 |
| NZ_JAJBNO010000009.1 | *[Ruminococcus] lactaris* | MSK.17.86 |
| NZ_JAJBNP010000016.1 | *[Ruminococcus] lactaris* | MSK.17.83 |
| NZ_JAJBNT010000018.1 | *[Ruminococcus] lactaris* | MSK.16.1 |
| NZ_JAJBQI010000017.1 | *[Ruminococcus] lactaris* | MSK.17.62 |
| NZ_JAJBRQ010000020.1 | *[Ruminococcus] lactaris* | MSK.18.80 |
| NZ_JAJBRR010000020.1 | *[Ruminococcus] lactaris* | MSK.18.64 |
| NZ_JAJBRS010000019.1 | *[Ruminococcus] lactaris* | MSK.18.54 |
| NZ_JAJBRU010000018.1 | *[Ruminococcus] lactaris* | MSK.18.24 |
| NZ_JAJBRX010000017.1 | *[Ruminococcus] lactaris* | MSK.17.77 |
| NZ_JAJBRY010000009.1 | *[Ruminococcus] lactaris* | MSK.17.22 |
| NZ_JAJBRZ010000017.1 | *[Ruminococcus] lactaris* | MSK.17.20 |
| NZ_JAKNIF010000010.1 | *[Ruminococcus] lactaris* | MSK.17.16 |
| NZ_JAQDJO010000035.1 | *[Ruminococcus] lactaris* | AM09-20-2 |
| NZ_JAQEAN010000013.1 | *[Ruminococcus] lactaris* | AM25-13AC |
| NZ_JAQEAQ010000009.1 | *[Ruminococcus] lactaris* | AM25-24 |
| NZ_JAQENN010000050.1 | *[Ruminococcus] lactaris* | TM13-2A |
| NZ_JARFIM010000021.1 | *[Ruminococcus] lactaris* | DFI.5.31 |
| NZ_JBBNJM010000043.1 | *[Ruminococcus] lactaris* | CLA-AA-H279 |
| NZ_JBBNKL010000013.1 | *[Ruminococcus] lactaris* | CLA-AA-H87 |
| NZ_QRHG01000014.1 | *[Ruminococcus] lactaris* | AM25-1LB |
| NZ_QRMI01000006.1 | *[Ruminococcus] lactaris* | AM09-9 |
| NZ_QSQN01000013.1 | *[Ruminococcus] lactaris* | TF11-7 |
| NZ_CP102292.1 | *[Ruminococcus] lactaris ATCC 29176* | ATCC 29176 |
| NZ_DS990185.1 | *[Ruminococcus] lactaris ATCC 29176* | ATCC 29176 |
| NZ_KI669407.1 | *[Ruminococcus] lactaris CC59_002D* | CC59_002D |
| NZ_AP028249.1 | *[Ruminococcus] torques* | 17EGH124 |
| NZ_CABHNA010000057.1 | *[Ruminococcus] torques* |  |
| NZ_CABJEY010000003.1 | *[Ruminococcus] torques* |  |
| NZ_CYZO01000034.1 | *[Ruminococcus] torques* | 2789STDY5834841 |
| NZ_CZBR01000004.1 | *[Ruminococcus] torques* | 2789STDY5608833 |
| NZ_CZBS01000010.1 | *[Ruminococcus] torques* | 2789STDY5608867 |
| NZ_CZBX01000005.1 | *[Ruminococcus] torques* | 2789STDY5834889 |
| NZ_JAJCIF010000020.1 | *[Ruminococcus] torques* | DFI.3.129 |
| NZ_JAJCMQ010000019.1 | *[Ruminococcus] torques* | DFI.4.158 |
| NZ_JAJCPV010000013.1 | *[Ruminococcus] torques* | DFI.1.203 |
| NZ_JAKNDF010000013.1 | *[Ruminococcus] torques* | DFI.1.111 |
| NZ_JAKNDF010000309.1 | *[Ruminococcus] torques* | DFI.1.111 |
| NZ_JAKNEW010000020.1 | *[Ruminococcus] torques* | DFI.4.159 |
| NZ_JAKNFB010000022.1 | *[Ruminococcus] torques* | DFI.4.27 |
| NZ_JAKNIZ010000020.1 | *[Ruminococcus] torques* | MSK.8.28 |
| NZ_JANGDZ010000002.1 | *[Ruminococcus] torques* | DFI.1.248 |
| NZ_JANGEP010000016.1 | *[Ruminococcus] torques* | DFI.1.118 |
| NZ_JANGEQ010000002.1 | *[Ruminococcus] torques* | DFI.1.117 |
| NZ_JANGER010000015.1 | *[Ruminococcus] torques* | DFI.1.116 |
| NZ_JANGES010000002.1 | *[Ruminococcus] torques* | DFI.1.115 |
| NZ_JANGET010000002.1 | *[Ruminococcus] torques* | DFI.1.114 |
| NZ_JANGEV010000015.1 | *[Ruminococcus] torques* | DFI.1.112 |
| NZ_JAQCOZ010000017.1 | *[Ruminococcus] torques* | AF04-18B |
| NZ_JAQDRP010000019.1 | *[Ruminococcus] torques* | AM109-74 |
| NZ_JAQEBM010000022.1 | *[Ruminococcus] torques* | AM28-7 |
| NZ_JAQECH010000037.1 | *[Ruminococcus] torques* | AM38-11BH |
| NZ_JARFIG010000023.1 | *[Ruminococcus] torques* | DFI.4.90 |
| NZ_JBBJJQ010000018.1 | *[Ruminococcus] torques* | VIII-239 |
| NZ_JBBNIY010000018.1 | *[Ruminococcus] torques* | CLA-AA-H261 |
| NZ_JBBNPG010000016.1 | *[Ruminococcus] torques* | CLA-SR-H010 |
| NZ_QRIH01000002.1 | *[Ruminococcus] torques* | AM22-16 |
| NZ_RCYR01000009.1 | *[Ruminococcus] torques* | aa_0143 |
| NZ_WMZY01000013.1 | *[Ruminococcus] torques* | BIOML-A7 |
| NZ_WMZZ01000013.1 | *[Ruminococcus] torques* | BIOML-A6 |
| NZ_WNAA01000009.1 | *[Ruminococcus] torques* | BIOML-A5 |
| NZ_WNAB01000016.1 | *[Ruminococcus] torques* | BIOML-A4 |
| NZ_WNAC01000009.1 | *[Ruminococcus] torques* | BIOML-A3 |
| NZ_WNAD01000010.1 | *[Ruminococcus] torques* | BIOML-A2 |
| NZ_WNAE01000005.1 | *[Ruminococcus] torques* | BIOML-A1 |
| NZ_DS264366.1 | *[Ruminococcus] torques ATCC 27756* | ATCC 27756 |
| NC_021015.1 | *[Ruminococcus] torques L2-14* | L2-14 |
| NZ_CP092643.1 | *Agathobacter rectalis* | VPI 0990 |
| NZ_CP100127.1 | *Agathobacter rectalis* | NB2A-17-FMU |
| NZ_CVRQ01000023.1 | *Agathobacter rectalis* | T1-815 |
| NZ_CYXM01000001.1 | *Agathobacter rectalis* | 2789STDY5834968 |
| NZ_CYYW01000003.1 | *Agathobacter rectalis* | 2789STDY5608860 |
| NZ_CZAJ01000024.1 | *Agathobacter rectalis* | 2789STDY5834884 |
| NZ_JAAILW010000005.1 | *Agathobacter rectalis* | MSK.17.79 |
| NZ_JAAILX010000005.1 | *Agathobacter rectalis* | MSK.17.78 |
| NZ_JAAILY010000005.1 | *Agathobacter rectalis* | MSK.17.70 |
| NZ_JAAIMC010000005.1 | *Agathobacter rectalis* | MSK.17.57 |
| NZ_JAAIMD010000007.1 | *Agathobacter rectalis* | MSK.17.52 |
| NZ_JAAIME010000011.1 | *Agathobacter rectalis* | MSK.17.42 |
| NZ_JAAIMG010000005.1 | *Agathobacter rectalis* | MSK.17.3 |
| NZ_JAAIMJ010000005.1 | *Agathobacter rectalis* | MSK.17.19 |
| NZ_JAAIMK010000005.1 | *Agathobacter rectalis* | MSK.17.13 |
| NZ_JAAIMP010000004.1 | *Agathobacter rectalis* | MSK.16.45 |
| NZ_JAAIMQ010000004.1 | *Agathobacter rectalis* | MSK.16.22 |
| NZ_JAAIRZ010000006.1 | *Agathobacter rectalis* | MSK.9.15 |
| NZ_JAAISA010000004.1 | *Agathobacter rectalis* | MSK.9.13 |
| NZ_JAAISB010000009.1 | *Agathobacter rectalis* | MSK.22.51 |
| NZ_JAAISC010000016.1 | *Agathobacter rectalis* | MSK.22.39 |
| NZ_JAAISD010000008.1 | *Agathobacter rectalis* | MSK.22.28 |
| NZ_JAAISE010000009.1 | *Agathobacter rectalis* | MSK.22.23 |
| NZ_JAAISF010000010.1 | *Agathobacter rectalis* | MSK.22.19 |
| NZ_JAAISG010000009.1 | *Agathobacter rectalis* | MSK.22.12 |
| NZ_JAAISH010000012.1 | *Agathobacter rectalis* | MSK.13.59 |
| NZ_JAAISI010000003.1 | *Agathobacter rectalis* | MSK.13.50 |
| NZ_JAAISJ010000011.1 | *Agathobacter rectalis* | MSK.13.48 |
| NZ_JAAISK010000011.1 | *Agathobacter rectalis* | MSK.13.47 |
| NZ_JADMPC010000003.1 | *Agathobacter rectalis* | D31t1_170403_H3 |
| NZ_JADMQZ010000028.1 | *Agathobacter rectalis* | D43t1_170807_G2 |
| NZ_JADNBN010000014.1 | *Agathobacter rectalis* | 1001271B_150615_F12 |
| NZ_JADNCL010000009.1 | *Agathobacter rectalis* | J1101437_171009_H3 |
| NZ_JADNGW010000045.1 | *Agathobacter rectalis* | D54t1_190329_G12 |
| NZ_JADNIT010000025.1 | *Agathobacter rectalis* | D41t1_190614_G3 |
| NZ_JADNON010000011.1 | *Agathobacter rectalis* | D53t1_180928_G2 |
| NZ_JADPAO010000021.1 | *Agathobacter rectalis* | 1001283B150210_160208_G9 |
| NZ_JAJCJK010000015.1 | *Agathobacter rectalis* | DFI.9.42 |
| NZ_JAJCJM010000019.1 | *Agathobacter rectalis* | DFI.9.37 |
| NZ_JAJCJP010000007.1 | *Agathobacter rectalis* | DFI.7.31 |
| NZ_JAJCJQ010000013.1 | *Agathobacter rectalis* | DFI.7.28A |
| NZ_JAJCJR010000014.1 | *Agathobacter rectalis* | DFI.7.23A |
| NZ_JAJFBX010000004.1 | *Agathobacter rectalis* | MSK.22.92 |
| NZ_JAKNFM010000017.1 | *Agathobacter rectalis* | DFI.5.3 |
| NZ_JANFYL010000009.1 | *Agathobacter rectalis* | SL.1.07 |
| NZ_JANFZO010000012.1 | *Agathobacter rectalis* | DFI.9.50 |
| NZ_JANFZZ010000012.1 | *Agathobacter rectalis* | DFI.9.12 |
| NZ_JANGBN010000012.1 | *Agathobacter rectalis* | DFI.6.27 |
| NZ_JAQDCR010000019.1 | *Agathobacter rectalis* | AF67-03PH9 |
| NZ_JAQESI010000016.1 | *Agathobacter rectalis* | AM97-21 |
| NZ_JAQLXY010000010.1 | *Agathobacter rectalis* | D54st1_G3_D54t1_190329 |
| NZ_JAQLXZ010000033.1 | *Agathobacter rectalis* | D54st1_D9_D54t1_190329 |
| NZ_JAQLYA010000012.1 | *Agathobacter rectalis* | D53st1_E10_D53t1_180928 |
| NZ_JAQLYB010000005.1 | *Agathobacter rectalis* | D53st1_B3_D53t1_180928 |
| NZ_JAQLYC010000021.1 | *Agathobacter rectalis* | 1001283st1_E6_1001283B150304_161114 |
| NZ_JAQLYD010000020.1 | *Agathobacter rectalis* | 1001283st1_D5_1001283B150225_161107 |
| NZ_JAQLYE010000021.1 | *Agathobacter rectalis* | 1001283st1_D2_1001283B150209_150212 |
| NZ_JAQLYF010000019.1 | *Agathobacter rectalis* | 1001283st1_B5_1001283B150210_160208 |
| NZ_JBBNFZ010000066.1 | *Agathobacter rectalis* | CLA-AA-H166 |
| NZ_JBBNIV010000025.1 | *Agathobacter rectalis* | CLA-AA-H258 |
| NZ_JBBNJQ010000026.1 | *Agathobacter rectalis* | CLA-AA-H285 |
| NZ_JBBNNI010000001.1 | *Agathobacter rectalis* | CLA-JM-H42 |
| NZ_JBCIUJ010000006.1 | *Agathobacter rectalis* | RTP31141st1_F9_RTP31141_220114 |
| NZ_JBCOUU010000017.1 | *Agathobacter rectalis* | RTP31023st1_F2_RTP31023_210422 |
| NZ_JBCPEY010000013.1 | *Agathobacter rectalis* | RTP21281st1_B9_RTP21281_210402 |
| NZ_JBDGCD010000011.1 | *Agathobacter rectalis* | RTP21359st1_A2_RTP21359_211015 |
| NZ_JBDGCE010000015.1 | *Agathobacter rectalis* | RTP21359st1_G5_RTP21360_211022 |
| NZ_JBDGCF010000004.1 | *Agathobacter rectalis* | RTP21360st1_B3_RTP21360_211022 |
| NZ_JBDMEJ010000011.1 | *Agathobacter rectalis* | RTP31015st1_H8_RTP31015_201113 |
| NZ_JBDMEK010000012.1 | *Agathobacter rectalis* | RTP31015st1_H6_RTP31015_201113 |
| NZ_JBDMEL010000001.1 | *Agathobacter rectalis* | RTP31015st1_G3_RTP31015_201113 |
| NZ_JBDMEM010000028.1 | *Agathobacter rectalis* | RTP31015st1_B6_RTP31015_201113 |
| NZ_JRFS01000015.1 | *Agathobacter rectalis* | R22 |
| NZ_QRIB01000003.1 | *Agathobacter rectalis* | AM22-4 |
| NZ_QRKN01000009.1 | *Agathobacter rectalis* | AM16-11 |
| NZ_QROF01000010.1 | *Agathobacter rectalis* | AF39-14AC |
| NZ_QRON01000002.1 | *Agathobacter rectalis* | AF38-24 |
| NZ_QRPB01000016.1 | *Agathobacter rectalis* | AF36-2BH |
| NZ_QRUJ01000013.1 | *Agathobacter rectalis* | AF25-15 |
| NZ_QRUL01000015.1 | *Agathobacter rectalis* | AF24-9LB |
| NZ_QRUX01000015.1 | *Agathobacter rectalis* | AF24-17 |
| NZ_QRWI01000009.1 | *Agathobacter rectalis* | AF19-4 |
| NZ_QRWK01000004.1 | *Agathobacter rectalis* | AF19-3AC |
| NZ_QRXF01000026.1 | *Agathobacter rectalis* | AF18-18LB |
| NZ_QRXG01000027.1 | *Agathobacter rectalis* | AF18-16LB |
| NZ_QRXR01000022.1 | *Agathobacter rectalis* | AF17-27 |
| NZ_QSAZ01000013.1 | *Agathobacter rectalis* | AF06-19 |
| NZ_QSEY01000034.1 | *Agathobacter rectalis* | AM44-2AT |
| NZ_QSFB01000002.1 | *Agathobacter rectalis* | AM44-1AT |
| NZ_QSFZ01000002.1 | *Agathobacter rectalis* | AM42-17AT |
| NZ_QSGF01000001.1 | *Agathobacter rectalis* | AM42-1 |
| NZ_QSHU01000010.1 | *Agathobacter rectalis* | AM36-3AA |
| NZ_QSJS01000001.1 | *Agathobacter rectalis* | AM30-13AC |
| NZ_QSKC01000005.1 | *Agathobacter rectalis* | AM29-10 |
| NZ_QSKY01000032.1 | *Agathobacter rectalis* | AM26-2LB |
| NZ_QSOB01000004.1 | *Agathobacter rectalis* | TM10-3 |
| NZ_QSQP01000008.1 | *Agathobacter rectalis* | TF11-15AC |
| NZ_QSTI01000015.1 | *Agathobacter rectalis* | OM08-12AT |
| NZ_QSTP01000018.1 | *Agathobacter rectalis* | OM07-13 |
| NZ_QSUE01000010.1 | *Agathobacter rectalis* | OM05-8AA |
| NZ_QSUG01000012.1 | *Agathobacter rectalis* | OM05-6AA |
| NZ_QSUH01000010.1 | *Agathobacter rectalis* | OM05-3AA |
| NZ_SPFX01000014.1 | *Agathobacter rectalis* | 1001271st1_F12 |
| NZ_VSTF01000014.1 | *Agathobacter rectalis* | T3WBe13 |
| NZ_VSTG01000014.1 | *Agathobacter rectalis* | L2-21 |
| NZ_WKQO01000003.1 | *Agathobacter rectalis* | BIOML-A12 |
| NZ_WKQP01000022.1 | *Agathobacter rectalis* | BIOML-A11 |
| NZ_WKQQ01000003.1 | *Agathobacter rectalis* | BIOML-A10 |
| NZ_WKQR01000014.1 | *Agathobacter rectalis* | BIOML-A9 |
| NZ_WKQS01000003.1 | *Agathobacter rectalis* | BIOML-A8 |
| NZ_WKQT01000012.1 | *Agathobacter rectalis* | BIOML-A7 |
| NZ_WKQU01000013.1 | *Agathobacter rectalis* | BIOML-A6 |
| NZ_WKQV01000003.1 | *Agathobacter rectalis* | BIOML-A5 |
| NZ_WKQW01000003.1 | *Agathobacter rectalis* | BIOML-A4 |
| NZ_WKQX01000003.1 | *Agathobacter rectalis* | BIOML-A3 |
| NZ_WKQY01000012.1 | *Agathobacter rectalis* | BIOML-A2 |
| NZ_WKQZ01000003.1 | *Agathobacter rectalis* | BIOML-A1 |
| NZ_WQOP01000039.1 | *Agathobacter rectalis* | MCC553 |
| NZ_WQOQ01000091.1 | *Agathobacter rectalis* | MCC552 |
| NC_012781.1 | *Agathobacter rectalis ATCC 33656* | ATCC 33656 |
| NC_021010.1 | *Agathobacter rectalis DSM 17629* | DSM 17629 |
| NC_021044.1 | *Agathobacter rectalis M104/1* | M104/1 |
| NZ_BMOY01000048.1 | *Alicyclobacillus cellulosilyticus* | JCM 18487 |
| NZ_KE386913.1 | *Alicyclobacillus contaminans DSM 17975* | DSM 17975 |
| NZ_LJCO01000014.1 | *Alicyclobacillus ferrooxydans* | TC-34 |
| NZ_AUMH01000005.1 | *Alicyclobacillus herbarius DSM 13609* | DSM 13609 |
| NZ_BCRP01000008.1 | *Alicyclobacillus kakegawensis NBRC 103104* | NBRC 103104 |
| NZ_FPBV01000002.1 | *Alicyclobacillus macrosporangiidus* | DSM 17980 |
| NZ_JNIL01000001.1 | *Alicyclobacillus macrosporangiidus CPP55* | CPP55 |
| NZ_BCQV01000023.1 | *Alicyclobacillus shizuokensis NBRC 103103* | NBRC 103103 |
| NZ_JAJFNK010000061.1 | *Alicyclobacillus tolerans* | BL-1 |
| NZ_CP058559.1 | *Alkalicella caledoniensis* | LB2 |
| NC_009633.1 | *Alkaliphilus metalliredigens QYMF* | QYMF |
| NZ_JAUORD010000012.1 | *Anaerobacillus sp. 1_MG-2023* | C3R10 |
| NZ_QPJT01000015.1 | *Anaerobacterium chartisolvens* | DSM 27016 |
| NZ_BAABFM010000048.1 | *Anaerocolumna aminovalerica* | JCM 1421 |
| NZ_FOWD01000014.1 | *Anaerocolumna aminovalerica* | DSM 1283 |
| NZ_JAHLON010000049.1 | *Anaerocolumna aminovalerica* | N98 |
| NZ_CP048000.1 | *Anaerocolumna sedimenticola* | CBA3638 |
| NZ_BAABRZ010000004.1 | *Anaerostipes caccae* | NBRC 114412 |
| NZ_CABIYM010000001.1 | *Anaerostipes caccae* |  |
| NZ_CP084016.1 | *Anaerostipes caccae* | NBRC 114412 |
| NZ_CP143937.1 | *Anaerostipes caccae* | DFI.7.76 |
| NZ_GL629687.1 | *Anaerostipes caccae* | 3_2_56FAA |
| NZ_JAJCLF010000004.1 | *Anaerostipes caccae* | DFI.1.56 |
| NZ_JAJCLH010000004.1 | *Anaerostipes caccae* | DFI.1.123 |
| NZ_JAJCLY010000006.1 | *Anaerostipes caccae* | SL.2.05 |
| NZ_JAJCPI010000003.1 | *Anaerostipes caccae* | DFI.1.222 |
| NZ_JAJCPJ010000004.1 | *Anaerostipes caccae* | DFI.1.221 |
| NZ_JAJCPM010000003.1 | *Anaerostipes caccae* | DFI.1.218 |
| NZ_JAJCPP010000003.1 | *Anaerostipes caccae* | DFI.1.214 |
| NZ_JAJCQH010000003.1 | *Anaerostipes caccae* | DFI.1.166 |
| NZ_JAJCQQ010000004.1 | *Anaerostipes caccae* | DFI.1.134 |
| NZ_JAJCRE010000002.1 | *Anaerostipes caccae* | DFI.1.94 |
| NZ_JAJCRG010000002.1 | *Anaerostipes caccae* | DFI.1.5 |
| NZ_JAJCRH010000002.1 | *Anaerostipes caccae* | DFI.1.4 |
| NZ_JAJCRI010000002.1 | *Anaerostipes caccae* | DFI.1.3 |
| NZ_JAJCRK010000002.1 | *Anaerostipes caccae* | DFI.1.19 |
| NZ_JAJCRL010000002.1 | *Anaerostipes caccae* | DFI.1.13 |
| NZ_JAKNED010000004.1 | *Anaerostipes caccae* | DFI.1.59 |
| NZ_JANGAQ010000003.1 | *Anaerostipes caccae* | DFI.7.76 |
| NZ_JAQFAK010000005.1 | *Anaerostipes caccae* | OM13-19BHA |
| NZ_JBBBVS010000002.1 | *Anaerostipes caccae* | CML199 |
| NZ_JBDKXL010000002.1 | *Anaerostipes caccae* | AGMB14476 |
| NZ_AP023027.1 | *Anaerostipes caccae L1-92* | JCM 13470 |
| NZ_CP036345.1 | *Anaerostipes caccae L1-92* | DSM 14662 |
| NZ_CP102254.1 | *Anaerostipes caccae L1-92* | DSM 14662 |
| NZ_DS499730.1 | *Anaerostipes caccae L1-92* | DSM 14662 |
| NZ_UYXC01000024.1 | *Anaerostipes caccae L1-92* |  |
| NZ_CAJSYK010000001.1 | *Anaerostipes faecis* |  |
| NZ_JAQEHL010000011.1 | *Anaerostipes hominis (ex Lee et al. 2021)* | AM75-12pH10A |
| NZ_JAQEVE010000012.1 | *Anaerostipes hominis (ex Lee et al. 2021)* | OF05-1 |
| NZ_JBCLTR010000003.1 | *Anaerostipes hominis (ex Lee et al. 2021)* | 32-10 |
| NZ_NQOG01000002.1 | *Anaerostipes hominis (ex Lee et al. 2021)* | BG01 |
| NZ_JACOOS010000001.1 | *Anaerostipes hominis (ex Liu et al. 2021)* | NSJ-7 |
| NZ_CP040058.1 | *Anaerostipes rhamnosivorans* | 1y2 |
| NZ_QTXQ01000009.1 | *Anaerostipes sp. AF04-45* | AF04-45 |
| NZ_JANFXK010000004.1 | *Anaerovorax odorimutans* | SL.3.17 |
| NZ_JAQOTG010000027.1 | *Anoxybacillus rupiensis* | DSM 17127 |
| NZ_CP064048.1 | *Anoxybacillus sediminis* | PCH 117 |
| NZ_CP047158.1 | *Anoxybacillus sp. PDR2* | PDR2 |
| NZ_JAHQCR010000070.1 | *Bacillus alkalicola* | JCM 17098 |
| NZ_JAPJMC010000007.1 | *Bacillus cereus* | BY124LC |
| NZ_JAPJMJ010000013.1 | *Bacillus cereus* | BY106LC |
| NZ_JAZIBI010000006.1 | *Bacillus cereus* | B10 |
| NZ_JAZIEZ010000006.1 | *Bacillus cereus* | B3 |
| NZ_JAZIFW010000006.1 | *Bacillus cereus* | B11 |
| NZ_LZQY01000038.1 | *Bacillus cereus* | A8S3 |
| NZ_MIFP01000035.1 | *Bacillus cereus* | MOD1_Bc77 |
| NZ_MPOL01000007.1 | *Bacillus cereus* | NZAS01 |
| NZ_NUMG01000016.1 | *Bacillus cereus* | AFS040105 |
| NZ_NUOJ01000012.1 | *Bacillus cereus* | AFS033081 |
| NZ_NUPL01000001.1 | *Bacillus cereus* | AFS030229 |
| NZ_QHHK01000001.1 | *Bacillus cereus* | BS16 |
| NZ_JAQFPI010000016.1 | *Bacillus cereus group sp. BcHK130* | BcHK130 |
| NZ_JAQFPF010000006.1 | *Bacillus cereus group sp. BcHK140* | BcHK140 |
| NZ_JAKUFE010000004.1 | *Bacillus cereus group sp. BfR-BA-01409* | BfR-BA-01409 |
| NZ_JAQFOU010000022.1 | *Bacillus cereus group sp. BY128LC* | BY128LC |
| NZ_JAQFOJ010000003.1 | *Bacillus cereus group sp. BY5-1LC* | BY5-1LC |
| NZ_JAQFNX010000015.1 | *Bacillus cereus group sp. m1-1* | m1-1 |
| NZ_JAQFNW010000014.1 | *Bacillus cereus group sp. m1-11* | m1-11 |
| NZ_JAQFNV010000014.1 | *Bacillus cereus group sp. m1-16* | m1-16 |
| NZ_JAQFNU010000014.1 | *Bacillus cereus group sp. m1-2* | m1-2 |
| NZ_JAQFNT010000014.1 | *Bacillus cereus group sp. m1-21* | m1-21 |
| NZ_JAQFNS010000014.1 | *Bacillus cereus group sp. m1-22* | m1-22 |
| NZ_JAQFNR010000013.1 | *Bacillus cereus group sp. m1-4* | m1-4 |
| NZ_JAQFNQ010000015.1 | *Bacillus cereus group sp. m2-12* | m2-12 |
| NZ_JAQFNP010000014.1 | *Bacillus cereus group sp. m2-21* | m2-21 |
| NZ_JAQFNO010000014.1 | *Bacillus cereus group sp. m2-68* | m2-68 |
| NZ_JARVHD010000001.1 | *Bacillus cereus group sp. MG11* | MG11 |
| NZ_JAQFMG010000013.1 | *Bacillus cereus group sp. TH41-1LC* | TH41-1LC |
| NZ_JAQFMF010000027.1 | *Bacillus cereus group sp. TH43LC* | TH43LC |
| NZ_JOTN01000009.1 | *Bacillus manliponensis* | JCM 15802 |
| NZ_JARMAI010000038.1 | *Bacillus mycoides* | B-23424 |
| NZ_JALAXF010000030.1 | *Bacillus paranthracis* | T2B1 |
| NZ_JAPNPJ010000017.1 | *Bacillus paranthracis* | 64.1 |
| NZ_JAPNPP010000018.1 | *Bacillus paranthracis* | 59.1 |
| NZ_JAPNPQ010000016.1 | *Bacillus paranthracis* | 55.2 |
| NZ_JAPNQH010000035.1 | *Bacillus paranthracis* | 19.1 |
| NZ_JARMCL010000088.1 | *Bacillus paranthracis* | B-4266 |
| NZ_JARSZF010000031.1 | *Bacillus paranthracis* | HD-690 |
| NZ_JAZDXB010000015.1 | *Bacillus paranthracis* | cyp-38L |
| NZ_LEBY01000070.1 | *Bacillus paranthracis* | AH827 |
| NZ_NJQK01000004.1 | *Bacillus paranthracis* | 18-1 |
| NZ_VKPH01000011.1 | *Bacillus paranthracis* | ATCC 10702 |
| NZ_VLYQ01000409.1 | *Bacillus paranthracis* | NR-608 |
| NZ_VEAS01000008.1 | *Bacillus pumilus SAFR-032* | DE0317 |
| NZ_JAMAUL010000006.1 | *Bacillus safensis* | MER 10 |
| NZ_JAWJLN010000002.1 | *Bacillus safensis* | Ni7 |
| NZ_JXCO01000014.1 | *Bacillus safensis* | B4134 |
| NZ_WEHU01000004.1 | *Bacillus sp. B1-b2* | B1-b2 |
| NZ_QOZX01000048.1 | *Bacillus sp. BB081* | BB081 |
| NZ_FOYF01000003.1 | *Bacillus sp. cl95* | CL95 |
| NZ_MAQX01000032.1 | *Bacillus sp. FJAT-26390* | FJAT-26390 |
| NZ_LIGI01000001.1 | *Bacillus sp. FJAT-27245* | FJAT-27245 |
| NZ_CP151985.1 | *Bacillus sp. FSL M8-0350* | FSL M8-0350 |
| NZ_FOJZ01000002.1 | *Bacillus sp. UNCCL13* | UNCCL13 |
| NZ_JARAFZ010000007.1 | *Bacillus thuringiensis* | FC2 |
| NZ_JYCL01000025.1 | *Bacillus thuringiensis* | Lr7/2 |
| NZ_VEFL01000022.1 | *Bacillus thuringiensis* | DE0141 |
| NZ_VEIE01000003.1 | *Bacillus thuringiensis* | DE0031 |
| NZ_VEMT01000007.1 | *Bacillus tropicus* | DE0016 |
| NZ_CP036073.1 | *Bacillus wiedmannii* | JAS08/1 |
| NZ_CP126099.1 | *Bacillus wiedmannii* | LN15 |
| NZ_NUET01000046.1 | *Bacillus wiedmannii* | AFS002508 |
| NZ_NURG01000026.1 | *Bacillus wiedmannii* | AFS053236 |
| NZ_NUTO01000072.1 | *Bacillus wiedmannii* | AFS036771 |
| NZ_NVJH01000037.1 | *Bacillus wiedmannii* | AFS063338 |
| NZ_NVJV01000024.1 | *Bacillus wiedmannii* | AFS061181 |
| NZ_JAJCIQ010000002.1 | *Bariatricus massiliensis* | DFI.1.181 |
| NZ_JAJCIR010000002.1 | *Bariatricus massiliensis* | DFI.1.179 |
| NZ_JAJCIS010000002.1 | *Bariatricus massiliensis* | DFI.1.165 |
| NZ_JAJCQI010000001.1 | *Bariatricus massiliensis* | DFI.1.164 |
| NZ_JANGDQ010000001.1 | *Bariatricus massiliensis* | DFI.1.82 |
| NZ_LT574838.1 | *Bariatricus massiliensis* | AT12 |
| NZ_CABKUA010000004.1 | *Beduini massiliensis* |  |
| NZ_CDPP01000004.1 | *Beduini massiliensis* | GM1 |
| NZ_JANJZU010000012.1 | *Blautia coccoides* | DSM 29138 |
| NZ_JBCITU010000005.1 | *Blautia coccoides* | RTP31141st1_E4_RTP31141_220114 |
| NZ_JBCITV010000005.1 | *Blautia coccoides* | RTP31141st1_E7_RTP31141_220114 |
| NZ_NQOF01000001.1 | *Blautia hominis* | KB1 |
| NZ_CABMKU010000001.1 | *Blautia marasmi* |  |
| NZ_JBCLQG010000002.1 | *Blautia marasmi* | 55-64 |
| NZ_OIWX01000001.1 | *Blautia marasmi* | Marseille-P2377 |
| NZ_CABLBP010000019.1 | *Blautia producta* |  |
| NZ_CP035945.1 | *Blautia producta* | PMF1 |
| NZ_CP039126.1 | *Blautia producta* | DSM 2950 |
| NZ_JANFXW010000014.1 | *Blautia producta* | SL.2.22 |
| NZ_AUUC01000004.1 | *Blautia producta ATCC 27340 = DSM 2950* | DSM 2950 |
| NZ_CP048626.1 | *Blautia producta ATCC 27340 = DSM 2950* | JCM 1471 |
| NZ_KB892647.1 | *Blautia producta ATCC 27340 = DSM 2950* | ATCC 27340 |
| NZ_SZNK01000001.1 | *Brevibacillus antibioticus* | TGS2-1 |
| NZ_JAUSVZ010000012.1 | *Brevibacillus aydinogluensis* | PDF25 |
| NZ_CABMGL010000086.1 | *Brevibacillus massiliensis* |  |
| NZ_HE978549.1 | *Brevibacillus massiliensis* | phR |
| NZ_KI629782.1 | *Brevibacillus panacihumi W25* | W25 |
| NZ_JAQFIQ010000046.1 | *Breznakia sp. PF5-3* | PF5-3 |
| NZ_JAQFIR010000057.1 | *Breznakia sp. PFB2-8* | PFB2-8 |
| NZ_JAQFIS010000044.1 | *Breznakia sp. PH5-24* | PH5-24 |
| NZ_JAQFIO010000044.1 | *Breznakia sp. PM6-1* | PM6-1 |
| NZ_ATVZ01000066.1 | *Butyrivibrio fibrisolvens AB2020* | AB2020 |
| NZ_JANSWL010000042.1 | *Butyrivibrio sp. DSM 10294* | DSM 10294 |
| NZ_FRCR01000021.1 | *Caldanaerovirga acetigignens* | DSM 18802 |
| NZ_BDGJ01000117.1 | *Calderihabitans maritimus* | KKC1 |
| NZ_JAHUQD010000002.1 | *Caldicoprobacter algeriensis* | TH7C1 |
| NZ_FOXR01000025.1 | *Caldicoprobacter faecalis* | DSM 20678 |
| NZ_JAFBDW010000007.1 | *Caldicoprobacter guelmensis* | DSM 24605 |
| NZ_VTPS01000025.1 | *Calorimonas adulescens* | A05MB |
| NZ_CP060286.1 | *Caproicibacter fermentans* | 7D4C2 |
| NZ_BDLR01000076.1 | *Carboxydocella sp. JDF658* | JDF658 |
| NZ_BDLQ01000033.1 | *Carboxydocella sp. ULO1* | ULO1 |
| NZ_FUXM01000003.1 | *Carboxydocella sporoproducens DSM 16521* | DSM 16521 |
| NZ_CP028491.1 | *Carboxydocella thermautotrophica* | 019 |
| NZ_ATYG01000019.1 | *Carboxydothermus ferrireducens DSM 11255* | DSM 11255 |
| NZ_JACCBS010000003.1 | *Carboxydothermus ferrireducens DSM 11255* | DSM 11255 |
| NC_007503.1 | *Carboxydothermus hydrogenoformans Z-2901* | Z-2901 |
| NZ_BDJL01000019.1 | *Carboxydothermus islandicus* | SET IS-9 |
| NZ_BDJK01000055.1 | *Carboxydothermus pertinax* | Ug1 |
| NZ_GL892076.1 | *Centipeda periodontii DSM 2778* | DSM 2778 |
| NZ_CP151997.1 | *Chryseomicrobium sp. FSL W7-1435* | FSL W7-1435 |
| NZ_JADCKL010000008.1 | *Claveliimonas monacensis* | DSM 108991 |
| NZ_BKAK01000012.1 | *Clostridium beijerinckii* | NBRC 109359 |
| NZ_CP016090.1 | *Clostridium beijerinckii* | BAS/B3/I/124 |
| NZ_CP073279.1 | *Clostridium beijerinckii* | CBEI |
| NZ_CP144906.1 | *Clostridium beijerinckii* | BRM001 |
| NZ_JABAEA010000006.1 | *Clostridium beijerinckii* | DJ008 |
| NZ_JABAEL010000002.1 | *Clostridium beijerinckii* | DJ012 |
| NZ_JABAEM010000005.1 | *Clostridium beijerinckii* | DJ010 |
| NZ_JABAGV010000002.1 | *Clostridium beijerinckii* | DJ015 |
| NZ_JABAGW010000035.1 | *Clostridium beijerinckii* | DJ083 |
| NZ_JABAHJ010000003.1 | *Clostridium beijerinckii* | DJ157 |
| NZ_JABAJC010000022.1 | *Clostridium beijerinckii* | DJ222 |
| NZ_JABAJD010000062.1 | *Clostridium beijerinckii* | DJ231 |
| NZ_JABAJE010000007.1 | *Clostridium beijerinckii* | DJ245 |
| NZ_JABAJG010000003.1 | *Clostridium beijerinckii* | DJ266 |
| NZ_JABAJH010000005.1 | *Clostridium beijerinckii* | DJ270 |
| NZ_JABANS010000060.1 | *Clostridium beijerinckii* | DJ232 |
| NZ_JABANV010000011.1 | *Clostridium beijerinckii* | DJ275 |
| NZ_JABANW010000060.1 | *Clostridium beijerinckii* | DJ287 |
| NZ_JABFUL010000003.1 | *Clostridium beijerinckii* | DJ076 |
| NZ_JABFUM010000001.1 | *Clostridium beijerinckii* | DJ077 |
| NZ_JABFUN010000001.1 | *Clostridium beijerinckii* | DJ078 |
| NZ_JABFUP010000002.1 | *Clostridium beijerinckii* | DJ091 |
| NZ_JABSWA010000001.1 | *Clostridium beijerinckii* | DJ019 |
| NZ_JABSWB010000001.1 | *Clostridium beijerinckii* | DJ027 |
| NZ_JABSWE010000001.1 | *Clostridium beijerinckii* | DJ022 |
| NZ_JABSWF010000001.1 | *Clostridium beijerinckii* | DJ044 |
| NZ_JABSWG010000001.1 | *Clostridium beijerinckii* | DJ045 |
| NZ_JABSWH010000001.1 | *Clostridium beijerinckii* | DJ047 |
| NZ_JABSWI010000001.1 | *Clostridium beijerinckii* | DJ032 |
| NZ_JABSWO010000001.1 | *Clostridium beijerinckii* | DJ001 |
| NZ_JABSWP010000001.1 | *Clostridium beijerinckii* | DJ020 |
| NZ_JABSWQ010000001.1 | *Clostridium beijerinckii* | DJ026 |
| NZ_JABSWR010000001.1 | *Clostridium beijerinckii* | DJ040 |
| NZ_JABSWS010000003.1 | *Clostridium beijerinckii* | DJ052 |
| NZ_JABSWT010000001.1 | *Clostridium beijerinckii* | DJ057 |
| NZ_JABSWU010000001.1 | *Clostridium beijerinckii* | DJ058 |
| NZ_JABSWV010000001.1 | *Clostridium beijerinckii* | DJ073 |
| NZ_JABSWX010000001.1 | *Clostridium beijerinckii* | DJ084 |
| NZ_JABSWY010000001.1 | *Clostridium beijerinckii* | DJ089 |
| NZ_JABSWZ010000001.1 | *Clostridium beijerinckii* | DJ097 |
| NZ_JABSXA010000001.1 | *Clostridium beijerinckii* | DJ102 |
| NZ_JABSXB010000001.1 | *Clostridium beijerinckii* | DJ105 |
| NZ_JABSXC010000001.1 | *Clostridium beijerinckii* | DJ106 |
| NZ_JABSXD010000003.1 | *Clostridium beijerinckii* | DJ107 |
| NZ_JABSXE010000001.1 | *Clostridium beijerinckii* | DJ110 |
| NZ_JABSXF010000001.1 | *Clostridium beijerinckii* | DJ112 |
| NZ_JABSXG010000001.1 | *Clostridium beijerinckii* | DJ116 |
| NZ_JABSXH010000001.1 | *Clostridium beijerinckii* | DJ119 |
| NZ_JABSXI010000001.1 | *Clostridium beijerinckii* | DJ121 |
| NZ_JABSXJ010000001.1 | *Clostridium beijerinckii* | DJ125 |
| NZ_JABSXK010000001.1 | *Clostridium beijerinckii* | DJ126 |
| NZ_JABSXL010000001.1 | *Clostridium beijerinckii* | DJ127 |
| NZ_JABSXM010000001.1 | *Clostridium beijerinckii* | DJ128 |
| NZ_JABSXN010000001.1 | *Clostridium beijerinckii* | DJ154 |
| NZ_JABSXO010000001.1 | *Clostridium beijerinckii* | DJ155 |
| NZ_JABSXP010000001.1 | *Clostridium beijerinckii* | DJ158 |
| NZ_JABSXQ010000001.1 | *Clostridium beijerinckii* | DJ159 |
| NZ_JABSXR010000001.1 | *Clostridium beijerinckii* | DJ180 |
| NZ_JABSXS010000001.1 | *Clostridium beijerinckii* | DJ182 |
| NZ_JABSXT010000001.1 | *Clostridium beijerinckii* | DJ184 |
| NZ_JABSXU010000002.1 | *Clostridium beijerinckii* | DJ185 |
| NZ_JABSXV010000001.1 | *Clostridium beijerinckii* | DJ187 |
| NZ_JABSXW010000001.1 | *Clostridium beijerinckii* | DJ226 |
| NZ_JABSXX010000001.1 | *Clostridium beijerinckii* | DJ227 |
| NZ_JABSXY010000001.1 | *Clostridium beijerinckii* | DJ228.2 |
| NZ_JABSXZ010000001.1 | *Clostridium beijerinckii* | DJ229 |
| NZ_JABSYA010000001.1 | *Clostridium beijerinckii* | DJ233 |
| NZ_JABSYB010000003.1 | *Clostridium beijerinckii* | DJ234 |
| NZ_JABSYC010000001.1 | *Clostridium beijerinckii* | DJ236 |
| NZ_JABSYD010000001.1 | *Clostridium beijerinckii* | DJ237 |
| NZ_JABSYE010000001.1 | *Clostridium beijerinckii* | DJ238 |
| NZ_JABSYF010000001.1 | *Clostridium beijerinckii* | DJ239 |
| NZ_JABSYG010000001.1 | *Clostridium beijerinckii* | DJ241 |
| NZ_JABSYH010000001.1 | *Clostridium beijerinckii* | DJ243 |
| NZ_JABSYI010000001.1 | *Clostridium beijerinckii* | DJ244 |
| NZ_JABSYJ010000001.1 | *Clostridium beijerinckii* | DJ248 |
| NZ_JABSYK010000001.1 | *Clostridium beijerinckii* | DJ251 |
| NZ_JABSYL010000001.1 | *Clostridium beijerinckii* | DJ253 |
| NZ_JABSYM010000001.1 | *Clostridium beijerinckii* | DJ254 |
| NZ_JABSYN010000001.1 | *Clostridium beijerinckii* | DJ255 |
| NZ_JABSYO010000001.1 | *Clostridium beijerinckii* | DJ259 |
| NZ_JABSYP010000001.1 | *Clostridium beijerinckii* | DJ261 |
| NZ_JABSYQ010000001.1 | *Clostridium beijerinckii* | DJ264 |
| NZ_JABSYR010000001.1 | *Clostridium beijerinckii* | DJ265 |
| NZ_JABSYS010000001.1 | *Clostridium beijerinckii* | DJ269 |
| NZ_JABSYT010000001.1 | *Clostridium beijerinckii* | DJ272 |
| NZ_JABSYU010000001.1 | *Clostridium beijerinckii* | DJ273 |
| NZ_JABSYV010000001.1 | *Clostridium beijerinckii* | DJ274 |
| NZ_JABSYW010000001.1 | *Clostridium beijerinckii* | DJ085 |
| NZ_JABSYX010000001.1 | *Clostridium beijerinckii* | DJ141 |
| NZ_JABSYY010000001.1 | *Clostridium beijerinckii* | DJ161 |
| NZ_JABSYZ010000001.1 | *Clostridium beijerinckii* | DJ164 |
| NZ_JABSZA010000001.1 | *Clostridium beijerinckii* | DJ165 |
| NZ_JABSZB010000001.1 | *Clostridium beijerinckii* | DJ166 |
| NZ_JABSZC010000001.1 | *Clostridium beijerinckii* | DJ170 |
| NZ_JABSZD010000001.1 | *Clostridium beijerinckii* | DJ171 |
| NZ_JABSZE010000001.1 | *Clostridium beijerinckii* | DJ173 |
| NZ_JABSZF010000001.1 | *Clostridium beijerinckii* | DJ174 |
| NZ_JABSZG010000001.1 | *Clostridium beijerinckii* | DJ175 |
| NZ_JABSZH010000003.1 | *Clostridium beijerinckii* | DJ176 |
| NZ_JABSZI010000001.1 | *Clostridium beijerinckii* | DJ195 |
| NZ_JABSZJ010000001.1 | *Clostridium beijerinckii* | DJ196 |
| NZ_JABSZK010000001.1 | *Clostridium beijerinckii* | DJ198 |
| NZ_JABSZL010000001.1 | *Clostridium beijerinckii* | DJ200 |
| NZ_JABSZM010000001.1 | *Clostridium beijerinckii* | DJ203 |
| NZ_JABSZN010000001.1 | *Clostridium beijerinckii* | DJ204 |
| NZ_JABSZO010000001.1 | *Clostridium beijerinckii* | DJ206 |
| NZ_JABSZP010000001.1 | *Clostridium beijerinckii* | DJ208 |
| NZ_JABSZQ010000001.1 | *Clostridium beijerinckii* | DJ212 |
| NZ_JABSZR010000001.1 | *Clostridium beijerinckii* | DJ213 |
| NZ_JABSZS010000001.1 | *Clostridium beijerinckii* | DJ215 |
| NZ_JABSZT010000001.1 | *Clostridium beijerinckii* | DJ216 |
| NZ_JABSZU010000001.1 | *Clostridium beijerinckii* | DJ217 |
| NZ_JABSZV010000002.1 | *Clostridium beijerinckii* | DJ221 |
| NZ_JABSZW010000002.1 | *Clostridium beijerinckii* | DJ223 |
| NZ_JABSZX010000001.1 | *Clostridium beijerinckii* | DJ277 |
| NZ_JABSZY010000002.1 | *Clostridium beijerinckii* | DJ278 |
| NZ_JABSZZ010000001.1 | *Clostridium beijerinckii* | DJ279 |
| NZ_JABTAA010000001.1 | *Clostridium beijerinckii* | DJ283 |
| NZ_JABTAB010000001.1 | *Clostridium beijerinckii* | DJ289 |
| NZ_JABTAC010000001.1 | *Clostridium beijerinckii* | DJ290 |
| NZ_JABTAE010000001.1 | *Clostridium beijerinckii* | DJ316 |
| NZ_JABTAF010000001.1 | *Clostridium beijerinckii* | DJ317 |
| NZ_JABTAH010000001.1 | *Clostridium beijerinckii* | DJ331 |
| NZ_JABTAI010000001.1 | *Clostridium beijerinckii* | DJ336 |
| NZ_JABTAJ010000001.1 | *Clostridium beijerinckii* | DJ092 |
| NZ_JABTAK010000002.1 | *Clostridium beijerinckii* | DJ131 |
| NZ_JABTAM010000001.1 | *Clostridium beijerinckii* | DJ139 |
| NZ_JABTAN010000001.1 | *Clostridium beijerinckii* | DJ140 |
| NZ_JABTAO010000001.1 | *Clostridium beijerinckii* | DJ143 |
| NZ_JABTAP010000001.1 | *Clostridium beijerinckii* | DJ144 |
| NZ_JABTAQ010000001.1 | *Clostridium beijerinckii* | DJ145 |
| NZ_JABTAR010000001.1 | *Clostridium beijerinckii* | DJ151 |
| NZ_JABTAS010000001.1 | *Clostridium beijerinckii* | DJ183 |
| NZ_JABTAU010000001.1 | *Clostridium beijerinckii* | DJ257 |
| NZ_JABTAV010000001.1 | *Clostridium beijerinckii* | DJ059 |
| NZ_JABTAW010000001.1 | *Clostridium beijerinckii* | DJ060 |
| NZ_JABTAY010000001.1 | *Clostridium beijerinckii* | DJ082 |
| NZ_JABTAZ010000003.1 | *Clostridium beijerinckii* | DJ149 |
| NZ_JABTBA010000001.1 | *Clostridium beijerinckii* | DJ153 |
| NZ_JABTBB010000001.1 | *Clostridium beijerinckii* | DJ163 |
| NZ_JABTBC010000003.1 | *Clostridium beijerinckii* | DJ168 |
| NZ_JABTBD010000001.1 | *Clostridium beijerinckii* | DJ177 |
| NZ_JABTBE010000001.1 | *Clostridium beijerinckii* | DJ202 |
| NZ_JABTBF010000001.1 | *Clostridium beijerinckii* | DJ214 |
| NZ_JABTBG010000001.1 | *Clostridium beijerinckii* | DJ258 |
| NZ_JABTBH010000001.1 | *Clostridium beijerinckii* | DJ199 |
| NZ_JABTBI010000001.1 | *Clostridium beijerinckii* | DJ050 |
| NZ_JABTBL010000001.1 | *Clostridium beijerinckii* | DJ054 |
| NZ_JABTBN010000001.1 | *Clostridium beijerinckii* | DJ225 |
| NZ_JABTDU010000001.1 | *Clostridium beijerinckii* | DJ211 |
| NZ_JABTDY010000002.1 | *Clostridium beijerinckii* | DJ009 |
| NZ_JABTDZ010000001.1 | *Clostridium beijerinckii* | DJ043 |
| NZ_JABTEC010000001.1 | *Clostridium beijerinckii* | DJ066 |
| NZ_JABTED010000001.1 | *Clostridium beijerinckii* | DJ095 |
| NZ_JABTEN010000001.1 | *Clostridium beijerinckii* | DJ181 |
| NZ_JABTEO010000005.1 | *Clostridium beijerinckii* | DJ186 |
| NZ_JABTEP010000001.1 | *Clostridium beijerinckii* | DJ235 |
| NZ_JABTEQ010000001.1 | *Clostridium beijerinckii* | DJ256 |
| NZ_JABTER010000002.1 | *Clostridium beijerinckii* | DJ129 |
| NZ_JABTES010000003.1 | *Clostridium beijerinckii* | DJ193 |
| NZ_JACAZL010000001.1 | *Clostridium beijerinckii* | DJ055 |
| NZ_JACAZM010000001.1 | *Clostridium beijerinckii* | DJ017 |
| NZ_JACAZN010000003.1 | *Clostridium beijerinckii* | DJ068 |
| NZ_JACAZO010000001.1 | *Clostridium beijerinckii* | DJ079 |
| NZ_JACAZQ010000001.1 | *Clostridium beijerinckii* | DJ108 |
| NZ_JACAZS010000001.1 | *Clostridium beijerinckii* | DJ230 |
| NZ_JACAZT010000002.1 | *Clostridium beijerinckii* | DJ240 |
| NZ_JACAZU010000001.1 | *Clostridium beijerinckii* | DJ162 |
| NZ_JACAZV010000001.1 | *Clostridium beijerinckii* | DJ197 |
| NZ_JACAZW010000008.1 | *Clostridium beijerinckii* | DJ201 |
| NZ_JACAZX010000001.1 | *Clostridium beijerinckii* | DJ207 |
| NZ_JACAZY010000001.1 | *Clostridium beijerinckii* | DJ219 |
| NZ_JACAZZ010000001.1 | *Clostridium beijerinckii* | DJ263 |
| NZ_JACBAA010000001.1 | *Clostridium beijerinckii* | DJ280 |
| NZ_JACBAC010000004.1 | *Clostridium beijerinckii* | DJ321 |
| NZ_JACBNM010000006.1 | *Clostridium beijerinckii* | DJ088 |
| NZ_JACBNO010000004.1 | *Clostridium beijerinckii* | DJ152 |
| NZ_JACERA010000006.1 | *Clostridium beijerinckii* | DJ218 |
| NZ_JACERB010000005.1 | *Clostridium beijerinckii* | DJ194 |
| NZ_JACERC010000005.1 | *Clostridium beijerinckii* | DJ209 |
| NZ_JACGYZ010000001.1 | *Clostridium beijerinckii* | DJ056 |
| NZ_JADOEF010000001.1 | *Clostridium beijerinckii* | NT08 |
| NZ_JAEVFY010000030.1 | *Clostridium beijerinckii* | G3_1a |
| NZ_JAEVFZ010000026.1 | *Clostridium beijerinckii* | G3_3b |
| NZ_JAFEKE010000030.1 | *Clostridium beijerinckii* | G3_5a |
| NZ_JAJSOL010000032.1 | *Clostridium beijerinckii* | CUEA02 |
| NZ_JAQCRM010000077.1 | *Clostridium beijerinckii* | AF101-19 |
| NZ_JARUNZ010000005.1 | *Clostridium beijerinckii* | csp1.3 |
| NZ_LZZD01000080.1 | *Clostridium beijerinckii* | BAS/B2 |
| NZ_LZZE01000079.1 | *Clostridium beijerinckii* | NCP 260 |
| NZ_LZZG01000020.1 | *Clostridium beijerinckii* | 4J9 |
| NZ_LZZH01000009.1 | *Clostridium beijerinckii* | NRRL B-591 |
| NZ_LZZI01000001.1 | *Clostridium beijerinckii* | DSM 53 |
| NZ_MBAF01000096.1 | *Clostridium beijerinckii* | BGS1 |
| NZ_UARH01000002.1 | *Clostridium beijerinckii* | NCTC13035 |
| NZ_CP006777.1 | *Clostridium beijerinckii ATCC 35702* | SA-1 |
| NC_009617.1 | *Clostridium beijerinckii NCIMB 8052* | NCIMB 8052 |
| NZ_CP011966.3 | *Clostridium beijerinckii NRRL B-598* | NRRL B-598 |
| NC_014393.1 | *Clostridium cellulovorans 743B* | 743B |
| NZ_WSRQ01000065.1 | *Clostridium chromiireducens* | NM79_F5 |
| NZ_JAAMNH020000001.1 | *Clostridium estertheticum* | FP3 |
| NZ_JAHLDR010000022.1 | *Clostridium estertheticum* | CF013 |
| NZ_AP024849.1 | *Clostridium gelidum* | C5S11 |
| NZ_PQCR01000067.1 | *Clostridium sp. 2-1* | 2-1 |
| NZ_QUFI01000001.1 | *Clostridium sp. AF27-2AA* | AF27-2AA |
| NZ_QUEH01000007.1 | *Clostridium sp. AF32-12BH* | AF32-12BH |
| NZ_QUIV01000009.1 | *Clostridium sp. AM33-3* | AM33-3 |
| NZ_QUIK01000003.1 | *Clostridium sp. AM42-4* | AM42-4 |
| NZ_JBFDTP010000001.1 | *Clostridium sp. AN503* | AN503 |
| NZ_SPGT01000023.1 | *Clostridium sp. BSD2780061688st1 H5* | BSD2780061688st1_H5 |
| NZ_GL870816.1 | *Clostridium sp. D5* | D5 |
| NZ_CM001240.1 | *Clostridium sp. DL-VIII* | DL-VIII |
| NZ_KE992738.1 | *Clostridium sp. KLE 1755* | KLE 1755 |
| NZ_WQPN01000022.1 | *Clostridium sp. MCC353* | MCC353 |
| NZ_BBDR01000015.1 | *Clostridium sp. NkU-1* | JCM 10519 |
| NZ_QUMD01000002.1 | *Clostridium sp. OF09-36* | OF09-36 |
| NZ_QULW01000010.1 | *Clostridium sp. OM02-18AC* | OM02-18AC |
| NZ_JAKGTG010000010.1 | *Clostridium sp. YIM B02506* | YIM B02506 |
| NZ_JAPPRQ010000043.1 | *Clostridium sp. ZS2-4* | ZS2-4 |
| NZ_CP021850.1 | *Clostridium thermosuccinogenes* | DSM 5807 |
| NZ_JBAIZC010000022.1 | *Clostridium thermosuccinogenes* | 1305-IL3214 |
| NZ_NIOI01000003.1 | *Clostridium thermosuccinogenes* | DSM 5809 |
| NZ_NIOJ01000087.1 | *Clostridium thermosuccinogenes* | DSM 5806 |
| NZ_NIOK01000089.1 | *Clostridium thermosuccinogenes* | DSM 5808 |
| NZ_UYZY01000003.1 | *Clostridium transplantifaecale* | Marseille-P8228 |
| NZ_QRDZ01000001.1 | *Cohnella phaseoli* | CECT 7287 |
| NZ_BSCI01000015.1 | *Coprococcus comes* | 31264 |
| NZ_CP070062.1 | *Coprococcus comes* | FDAARGOS_1339 |
| NZ_CYXR01000024.1 | *Coprococcus comes* | 2789STDY5834962 |
| NZ_CYYN01000016.1 | *Coprococcus comes* | 2789STDY5608832 |
| NZ_CYZK01000015.1 | *Coprococcus comes* | 2789STDY5834866 |
| NZ_JAAILK010000019.1 | *Coprococcus comes* | MSK.17.80 |
| NZ_JAAILT010000007.1 | *Coprococcus comes* | MSK.18.12 |
| NZ_JAAILU010000021.1 | *Coprococcus comes* | MSK.17.85 |
| NZ_JAAIMB010000023.1 | *Coprococcus comes* | MSK.17.59 |
| NZ_JAAIMF010000022.1 | *Coprococcus comes* | MSK.17.39 |
| NZ_JAAIMH010000022.1 | *Coprococcus comes* | MSK.17.27 |
| NZ_JAAIMI010000020.1 | *Coprococcus comes* | MSK.17.21 |
| NZ_JAAIMM010000001.1 | *Coprococcus comes* | MSK.16.63 |
| NZ_JAAIMN010000004.1 | *Coprococcus comes* | MSK.16.59 |
| NZ_JAAIMO010000004.1 | *Coprococcus comes* | MSK.16.50 |
| NZ_JAAIMV010000009.1 | *Coprococcus comes* | MSK.20.88 |
| NZ_JAAINA010000003.1 | *Coprococcus comes* | MSK.20.24 |
| NZ_JAAINK010000036.1 | *Coprococcus comes* | MSK.18.76 |
| NZ_JAAIOJ010000004.1 | *Coprococcus comes* | MSK.16.8 |
| NZ_JAAIOK010000004.1 | *Coprococcus comes* | MSK.16.14 |
| NZ_JAAIOL010000003.1 | *Coprococcus comes* | MSK.16.12 |
| NZ_JAAIOM010000005.1 | *Coprococcus comes* | MSK.16.11 |
| NZ_JAAION010000027.1 | *Coprococcus comes* | MSK.11.53 |
| NZ_JAAIOO010000028.1 | *Coprococcus comes* | MSK.11.50 |
| NZ_JAAIOP010000029.1 | *Coprococcus comes* | MSK.11.23 |
| NZ_JAAIOQ010000010.1 | *Coprococcus comes* | MSK.10.14 |
| NZ_JABMHY010000019.1 | *Coprococcus comes* | MSK.17.45 |
| NZ_JABWDC010000075.1 | *Coprococcus comes* | F22 |
| NZ_JADMOE010000022.1 | *Coprococcus comes* | 1001283B150304_161114_E1 |
| NZ_JADMRF010000029.1 | *Coprococcus comes* | D54t1_190329_E4 |
| NZ_JADMUX010000044.1 | *Coprococcus comes* | 1001713B170207_170306_A5 |
| NZ_JADMWX010000021.1 | *Coprococcus comes* | BSD2780120875b_170604_E1 |
| NZ_JADMXP010000003.1 | *Coprococcus comes* | D40t1_170626_H11 |
| NZ_JADMYO010000001.1 | *Coprococcus comes* | J1101004_170508_C2 |
| NZ_JADNAW010000095.1 | *Coprococcus comes* | D6t1_180914_H5 |
| NZ_JADNES010000001.1 | *Coprococcus comes* | D54t1_190329_C12 |
| NZ_JADNFB010000021.1 | *Coprococcus comes* | D41t1_190614_C3 |
| NZ_JADNGX010000021.1 | *Coprococcus comes* | 1001713B170207_170306_E2 |
| NZ_JADNIC010000006.1 | *Coprococcus comes* | 1001271B_151109_E1 |
| NZ_JADNIG010000010.1 | *Coprococcus comes* | D55t1_190419_D6 |
| NZ_JADNIP010000001.1 | *Coprococcus comes* | D43t1_170807_C12 |
| NZ_JADNLV010000134.1 | *Coprococcus comes* | 1001095H_141210_H4 |
| NZ_JADNLX010000010.1 | *Coprococcus comes* | D31t1_170403_G3 |
| NZ_JADPCS010000005.1 | *Coprococcus comes* | 1001283B150225_161107_A5 |
| NZ_JAJCMV010000022.1 | *Coprococcus comes* | DFI.3.85 |
| NZ_JAJCMW010000021.1 | *Coprococcus comes* | DFI.3.84 |
| NZ_JAQLDL010000009.1 | *Coprococcus comes* | D40st1_C9_D40t1_170626 |
| NZ_JAQLDM010000003.1 | *Coprococcus comes* | D40st1_A2_D40t1_170626 |
| NZ_JAQNCD010000025.1 | *Coprococcus comes* | D54st2_C8_D54t1_190329 |
| NZ_JAQNCE010000042.1 | *Coprococcus comes* | 1001713st1_G6_1001713B170207_170306 |
| NZ_JAQNCF010000040.1 | *Coprococcus comes* | 1001713st1_E5_1001713B170131_170501 |
| NZ_JAQNCG010000044.1 | *Coprococcus comes* | 1001713st1_D8_1001713B170214_170313 |
| NZ_JAQNCH010000038.1 | *Coprococcus comes* | 1001713st1_C9_1001713B170207_170306 |
| NZ_JBBNFT010000004.1 | *Coprococcus comes* | CLA-AA-H153 |
| NZ_JBBNNB010000005.1 | *Coprococcus comes* | CLA-JM-H33 |
| NZ_JBCJBC010000013.1 | *Coprococcus comes* | 1033st1_H10_1033SCRN_220408 |
| NZ_JBCPDO010000004.1 | *Coprococcus comes* | RTP21065st1_C5_RTP21065_210128 |
| NZ_JBDGBE010000001.1 | *Coprococcus comes* | RTP21357st1_D5_RTP21357_210408 |
| NZ_JBDMGI010000025.1 | *Coprococcus comes* | RTP31083st1_A10_RTP31083_211112 |
| NZ_JBDMGJ010000021.1 | *Coprococcus comes* | RTP31081st1_E10_RTP31081_211007 |
| NZ_JBDMGK010000027.1 | *Coprococcus comes* | RTP31015st1_F9_RTP31015_201113 |
| NZ_JBDMGL010000005.1 | *Coprococcus comes* | RTP31015st1_E7_RTP31015_201113 |
| NZ_JBDMGM010000003.1 | *Coprococcus comes* | RTP31015st1_E4_RTP31015_201113 |
| NZ_JBDMGN010000005.1 | *Coprococcus comes* | RTP31015st1_D2_RTP31015_201113 |
| NZ_JBDMSI010000014.1 | *Coprococcus comes* | 1001271st1_H1_1001271B_151109 |
| NZ_JBDMSJ010000006.1 | *Coprococcus comes* | 1001271st1_E1_1001271B_151109 |
| NZ_QRHO01000008.1 | *Coprococcus comes* | AM23-3 |
| NZ_QRIM01000002.1 | *Coprococcus comes* | AM22-12LB |
| NZ_QRXJ01000007.1 | *Coprococcus comes* | AF18-12LB |
| NZ_QRXY01000006.1 | *Coprococcus comes* | AF16-31 |
| NZ_QSOV01000013.1 | *Coprococcus comes* | TM07-19 |
| NZ_WQPG01000091.1 | *Coprococcus comes* | MCC412 |
| NZ_WQPH01000099.1 | *Coprococcus comes* | MCC409 |
| NZ_WQPO01000087.1 | *Coprococcus comes* | MCC346 |
| NZ_CP102277.1 | *Coprococcus comes ATCC 27758* | ATCC 27758 |
| NZ_FNWC01000007.1 | *Coprococcus phoceensis* | Marseille-P3062T |
| NZ_JAQCSY010000020.1 | *Coprococcus sp. AF102-57* | AF102-57 |
| NZ_JAQCZB010000011.1 | *Coprococcus sp. AF51-11b1* | AF51-11b1 |
| NZ_JAQDGZ010000012.1 | *Coprococcus sp. AF99-45* | AF99-45 |
| NZ_JAQDKN010000007.1 | *Coprococcus sp. AM100_B19A* | AM100_B19A |
| NZ_JAQDVB010000015.1 | *Coprococcus sp. AM11-30B* | AM11-30B |
| NZ_JAQESG010000029.1 | *Coprococcus sp. AM97-06* | AM97-06 |
| NZ_JAQESL010000003.1 | *Coprococcus sp. AM97-35* | AM97-35 |
| NZ_JANGBB010000007.1 | *Coprococcus sp. DFI.6.81* | DFI.6.81 |
| NZ_JAQEUC010000019.1 | *Coprococcus sp. LG100-32* | LG100-32 |
| NZ_JAQEUJ010000013.1 | *Coprococcus sp. LG101-27* | LG101-27 |
| NZ_JBCPDP010000015.1 | *Coprococcus sp. RTP21204st1_G4_RTP21204_210225* | RTP21204st1_G4_RTP21204_210225 |
| NZ_JAQEKA010000037.1 | *Coprococcus sp. TM115-141* | TM115-141 |
| NZ_JAQEKS010000022.1 | *Coprococcus sp. TM115-187* | TM115-187 |
| NZ_SGXF01000001.1 | *Cuneatibacter caecimuris* | DSM 29486 |
| NZ_JALIFO010000015.1 | *Cuneatibacter sp. NSJ-177* | NSJ-177 |
| NZ_LMBX01000004.1 | *Cytobacillus praedii* | FJAT-25547 |
| NZ_JADNRQ010000001.1 | *Desulfallas sp. Bu1-1* | Bu1-1 |
| NZ_VNHM01000011.1 | *Desulfallas thermosapovorans DSM 6562* | DSM 6562 |
| NZ_FRDN01000003.1 | *Desulfitobacterium chlororespirans DSM 11544* | DSM 11544 |
| NZ_CABKQQ010000052.1 | *Desulfitobacterium hafniense* |  |
| NZ_LK996017.1 | *Desulfitobacterium hafniense* | PCE-S |
| NZ_LOCK01000094.1 | *Desulfitobacterium hafniense* | DH |
| NC_011830.1 | *Desulfitobacterium hafniense DCB-2* | DCB-2 |
| NZ_JH414483.1 | *Desulfitobacterium hafniense DP7* | DP7 |
| NC_007907.1 | *Desulfitobacterium hafniense Y51* | Y51 |
| NZ_FQUW01000009.1 | *Desulfofundulus australicus DSM 11792* | DSM 11792 |
| NC_015573.1 | *Desulfofundulus kuznetsovii DSM 6115* | DSM 6115 |
| NZ_JAUSUX010000003.1 | *Desulfofundulus luciae* | DSM 12396 |
| NZ_RBWE01000001.1 | *Desulfofundulus salinus* | 435 |
| NZ_JAAOEF010000001.1 | *Desulfofundulus sp. TPOSR* | TPOSR |
| NZ_JANWUT010000003.1 | *Desulfofundulus thermocisternus* | AL36 |
| NZ_JMLG01000002.1 | *Desulfofundulus thermocisternus DSM 10259* | DSM 10259 |
| NZ_FQZM01000030.1 | *Desulfofundulus thermosubterraneus DSM 16057* | DSM 16057 |
| NC_021184.1 | *Desulfoscipio gibsoniae DSM 7213* | DSM 7213 |
| NZ_MASS01000001.1 | *Desulfosporosinus sp. BG* | BG |
| NZ_NADJ01000012.1 | *Desulfosporosinus sp. FKB* | FKB |
| NZ_JMGA01000015.1 | *Desulfosporosinus sp. HMP52* | HMP52 |
| NZ_LYVF01000137.1 | *Desulfotomaculum copahuensis* | LMa1 |
| NZ_MIJE01000022.1 | *Desulfuribacillus alkaliarsenatis* | AHT28 |
| NZ_JAPDNO010000005.1 | *Dethiobacter alkaliphilus* | Z1002 |
| NZ_CABHNI010000038.1 | *Dorea formicigenerans* |  |
| NZ_CABJBB010000005.1 | *Dorea formicigenerans* |  |
| NZ_CP102279.1 | *Dorea formicigenerans* | ATCC 27755 |
| NZ_JAAIMA010000003.1 | *Dorea formicigenerans* | MSK.17.61 |
| NZ_JAAIOE010000009.1 | *Dorea formicigenerans* | MSK.23.29 |
| NZ_JAAIOF010000014.1 | *Dorea formicigenerans* | MSK.13.39 |
| NZ_JAAIOG010000024.1 | *Dorea formicigenerans* | MSK.13.15 |
| NZ_JAAITG010000019.1 | *Dorea formicigenerans* | MSK.19.39 |
| NZ_JABAFX010000027.1 | *Dorea formicigenerans* | BSM-383-APC-5F |
| NZ_JAJCNY010000023.1 | *Dorea formicigenerans* | DFI.1.97 |
| NZ_JAJCOA010000023.1 | *Dorea formicigenerans* | DFI.1.90 |
| NZ_JAJCOB010000024.1 | *Dorea formicigenerans* | DFI.1.81 |
| NZ_JAJCOE010000023.1 | *Dorea formicigenerans* | DFI.1.75 |
| NZ_JAJCOF010000023.1 | *Dorea formicigenerans* | DFI.1.72 |
| NZ_JAJCOG010000027.1 | *Dorea formicigenerans* | DFI.1.64 |
| NZ_JAJCOI010000025.1 | *Dorea formicigenerans* | DFI.1.58 |
| NZ_JAJCOO010000025.1 | *Dorea formicigenerans* | DFI.1.38 |
| NZ_JAJCPE010000023.1 | *Dorea formicigenerans* | DFI.1.230 |
| NZ_JAJCPH010000026.1 | *Dorea formicigenerans* | DFI.1.224 |
| NZ_JAJCPK010000024.1 | *Dorea formicigenerans* | DFI.1.220 |
| NZ_JAJCPL010000023.1 | *Dorea formicigenerans* | DFI.1.219 |
| NZ_JAJCPO010000023.1 | *Dorea formicigenerans* | DFI.1.215 |
| NZ_JAJCPS010000026.1 | *Dorea formicigenerans* | DFI.1.210 |
| NZ_JAJCPZ010000024.1 | *Dorea formicigenerans* | DFI.1.180 |
| NZ_JAJCQA010000024.1 | *Dorea formicigenerans* | DFI.1.18 |
| NZ_JAJCQM010000026.1 | *Dorea formicigenerans* | DFI.1.155 |
| NZ_JAJDKV010000026.1 | *Dorea formicigenerans* | DFI.6.93 |
| NZ_JAKNDW010000023.1 | *Dorea formicigenerans* | DFI.1.231 |
| NZ_JAKNDY010000024.1 | *Dorea formicigenerans* | DFI.1.235 |
| NZ_JAKNGK010000027.1 | *Dorea formicigenerans* | DFI.6.65 |
| NZ_JANGEB010000024.1 | *Dorea formicigenerans* | DFI.1.232 |
| NZ_JAQDGW010000014.1 | *Dorea formicigenerans* | AF99-31 |
| NZ_JAQDKF010000007.1 | *Dorea formicigenerans* | AM100_B1_35 |
| NZ_JAQDKG010000002.1 | *Dorea formicigenerans* | AM100_B1_35A |
| NZ_JBBNJA010000011.1 | *Dorea formicigenerans* | CLA-AA-H264 |
| NZ_JBBNPT010000015.1 | *Dorea formicigenerans* | CLA-SR-H027 |
| NZ_JBDMGG010000022.1 | *Dorea formicigenerans* | RTP31015st1_G6_RTP31015_201113 |
| NZ_JBDMGH010000005.1 | *Dorea formicigenerans* | RTP31015st1_B3_RTP31015_201113 |
| NZ_QRHN01000016.1 | *Dorea formicigenerans* | AM23-7AC |
| NZ_QRPD01000005.1 | *Dorea formicigenerans* | AF36-1BH |
| NZ_QRQQ01000010.1 | *Dorea formicigenerans* | AF31-13BH |
| NZ_QRUK01000016.1 | *Dorea formicigenerans* | AF25-11 |
| NZ_QRWH01000006.1 | *Dorea formicigenerans* | AF19-4AC |
| NZ_QRWS01000009.1 | *Dorea formicigenerans* | AF19-13 |
| NZ_QSFS01000015.1 | *Dorea formicigenerans* | AM42-8 |
| NZ_QSGQ01000005.1 | *Dorea formicigenerans* | AM40-15AC |
| NZ_QSHK01000014.1 | *Dorea formicigenerans* | AM37-5 |
| NZ_QSHP01000014.1 | *Dorea formicigenerans* | AM37-11 |
| NZ_QSKA01000013.1 | *Dorea formicigenerans* | AM29-11 |
| NZ_QSOI01000006.1 | *Dorea formicigenerans* | TM09-19AC |
| NZ_QSOK01000006.1 | *Dorea formicigenerans* | TM09-16AC |
| NZ_QSPL01000008.1 | *Dorea formicigenerans* | TM05-5 |
| NZ_QSQM01000014.1 | *Dorea formicigenerans* | TF12-1 |
| NZ_QSQQ01000012.1 | *Dorea formicigenerans* | TF11-11 |
| NZ_QSRA01000017.1 | *Dorea formicigenerans* | TF09-3 |
| NZ_QSVB01000018.1 | *Dorea formicigenerans* | OM03-2 |
| NZ_QSVQ01000022.1 | *Dorea formicigenerans* | OM02-12 |
| NZ_WQPB01000008.1 | *Dorea formicigenerans* | MCC444 |
| NZ_WQPC01000006.1 | *Dorea formicigenerans* | MCC443 |
| NZ_AAXA02000016.1 | *Dorea formicigenerans ATCC 27755* | ATCC 27755 |
| NZ_JAJCNF010000007.1 | *Dorea sp. 210702-DFI.3.125* | DFI.3.125 |
| NZ_JAJCNB010000019.1 | *Dorea sp. 210702-DFI.3.17* | DFI.3.17 |
| NZ_ATVU01000004.1 | *Dorea sp. AGR2135* | AGR2135 |
| NZ_QUCZ01000010.1 | *Dorea sp. AM13-35* | AM13-35 |
| NZ_OLMI01000002.1 | *Dorea sp. Marseille-P4042* | Marseille-P4042 |
| NZ_JBDOHT010000001.1 | *Dorea sp. YH-dor228* | YH-dor228 |
| NZ_CP034413.3 | *Dysosmobacter welbionis* | J115 |
| NZ_JANGBC010000009.1 | *Dysosmobacter welbionis* | DFI.6.79 |
| NZ_JAWQDG010000007.1 | *Dysosmobacter welbionis* | CM711B_48 |
| NZ_AQXL01000120.1 | *Effusibacillus pohliae DSM 22757* | DSM 22757 |
| NZ_LT969517.1 | *Eisenbergiella massiliensis* | AT11 |
| NZ_QVLU01000008.1 | *Eisenbergiella massiliensis* | AF26-4BH |
| NZ_QVLV01000003.1 | *Eisenbergiella massiliensis* | TF05-5AC |
| NZ_QVLV01000009.1 | *Eisenbergiella massiliensis* | TF05-5AC |
| NZ_JAQDDK010000004.1 | *Eisenbergiella porci* | AF73-11pH9TA |
| NZ_VUMI01000002.1 | *Eisenbergiella porci* | WCA-389-WT-23B |
| NZ_VUMI01000091.1 | *Eisenbergiella porci* | WCA-389-WT-23B |
| NZ_QUES01000039.1 | *Eisenbergiella sp. OF01-20* | OF01-20 |
| NZ_JAAITT010000051.1 | *Enterocloster aldenensis* | MSK.1.17 |
| NZ_JAJCID010000012.1 | *Enterocloster aldenensis* | DFI.3.50 |
| NZ_JAKNGE010000008.1 | *Enterocloster aldenensis* | DFI.6.55 |
| NZ_JAQFCM010000022.1 | *Enterocloster aldenensis* | TF09-12AC |
| NZ_JAUDCM010000010.1 | *Enterocloster aldenensis* | ET318 |
| NZ_JAWQDI010000032.1 | *Enterocloster aldenensis* | CM710M_44 |
| NZ_JBBNNR010000015.1 | *Enterocloster aldenensis* | CLA-JM-H53 |
| NZ_JBCLPI010000019.1 | *Enterocloster aldenensis* | 61-73 |
| NZ_QVEX01000010.1 | *Enterocloster aldenensis* | AF19-9LB |
| NZ_WTVF01000013.1 | *Enterocloster aldenensis* | MSK.1.13 |
| NZ_CABMHH010000092.1 | *Enterocloster asparagiformis* |  |
| NZ_QSBM01000002.1 | *Enterocloster asparagiformis* | AF04-15 |
| NZ_JADMNW010000009.1 | *Enterocloster bolteae* | 1001713B170214_170313_G12 |
| NZ_JADMVR010000006.1 | *Enterocloster bolteae* | 1001302B_160321_D9 |
| NZ_JADNDB010000002.1 | *Enterocloster bolteae* | D31t1_170403_B11 |
| NZ_JADNLR010000002.1 | *Enterocloster bolteae* | D40t1_170626_F8 |
| NZ_JADNPF010000006.1 | *Enterocloster bolteae* | 1001287H_170206_C8 |
| NZ_JAJCIN010000034.1 | *Enterocloster bolteae* | DFI.2.21 |
| NZ_JAJCJE010000010.1 | *Enterocloster bolteae* | SL.1.21 |
| NZ_JAJCLG010000005.1 | *Enterocloster bolteae* | DFI.1.195 |
| NZ_JAJCPT010000004.1 | *Enterocloster bolteae* | DFI.1.207 |
| NZ_JAKNDQ010000005.1 | *Enterocloster bolteae* | DFI.1.16 |
| NZ_JAKNDS010000003.1 | *Enterocloster bolteae* | DFI.1.209 |
| NZ_JANFYA010000001.1 | *Enterocloster bolteae* | SL.2.16 |
| NZ_JANKAI010000002.1 | *Enterocloster bolteae* | DSM 29485 |
| NZ_JAQEBA010000003.1 | *Enterocloster bolteae* | AM27-28LB |
| NZ_JAQEEO010000007.1 | *Enterocloster bolteae* | AM60-12MH-1A |
| NZ_JBBNFS010000032.1 | *Enterocloster bolteae* | CLA-AA-H15 |
| NZ_JBCOSV010000019.1 | *Enterocloster bolteae* | RTP31004st1_E3_RTP31004_210507 |
| NZ_QRTI01000007.1 | *Enterocloster bolteae* | AF27-9 |
| NZ_QRUZ01000007.1 | *Enterocloster bolteae* | AF24-13 |
| NZ_QRZM01000005.1 | *Enterocloster bolteae* | AF14-18 |
| NZ_QSQX01000015.1 | *Enterocloster bolteae* | TF09-4AC |
| NZ_QYRW01000026.1 | *Enterocloster bolteae* | AHG0001 |
| NZ_KB851183.1 | *Enterocloster bolteae 90A5* | 90A5 |
| NZ_KB851171.1 | *Enterocloster bolteae 90B7* | 90B7 |
| NZ_KB851156.1 | *Enterocloster bolteae 90B8* | 90B8 |
| NZ_KQ235845.1 | *Enterocloster bolteae WAL-14578* | WAL-14578 |
| NZ_JAQDJP010000015.1 | *Enterocloster citroniae* | AM09-31 |
| NZ_BJLB01000001.1 | *Enterocloster clostridioformis* | NBRC 113352 |
| NZ_CP050964.1 | *Enterocloster clostridioformis* | FDAARGOS_739 |
| NZ_CZAB01000002.1 | *Enterocloster clostridioformis* | 2789STDY5834865 |
| NZ_FOIO01000030.1 | *Enterocloster clostridioformis* | NLAE-zl-C196 |
| NZ_FOJH01000030.1 | *Enterocloster clostridioformis* | NLAE-zl-G208 |
| NZ_FOOJ01000009.1 | *Enterocloster clostridioformis* | ATCC 25537 |
| NZ_JAAIND010000008.1 | *Enterocloster clostridioformis* | MSK.2.80 |
| NZ_JAAISQ010000008.1 | *Enterocloster clostridioformis* | MSK.2.98 |
| NZ_JAAISR010000008.1 | *Enterocloster clostridioformis* | MSK.2.94 |
| NZ_JAAISS010000008.1 | *Enterocloster clostridioformis* | MSK.2.90 |
| NZ_JAAIST010000008.1 | *Enterocloster clostridioformis* | MSK.2.78 |
| NZ_JAAISU010000008.1 | *Enterocloster clostridioformis* | MSK.2.63 |
| NZ_JAAISV010000009.1 | *Enterocloster clostridioformis* | MSK.2.59 |
| NZ_JAAISW010000013.1 | *Enterocloster clostridioformis* | MSK.2.26 |
| NZ_JABMIB010000008.1 | *Enterocloster clostridioformis* | MSK.2.73 |
| NZ_JADMWI010000056.1 | *Enterocloster clostridioformis* | 1001175B_160314_C5 |
| NZ_JADPAG010000005.1 | *Enterocloster clostridioformis* | J1101653_170612_A4 |
| NZ_JADYUU010000005.1 | *Enterocloster clostridioformis* | ET46 |
| NZ_JAIWZC010000001.1 | *Enterocloster clostridioformis* | FDAARGOS_1529 |
| NZ_JAQDHE010000006.1 | *Enterocloster clostridioformis* | AFF13-24A |
| NZ_JAQLCZ010000199.1 | *Enterocloster clostridioformis* | J1101653st1_B2_J1101653_170612 |
| NZ_JAQLDA010000007.1 | *Enterocloster clostridioformis* | J1100826st1_G3_J1100826_190719 |
| NZ_JAQLDB010000154.1 | *Enterocloster clostridioformis* | D35st1_B2_D35t1_190705 |
| NZ_JAQLDC010000121.1 | *Enterocloster clostridioformis* | D35st1_B1_D35t1_190705 |
| NZ_JBBNNP010000006.1 | *Enterocloster clostridioformis* | CLA-JM-H51 |
| NZ_JBCPCJ010000012.1 | *Enterocloster clostridioformis* | RTP31106st1_A10_RTP31106_210429 |
| NZ_JBCPCK010000012.1 | *Enterocloster clostridioformis* | RTP31106st1_A6_RTP31106_210429 |
| NZ_JBCPCL010000014.1 | *Enterocloster clostridioformis* | RTP31106st1_B11_RTP31106_210429 |
| NZ_JBCPCM010000103.1 | *Enterocloster clostridioformis* | RTP31106st1_C9_RTP31106_210429 |
| NZ_JBCPCN010000013.1 | *Enterocloster clostridioformis* | RTP31106st1_G10_RTP31106_210429 |
| NZ_JBDMHU010000003.1 | *Enterocloster clostridioformis* | RTP21489st1_B4_RTP31083_211112 |
| NZ_JBDMHV010000049.1 | *Enterocloster clostridioformis* | RTP21489st1_A4_RTP21489_200313 |
| NZ_JBDOZM010000007.1 | *Enterocloster clostridioformis* | J1101653st1_H5_J1100826_190719 |
| NZ_JBDOZN010000030.1 | *Enterocloster clostridioformis* | J1101653st1_F9_J1101653_170612 |
| NZ_JBDOZO010000010.1 | *Enterocloster clostridioformis* | J1100826st1_D9_J1100826_190719 |
| NZ_SPGN01000056.1 | *Enterocloster clostridioformis* | 1001175st1_C5 |
| NZ_UAVW01000020.1 | *Enterocloster clostridioformis* | NCTC11224 |
| NZ_CABJCG010000012.1 | *Enterocloster lavalensis* |  |
| NZ_FOIM01000022.1 | *Enterocloster lavalensis* | NLAE-zl-G277 |
| NZ_JAJCRP010000006.1 | *Enterocloster lavalensis* | SL.2.12 |
| NZ_JBCOHT010000027.1 | *Enterocloster lavalensis* | RTP21230st1_A7_RTP21230_210325 |
| NZ_JBCOHU010000002.1 | *Enterocloster lavalensis* | RTP21230st1_F2_RTP21230_210325 |
| NZ_JBCOHV010000007.1 | *Enterocloster lavalensis* | RTP21230st1_F6_RTP21230_210325 |
| NZ_JBCOHW010000288.1 | *Enterocloster lavalensis* | RTP21230st1_H1_RTP21230_210325 |
| NZ_PYII01000006.1 | *Enterocloster lavalensis* | KCTC 15153 |
| NZ_JAJCHK010000031.1 | *Enterocloster sp. 210928-DFI.2.20* | DFI.2.20 |
| NZ_JAKNTH010000001.1 | *Enterocloster sp. OA11* | OA11 |
| NZ_CABJBY010000018.1 | *Enterococcus asini* |  |
| NZ_JADMDV010000027.1 | *Enterococcus asini* | U1910977 |
| NZ_JAQCUM010000008.1 | *Enterococcus asini* | AF13-5H |
| NZ_JARQAK010000033.1 | *Enterococcus asini* | P63-2 |
| NZ_JARQAQ010000026.1 | *Enterococcus asini* | P7-2 |
| NZ_JARQAS010000019.1 | *Enterococcus asini* | P1-2 |
| NZ_JARQBJ010000007.1 | *Enterococcus asini* | B226-2 |
| NZ_JBBNKN010000008.1 | *Enterococcus asini* | CLA-AC-H002 |
| NZ_JBEFQE010000011.1 | *Enterococcus asini* | EB2-12 |
| NZ_QRZW01000018.1 | *Enterococcus asini* | AF13-3-H |
| NZ_JAFBFD010000043.1 | *Enterococcus lemanii* | DSM 105069 |
| NZ_PIEU01000022.1 | *Enterococcus plantarum* | TRW2 |
| NZ_NGMO01000003.1 | *Enterococcus sp. 10A9_DIV0425* | 10A9_DIV0425 |
| NZ_RCYI01000001.1 | *Eubacterium sp. am_0171* | am_0171 |
| NZ_WKZL01000006.1 | *Eubacterium sp. BIOML-A1* | BIOML-A1 |
| NZ_WKZK01000001.1 | *Eubacterium sp. BIOML-A2* | BIOML-A2 |
| NZ_JADMRI010000023.1 | *Eubacterium sp. BSD2780061688b_171218_H5* | BSD2780061688b_171218_H5 |
| NZ_JPJE01000021.1 | *Eubacterium sp. ER2* | ER2 |
| NZ_LN865150.1 | *Eubacterium sp. SB2* | SB2 |
| NZ_JAJCRU010000024.1 | *Extibacter muris* | SL.2.04 |
| NZ_JANFYC010000037.1 | *Extibacter muris* | SL.2.08 |
| NZ_JANFYD010000039.1 | *Extibacter muris* | SL.2.07 |
| NZ_JAOQJX010000004.1 | *Faecalicatena acetigenes* | H2_18 |
| NZ_BAAACT010000069.1 | *Faecalicatena contorta* | JCM 6483 |
| NZ_BQNQ01000001.1 | *Faecalicatena contorta* | CE91-St64 |
| NZ_CABKUE010000006.1 | *Faecalicatena contorta* |  |
| NZ_CYZU01000004.1 | *Faecalicatena contorta* | 2789STDY5834876 |
| NZ_JACSNM010000029.1 | *Faecalicatena contorta* | An846 |
| NZ_JACSNO010000003.1 | *Faecalicatena contorta* | An826 |
| NZ_JADNFP010000001.1 | *Faecalicatena contorta* | 1001302B_160321_D10 |
| NZ_JAFBIN010000004.1 | *Faecalicatena contorta* | An936 |
| NZ_JAFBIX010000009.1 | *Faecalicatena contorta* | ET55 |
| NZ_JAFBIY010000001.1 | *Faecalicatena contorta* | ET52 |
| NZ_RDBT01000015.1 | *Faecalicatena contorta* | D5-21 |
| NZ_JACLYY010000005.1 | *Faecalicatena fissicatena* | An773 |
| NZ_LOED01000023.1 | *Fervidicola ferrireducens* | Y170 |
| NZ_CP089291.1 | *Fodinisporobacter ferrooxydans* | MYW30-H2 |
| NZ_JADKNH010000013.1 | *Fusibacter ferrireducens* | Q10-2 |
| NZ_FQZV01000031.1 | *Geosporobacter subterraneus DSM 17957* | DSM 17957 |
| NZ_SMAG01000002.1 | *Hazenella coriacea* | DSM 45707 |
| NC_010337.2 | *Heliomicrobium modesticaldum Ice1* | Ice1; ATCC 51547 |
| NZ_WXEY01000017.1 | *Heliomicrobium undosum* | DSM 13378 |
| NZ_JAOQNU010000016.1 | *Heliophilum fasciatum* | MTM |
| NZ_SLXT01000017.1 | *Heliophilum fasciatum* | DSM 11170 |
| NZ_CALPCM010000008.1 | *Hominisplanchenecus murintestinalis* | DSM 111139 |
| NZ_SRZB01000005.1 | *Hominisplanchenecus murintestinalis* | NM72_1-8 |
| NZ_JBBMFC010000003.1 | *Hominiventricola aquisgranensis* | CLA-AA-H78B |
| NZ_CP102274.1 | *Hungatella hathewayi* | DSM 13479 |
| NZ_JADNLE010000008.1 | *Hungatella hathewayi* | 1001175B_160314_A10 |
| NZ_JAQDZV010000001.1 | *Hungatella hathewayi* | AM21-13LB |
| NZ_JBDMEE010000087.1 | *Hungatella hathewayi* | RTP31083st1_D9_RTP31083_211112 |
| NZ_JBDMEF010000091.1 | *Hungatella hathewayi* | RTP31083st1_A6_RTP31083_211112 |
| NZ_QSGX01000028.1 | *Hungatella hathewayi* | AM39-16AC |
| NZ_QSGX01000033.1 | *Hungatella hathewayi* | AM39-16AC |
| NZ_QVIA01000022.1 | *Hungatella hathewayi* | AF19-21 |
| NZ_JBDOQL010000203.1 | *Hydrogenibacillus schlegelii* | DSM 2000-1 |
| NZ_JBFMHT010000283.1 | *Hydrogenibacillus schlegelii* | DSM 2000-2 |
| NZ_JBFMHU010000304.1 | *Hydrogenibacillus schlegelii* | DSM 2000-3 |
| NZ_JXBB01000045.1 | *Hydrogenibacillus schlegelii* | MA 48 |
| NZ_JYFD01000158.1 | *Hydrogenibacillus schlegelii* | DSM 2000 |
| NZ_CP080953.1 | *Hydrogenibacillus sp. N12* | N12 |
| NZ_CP024955.1 | *Kyrpidia spormannii* | EA-1 |
| NC_014098.1 | *Kyrpidia tusciae DSM 2912* | DSM 2912 |
| NZ_CABMKL010000001.1 | *Lachnoclostridium edouardi* |  |
| NZ_OESQ01000001.1 | *Lachnoclostridium edouardi* | Marseille-P3397 |
| NZ_LT635479.1 | *Lachnoclostridium phocaeense* | Marseille-P3177T |
| NZ_NFLQ01000009.1 | *Lachnoclostridium sp. An118* | An118 |
| NZ_NFKJ01000020.1 | *Lachnoclostridium sp. An181* | An181 |
| NZ_NFJZ01000013.1 | *Lachnoclostridium sp. An196* | An196 |
| NZ_JAHLQB010000110.1 | *Lachnoclostridium sp. MSJ-17* | MSJ-17 |
| NZ_AZUI01000001.1 | *Lacrimispora indolis DSM 755* | DSM 755 |
| NZ_ATXD01000007.1 | *Lacrimispora indolis SR3* | SR3 |
| NZ_CP157940.1 | *Lacrimispora sp. BS-2* | BS-2 |
| NZ_KZ119600.1 | *Litchfieldia alkalitelluris* | 10368 |
| NZ_UPPP01000072.1 | *Lucifera butyrica* |  |
| NZ_UWOE01000001.1 | *Luxibacter massiliensis* | Marseille-P5551 |
| NZ_CP152047.1 | *Lysinibacillus sp. FSL K6-0057* | FSL K6-0057 |
| NZ_CP150264.1 | *Lysinibacillus sp. FSL K6-3209* |  |
| NZ_NFLD01000009.1 | *Massilimicrobiota sp. An142* | An142 |
| NZ_JACJKO010000012.1 | *Massilimicrobiota timonensis* | An731 |
| NZ_UYXN01000023.1 | *Massilimicrobiota timonensis* |  |
| NZ_BHGK01000001.1 | *Mediterraneibacter butyricigenes* | KCTC 15684 |
| NZ_JADPGD010000002.1 | *Mediterraneibacter faecis* | 1001713B170131_170501_C12 |
| NZ_JADPGZ010000004.1 | *Mediterraneibacter faecis* | D59t2_181005_E7 |
| NZ_JADPLP010000007.1 | *Mediterraneibacter faecis* | 1001285H_161024_C3 |
| NZ_JAJBMF010000006.1 | *Mediterraneibacter faecis* | MSK.13.52 |
| NZ_JAJBMJ010000005.1 | *Mediterraneibacter faecis* | MSK.7.18 |
| NZ_JAJBNZ010000007.1 | *Mediterraneibacter faecis* | MSK.13.58 |
| NZ_JAJBOA010000009.1 | *Mediterraneibacter faecis* | MSK.13.54 |
| NZ_JAJBOB010000014.1 | *Mediterraneibacter faecis* | MSK.13.49 |
| NZ_JAJBOC010000007.1 | *Mediterraneibacter faecis* | MSK.13.44 |
| NZ_JAJBOE010000006.1 | *Mediterraneibacter faecis* | MSK.13.46 |
| NZ_JAJBOF010000016.1 | *Mediterraneibacter faecis* | MSK.13.42 |
| NZ_JAJBQK010000009.1 | *Mediterraneibacter faecis* | MSK.13.18 |
| NZ_JAJBQL010000009.1 | *Mediterraneibacter faecis* | MSK.10.13 |
| NZ_JAJBQM010000010.1 | *Mediterraneibacter faecis* | MSK.13.9 |
| NZ_JAJCHS010000022.1 | *Mediterraneibacter faecis* | DFI.4.88 |
| NZ_JAJCHW010000019.1 | *Mediterraneibacter faecis* | DFI.4.18 |
| NZ_JAJCHY010000019.1 | *Mediterraneibacter faecis* | DFI.4.116 |
| NZ_JAJCIG010000010.1 | *Mediterraneibacter faecis* | DFI.3.118 |
| NZ_JAJCIY010000001.1 | *Mediterraneibacter faecis* | DFI.4.12 |
| NZ_JAKNFG010000018.1 | *Mediterraneibacter faecis* | DFI.4.70 |
| NZ_JAKNIP010000015.1 | *Mediterraneibacter faecis* | MSK.20.22 |
| NZ_JAKNIQ010000015.1 | *Mediterraneibacter faecis* | MSK.20.47 |
| NZ_JAKNIR010000004.1 | *Mediterraneibacter faecis* | MSK.20.83 |
| NZ_JAKNIS010000016.1 | *Mediterraneibacter faecis* | MSK.20.86 |
| NZ_JAKNIT010000015.1 | *Mediterraneibacter faecis* | MSK.20.90 |
| NZ_JAKNIU010000015.1 | *Mediterraneibacter faecis* | MSK.20.91 |
| NZ_JAQDKE010000010.1 | *Mediterraneibacter faecis* | AM100_B1_31A |
| NZ_JAQDKU010000002.1 | *Mediterraneibacter faecis* | AM100_B33A |
| NZ_JAQDRE010000015.1 | *Mediterraneibacter faecis* | AM109-29 |
| NZ_JAQDUE010000004.1 | *Mediterraneibacter faecis* | AM110-68 |
| NZ_JAQEBE010000008.1 | *Mediterraneibacter faecis* | AM27-9LB |
| NZ_JAQEDB010000001.1 | *Mediterraneibacter faecis* | AM44-6 |
| NZ_JBBNGH010000002.1 | *Mediterraneibacter faecis* | CLA-AA-H178 |
| NZ_JBBNJZ010000003.1 | *Mediterraneibacter faecis* | CLA-AA-H308 |
| NZ_JBDOJD010000005.1 | *Mediterraneibacter faecis* | RTP21487st1_H4_RTP21489_200313 |
| NZ_WNAF01000006.1 | *Mediterraneibacter faecis* | BIOML-A1 |
| NZ_WQNO01000044.1 | *Mediterraneibacter faecis* | MCC719 |
| NZ_JACJKE010000006.1 | *Mediterraneibacter glycyrrhizinilyticus* | An785 |
| NZ_JAUDCC010000001.1 | *Mediterraneibacter glycyrrhizinilyticus* | ET488 |
| NZ_JAUDCQ010000011.1 | *Mediterraneibacter glycyrrhizinilyticus* | ET229 |
| NZ_JAUEIO010000004.1 | *Mediterraneibacter glycyrrhizinilyticus* | 39_SSukc10 |
| NZ_CP070036.1 | *Mediterraneibacter gnavus* | FDAARGOS_1342 |
| NZ_CP096671.1 | *Mediterraneibacter gnavus* | CC55_001C |
| NZ_CP111084.1 | *Mediterraneibacter gnavus* | JCM6515 |
| NZ_CP111086.1 | *Mediterraneibacter gnavus* | JCM6515 |
| NZ_CYZG01000022.1 | *Mediterraneibacter gnavus* | 2789STDY5608852 |
| NZ_JAAILN010000031.1 | *Mediterraneibacter gnavus* | MSK.5.19 |
| NZ_JAAILZ010000021.1 | *Mediterraneibacter gnavus* | MSK.17.63 |
| NZ_JAAIMR010000037.1 | *Mediterraneibacter gnavus* | MSK.15.58 |
| NZ_JAAIMS010000025.1 | *Mediterraneibacter gnavus* | MSK.5.17 |
| NZ_JAAIMT010000020.1 | *Mediterraneibacter gnavus* | MSK.23.91 |
| NZ_JAAING010000028.1 | *Mediterraneibacter gnavus* | MSK.19.38 |
| NZ_JAAIQW010000024.1 | *Mediterraneibacter gnavus* | MSK.7.31 |
| NZ_JAAIQX010000024.1 | *Mediterraneibacter gnavus* | MSK.7.28 |
| NZ_JAAIQY010000008.1 | *Mediterraneibacter gnavus* | MSK.23.93 |
| NZ_JAAIQZ010000026.1 | *Mediterraneibacter gnavus* | MSK.23.92 |
| NZ_JAAIRA010000026.1 | *Mediterraneibacter gnavus* | MSK.23.82 |
| NZ_JAAIRB010000026.1 | *Mediterraneibacter gnavus* | MSK.23.81 |
| NZ_JAAIRC010000008.1 | *Mediterraneibacter gnavus* | MSK.23.71 |
| NZ_JAAIRD010000008.1 | *Mediterraneibacter gnavus* | MSK.23.63 |
| NZ_JAAIRE010000060.1 | *Mediterraneibacter gnavus* | MSK.23.62 |
| NZ_JAAIRF010000008.1 | *Mediterraneibacter gnavus* | MSK.23.61 |
| NZ_JAAIRG010000026.1 | *Mediterraneibacter gnavus* | MSK.23.60 |
| NZ_JAAIRH010000028.1 | *Mediterraneibacter gnavus* | MSK.23.56 |
| NZ_JAAIRI010000008.1 | *Mediterraneibacter gnavus* | MSK.23.55 |
| NZ_JAAIRJ010000008.1 | *Mediterraneibacter gnavus* | MSK.23.46 |
| NZ_JAAIRK010000026.1 | *Mediterraneibacter gnavus* | MSK.23.43 |
| NZ_JAAIRL010000027.1 | *Mediterraneibacter gnavus* | MSK.23.41 |
| NZ_JAAIRM010000023.1 | *Mediterraneibacter gnavus* | MSK.22.53 |
| NZ_JAAIRN010000027.1 | *Mediterraneibacter gnavus* | MSK.22.24 |
| NZ_JAAIRO010000029.1 | *Mediterraneibacter gnavus* | MSK.19.33 |
| NZ_JAAIRP010000021.1 | *Mediterraneibacter gnavus* | MSK.17.82 |
| NZ_JAAIRQ010000037.1 | *Mediterraneibacter gnavus* | MSK.15.9 |
| NZ_JAAIRR010000019.1 | *Mediterraneibacter gnavus* | MSK.15.77 |
| NZ_JAAIRS010000035.1 | *Mediterraneibacter gnavus* | MSK.15.59 |
| NZ_JAAIRT010000036.1 | *Mediterraneibacter gnavus* | MSK.15.56 |
| NZ_JAAIRU010000028.1 | *Mediterraneibacter gnavus* | MSK.15.54 |
| NZ_JAAIRV010000038.1 | *Mediterraneibacter gnavus* | MSK.15.32 |
| NZ_JAAIRW010000036.1 | *Mediterraneibacter gnavus* | MSK.15.18 |
| NZ_JAAIRX010000036.1 | *Mediterraneibacter gnavus* | MSK.15.10 |
| NZ_JAAIRY010000030.1 | *Mediterraneibacter gnavus* | MSK.11.9 |
| NZ_JADMPO010000022.1 | *Mediterraneibacter gnavus* | D52t1_170925_A9 |
| NZ_JADMPV010000016.1 | *Mediterraneibacter gnavus* | BSD2780120874_150323_G6 |
| NZ_JADMTD010000010.1 | *Mediterraneibacter gnavus* | D31t1_170403_H6 |
| NZ_JADMTQ010000043.1 | *Mediterraneibacter gnavus* | D33t1_170424_E3 |
| NZ_JADNGF010000017.1 | *Mediterraneibacter gnavus* | D40t1_170626_D9 |
| NZ_JADNIZ010000005.1 | *Mediterraneibacter gnavus* | 1001285H_161024_A10 |
| NZ_JADNOC010000017.1 | *Mediterraneibacter gnavus* | 1001302B_160321_G6 |
| NZ_JADPDH010000022.1 | *Mediterraneibacter gnavus* | BSD2780061687_150420_H1 |
| NZ_JADYUQ010000026.1 | *Mediterraneibacter gnavus* | ET50 |
| NZ_JAJBLZ010000015.1 | *Mediterraneibacter gnavus* | MSK.7.5 |
| NZ_JAJBNC010000025.1 | *Mediterraneibacter gnavus* | MSK.23.4 |
| NZ_JAJBOJ010000025.1 | *Mediterraneibacter gnavus* | MSK.23.20 |
| NZ_JAJBOM010000006.1 | *Mediterraneibacter gnavus* | MSK.23.18 |
| NZ_JAJBON010000025.1 | *Mediterraneibacter gnavus* | MSK.23.17 |
| NZ_JAJBPH010000006.1 | *Mediterraneibacter gnavus* | MSK.23.10 |
| NZ_JAJBPJ010000004.1 | *Mediterraneibacter gnavus* | MSK.22.96 |
| NZ_JAJBPM010000006.1 | *Mediterraneibacter gnavus* | MSK.22.91 |
| NZ_JAKNIW010000026.1 | *Mediterraneibacter gnavus* | MSK.23.27 |
| NZ_JALQCM010000001.1 | *Mediterraneibacter gnavus* | S107-86 |
| NZ_JALQCN010000006.1 | *Mediterraneibacter gnavus* | S107-61 |
| NZ_JANFXP010000020.1 | *Mediterraneibacter gnavus* | SL.2.31 |
| NZ_JAPRAU010000026.1 | *Mediterraneibacter gnavus* | PS/160 |
| NZ_JAPRAV010000008.1 | *Mediterraneibacter gnavus* | CCUG 57137 |
| NZ_JAPRAW010000022.1 | *Mediterraneibacter gnavus* | CCUG 57208 |
| NZ_JAPRAX010000021.1 | *Mediterraneibacter gnavus* | CCUG 57161 |
| NZ_JAPRAY010000018.1 | *Mediterraneibacter gnavus* | CCUG 49994 |
| NZ_JAPRAZ010000004.1 | *Mediterraneibacter gnavus* | CCUG 43437 |
| NZ_JAPRBA010000012.1 | *Mediterraneibacter gnavus* | CCUG 43222 |
| NZ_JAPRBB010000023.1 | *Mediterraneibacter gnavus* | CCUG 54531 |
| NZ_JAPRBC010000006.1 | *Mediterraneibacter gnavus* | CCUG 51289 |
| NZ_JAPRBD010000020.1 | *Mediterraneibacter gnavus* | CCUG 52279 |
| NZ_JAPZEC010000008.1 | *Mediterraneibacter gnavus* | RSHDN_121 |
| NZ_JAPZED010000008.1 | *Mediterraneibacter gnavus* | RSHDN_123 |
| NZ_JAPZEG010000007.1 | *Mediterraneibacter gnavus* | RSHDN_120 |
| NZ_JAPZEH010000009.1 | *Mediterraneibacter gnavus* | RSHDN_122 |
| NZ_JAQDNS010000032.1 | *Mediterraneibacter gnavus* | AM10-1A |
| NZ_JAQESN010000027.1 | *Mediterraneibacter gnavus* | AM97-54 |
| NZ_JAQMLA010000037.1 | *Mediterraneibacter gnavus* | RTP21484st1_H11_RTP21484_190118 |
| NZ_JAQMLB010000036.1 | *Mediterraneibacter gnavus* | RTP21484st1_E7_RTP21484_190118 |
| NZ_JAQMLC010000037.1 | *Mediterraneibacter gnavus* | RTP21484st1_C12_RTP21484_190118 |
| NZ_JAQMLD010000009.1 | *Mediterraneibacter gnavus* | J1101312st1_A9_J1101312_190322 |
| NZ_JAQMLE010000009.1 | *Mediterraneibacter gnavus* | J1101312st1_A7_J1101312_190322 |
| NZ_JAQMLF010000008.1 | *Mediterraneibacter gnavus* | J1101312st1_A12_J1101312_190322 |
| NZ_JAQMLG010000026.1 | *Mediterraneibacter gnavus* | D33st1_G9_D33t1_170424 |
| NZ_JAQMLH010000022.1 | *Mediterraneibacter gnavus* | BSD3448080968st1_G7_BSD3448080968_160620 |
| NZ_JAQMLI010000017.1 | *Mediterraneibacter gnavus* | BSD2780120874st1_F6_2217SCRN_200827 |
| NZ_JAQMLJ010000019.1 | *Mediterraneibacter gnavus* | BSD2780120874st1_E4_2217SCRN_200827 |
| NZ_JAQMLK010000021.1 | *Mediterraneibacter gnavus* | BSD2780061687st1_E4_BSD2780061687_150420 |
| NZ_JAQMLL010000022.1 | *Mediterraneibacter gnavus* | 2217st1_H4_2217SCRN_200827 |
| NZ_JAQMLM010000021.1 | *Mediterraneibacter gnavus* | 2217st1_G3_2217SCRN_200827 |
| NZ_JAQMLN010000018.1 | *Mediterraneibacter gnavus* | 1001285st1_H5_1001285H_161024 |
| NZ_JAQMLO010000009.1 | *Mediterraneibacter gnavus* | 1001217st1_G9_1001217B_191108 |
| NZ_JAQMLP010000002.1 | *Mediterraneibacter gnavus* | 1001217st1_D6_1001217B_191108 |
| NZ_JAQMLQ010000009.1 | *Mediterraneibacter gnavus* | 1001217st1_B7_1001217B_191108 |
| NZ_JAQMLR010000009.1 | *Mediterraneibacter gnavus* | 1001217st1_A9_1001217B_191108 |
| NZ_JAQOWV010000007.1 | *Mediterraneibacter gnavus* | RSHDN_120 |
| NZ_JAQOWW010000009.1 | *Mediterraneibacter gnavus* | RSHDN_122 |
| NZ_JBBNLE010000037.1 | *Mediterraneibacter gnavus* | CLA-AP-H37 |
| NZ_JBBNNO010000021.1 | *Mediterraneibacter gnavus* | CLA-JM-H50 |
| NZ_JBCIUQ010000018.1 | *Mediterraneibacter gnavus* | RTP31141st1_H1_RTP31141_220114 |
| NZ_JBCIUR010000018.1 | *Mediterraneibacter gnavus* | RTP31141st1_H7_RTP31141_220114 |
| NZ_JBCIUS010000037.1 | *Mediterraneibacter gnavus* | RTP31141st2_D6_RTP31141_220114 |
| NZ_JBCIUT010000030.1 | *Mediterraneibacter gnavus* | RTP31141st2_E3_RTP31141_220114 |
| NZ_JBCOVY010000205.1 | *Mediterraneibacter gnavus* | RTP21484st1_A3_RTP31023_210422 |
| NZ_JBCOVZ010000037.1 | *Mediterraneibacter gnavus* | RTP21484st1_C11_RTP31023_210422 |
| NZ_JBCOWA010000048.1 | *Mediterraneibacter gnavus* | RTP21484st1_E11_RTP31023_210422 |
| NZ_JBCPGB010000014.1 | *Mediterraneibacter gnavus* | D40st1_A9_RTP21276_210326 |
| NZ_JBCPGC010000003.1 | *Mediterraneibacter gnavus* | RTP21281st1_G9_RTP21281_210402 |
| NZ_JBDGDE010000008.1 | *Mediterraneibacter gnavus* | RTP21359st1_C1_RTP21359_211015 |
| NZ_JBDGDF010000012.1 | *Mediterraneibacter gnavus* | RTP21359st1_E7_RTP21359_211015 |
| NZ_JBDGDG010000003.1 | *Mediterraneibacter gnavus* | RTP21359st1_F11_RTP21359_211015 |
| NZ_JBDGDH010000024.1 | *Mediterraneibacter gnavus* | RTP21360st1_D12_RTP21360_211022 |
| NZ_JBDGDI010000036.1 | *Mediterraneibacter gnavus* | RTP21360st1_F11_RTP21360_211022 |
| NZ_JBDGDJ010000018.1 | *Mediterraneibacter gnavus* | RTP21360st2_E9_RTP21360_211022 |
| NZ_JBDMCU010000022.1 | *Mediterraneibacter gnavus* | RTP21400st2_H3_RTP21400_201112 |
| NZ_JBDMCV010000019.1 | *Mediterraneibacter gnavus* | RTP21400st1_D2_RTP21400_201112 |
| NZ_JBDMCW010000023.1 | *Mediterraneibacter gnavus* | RTP21360st1_H11_RTP21489_200313 |
| NZ_JBDMCX010000024.1 | *Mediterraneibacter gnavus* | RTP21360st1_G1_RTP21489_200313 |
| NZ_JBDMCY010000024.1 | *Mediterraneibacter gnavus* | RTP21360st1_F8_RTP21489_200313 |
| NZ_JBDMCZ010000025.1 | *Mediterraneibacter gnavus* | RTP21360st1_D1_RTP21489_200313 |
| NZ_JBDMDA010000025.1 | *Mediterraneibacter gnavus* | RTP21360st1_C8_RTP21489_200313 |
| NZ_JBDPGS010000013.1 | *Mediterraneibacter gnavus* | BSD3448080968st1_E11_BSD3448080968_160620 |
| NZ_JBDPGT010000023.1 | *Mediterraneibacter gnavus* | 2217st1_D3_2217SCRN_200827 |
| NZ_JBDPGU010000029.1 | *Mediterraneibacter gnavus* | 2217st1_A7_2217SCRN_200827 |
| NZ_JBDPIC010000010.1 | *Mediterraneibacter gnavus* | D31st1_F1_D31t1_170403 |
| NZ_NIHM01000007.1 | *Mediterraneibacter gnavus* | RJX1118 |
| NZ_NIHN01000009.1 | *Mediterraneibacter gnavus* | RJX1119 |
| NZ_NIHO01000054.1 | *Mediterraneibacter gnavus* | RJX1120 |
| NZ_NIHP01000019.1 | *Mediterraneibacter gnavus* | RJX1121 |
| NZ_NIHQ01000015.1 | *Mediterraneibacter gnavus* | RJX1122 |
| NZ_NIHR01000015.1 | *Mediterraneibacter gnavus* | RJX1123 |
| NZ_NIHS01000023.1 | *Mediterraneibacter gnavus* | RJX1124 |
| NZ_NIHT01000023.1 | *Mediterraneibacter gnavus* | RJX1125 |
| NZ_NIHU01000005.1 | *Mediterraneibacter gnavus* | RJX1126 |
| NZ_NIHV01000006.1 | *Mediterraneibacter gnavus* | RJX1127 |
| NZ_NIHW01000027.1 | *Mediterraneibacter gnavus* | RJX1128 |
| NZ_QRIA01000003.1 | *Mediterraneibacter gnavus* | AM22-7AC |
| NZ_QRIP01000029.1 | *Mediterraneibacter gnavus* | AM21-41 |
| NZ_QRIS01000030.1 | *Mediterraneibacter gnavus* | AM21-18 |
| NZ_QRJQ01000021.1 | *Mediterraneibacter gnavus* | AM17-51 |
| NZ_QRLD01000014.1 | *Mediterraneibacter gnavus* | AM13-39 |
| NZ_QRLN01000005.1 | *Mediterraneibacter gnavus* | AM12-54 |
| NZ_QRPU01000020.1 | *Mediterraneibacter gnavus* | AF34-4BH |
| NZ_QRQE01000022.1 | *Mediterraneibacter gnavus* | AF33-12 |
| NZ_QRTJ01000018.1 | *Mediterraneibacter gnavus* | AF27-4BH |
| NZ_QRUR01000002.1 | *Mediterraneibacter gnavus* | AF24-23LB |
| NZ_QRWQ01000001.1 | *Mediterraneibacter gnavus* | AF19-16AC |
| NZ_QSAA01000028.1 | *Mediterraneibacter gnavus* | AF13-14A |
| NZ_QSHH01000022.1 | *Mediterraneibacter gnavus* | AM38-12 |
| NZ_QSIR01000027.1 | *Mediterraneibacter gnavus* | AM32-6 |
| NZ_QSKP01000002.1 | *Mediterraneibacter gnavus* | AM27-32 |
| NZ_QSPZ01000030.1 | *Mediterraneibacter gnavus* | TM04-16 |
| NZ_JAGQ01000004.1 | *Mediterraneibacter gnavus AGR2154* | AGR2154 |
| NZ_AAYG02000033.1 | *Mediterraneibacter gnavus ATCC 29149* | ATCC 29149 |
| NZ_CP027002.1 | *Mediterraneibacter gnavus ATCC 29149* | ATCC 29149 |
| NZ_CP043051.1 | *Mediterraneibacter gnavus ATCC 29149* | JCM6515 |
| NZ_CP102291.1 | *Mediterraneibacter gnavus ATCC 29149* | ATCC 29149 |
| NZ_PUEL01000004.1 | *Mediterraneibacter gnavus ATCC 29149* | ATCC 29149 |
| NZ_KI669418.1 | *Mediterraneibacter gnavus CC55_001C* | CC55_001C |
| NZ_JACOPF010000003.1 | *Mediterraneibacter hominis* | NSJ-55 |
| NZ_JAQEHW010000014.1 | *Mediterraneibacter massiliensis* | AM78-01pH3A |
| NZ_LN913000.1 | *Mediterraneibacter massiliensis* | Marseille-P2086 |
| NZ_JAJCNG010000010.1 | *Mediterraneibacter sp. 210702-DFI.3.120* | DFI.3.120 |
| NZ_JAJCME010000005.1 | *Mediterraneibacter sp. 210702-DFI.5.30* | DFI.5.30 |
| NZ_SIHS01000023.1 | *Mediterraneibacter sp. gm002* | gm002 |
| NZ_SIHS01000038.1 | *Mediterraneibacter sp. gm002* | gm002 |
| NZ_JBBKBE010000023.1 | *Mediterraneibacter sp. ICN-202921* | ICN-202921 |
| NZ_CP136418.1 | *Moorella humiferrea* | LNE |
| NZ_CP136419.1 | *Moorella humiferrea* | 64-FGQ |
| NZ_CP136421.1 | *Moorella humiferrea* | OCP |
| NZ_PVXM01000006.1 | *Moorella humiferrea* | DSM 23265 |
| NZ_LTBC01000017.1 | *Moorella mulderi DSM 14980* | DSM 14980 |
| NZ_BJKO01000001.1 | *Moorella sp. E306M* | E306M |
| NZ_BJKN01000001.1 | *Moorella sp. E308F* | E308F |
| NZ_PVXL01000006.1 | *Moorella stamsii* | DSM 26217 |
| NZ_JANRHG010000007.1 | *Moorella sulfitireducens* | SLA38 |
| NZ_BSDM01000004.1 | *Moorella thermoacetica* | G-20 |
| NZ_CP012369.1 | *Moorella thermoacetica* | DSM 521 |
| NZ_CP012370.1 | *Moorella thermoacetica* | DSM 2955 |
| NZ_CP017019.1 | *Moorella thermoacetica* | DSM 103132 |
| NZ_CP017237.1 | *Moorella thermoacetica* | DSM 103284 |
| NZ_CP031054.1 | *Moorella thermoacetica* | 39073-HH |
| NZ_CP136416.1 | *Moorella thermoacetica* | BGP |
| NZ_CP136417.1 | *Moorella thermoacetica* | MBA |
| NZ_CP136425.1 | *Moorella thermoacetica* | KAM |
| NZ_CP136551.1 | *Moorella thermoacetica* | COM |
| NZ_MDDB01000004.1 | *Moorella thermoacetica* | DSM 6867 |
| NZ_MDDD01000007.1 | *Moorella thermoacetica* | DSM 12993 |
| NZ_MDDE01000008.1 | *Moorella thermoacetica* | DSM 7417 |
| NZ_MIHH01000027.1 | *Moorella thermoacetica* | DSM 11768 |
| NZ_MIHH01000042.1 | *Moorella thermoacetica* | DSM 11768 |
| NZ_MIHH01000079.1 | *Moorella thermoacetica* | DSM 11768 |
| NZ_MIIF01000009.1 | *Moorella thermoacetica* | DSM 12797 |
| NZ_VCDV01000005.1 | *Moorella thermoacetica* | ATCC 31490 |
| NZ_VCDW01000004.1 | *Moorella thermoacetica* | ATCC 35608 |
| NZ_VCDX01000010.1 | *Moorella thermoacetica* | ATCC 33924 |
| NZ_VCDY01000004.1 | *Moorella thermoacetica* | ATCC 49707 |
| NZ_DF238840.1 | *Moorella thermoacetica Y72* | Y72 |
| NZ_SLZZ01000015.1 | *Muricomes intestini* | DSM 29489 |
| NZ_JAKNTQ010000005.1 | *Muricomes sp. OA1* | OA1 |
| NZ_CP054394.1 | *Natranaerofaba carboxydovora* | ANCO1 |
| NZ_CALBWS010000031.1 | *Neobacillus rhizosphaerae* | CIP 111895 |
| NZ_CP126093.1 | *Neobacillus sp. DY30* | DY30 |
| NZ_JAOQKG010000004.1 | *Oscillibacter acetigenes* | H4_59 |
| NZ_AP018532.1 | *Oscillibacter sp. PEA192* | PEA192 |
| NZ_WSQB01000014.1 | *Paenibacillus alvei* | MP1 |
| NZ_CP021780.1 | *Paenibacillus donghaensis* | KCTC 13049 |
| NZ_BTYA01000020.1 | *Paenibacillus elgii* | YSY-1.2 |
| NZ_BTYA01000046.1 | *Paenibacillus elgii* | YSY-1.2 |
| NZ_JALRMO010000001.1 | *Paenibacillus hamazuiensis* | YIM B00624 |
| NZ_JALPRK010000007.1 | *Paenibacillus mellifer* | MBLB2552 |
| NZ_FNDY01000003.1 | *Paenibacillus naphthalenovorans* | PR-N1 |
| NZ_MKQK01000079.1 | *Paenibacillus odorifer* | FSL F4-0152 |
| NZ_MKQP01000014.1 | *Paenibacillus odorifer* | FSL H7-0604 |
| NZ_MPTV01000008.1 | *Paenibacillus odorifer* | FSL H8-0237 |
| NZ_JAHZIJ010000015.1 | *Paenibacillus oenotherae* | DT7-4 |
| NZ_VIJZ01000019.1 | *Paenibacillus ottowii* | MS2379 |
| NZ_VCIX01000086.1 | *Paenibacillus paridis* | py1325 |
| NZ_BIMM01000032.1 | *Paenibacillus pasadenensis* | NBRC 101214 |
| NZ_NFEZ01000004.1 | *Paenibacillus pasadenensis* | R16 |
| NZ_AULW01000036.1 | *Paenibacillus pasadenensis DSM 19293* | DSM 19293 |
| NZ_CP092831.1 | *Paenibacillus peoriae* | ZBSF16 |
| NZ_CP132974.1 | *Paenibacillus peoriae* | JJ21 |
| NZ_JAVDUG010000001.1 | *Paenibacillus peoriae* | BE143 |
| NZ_MRTC01000008.1 | *Paenibacillus peoriae* | FSL A5-0030 |
| NZ_MRTM01000010.1 | *Paenibacillus peoriae* | FSL H8-0551 |
| NZ_BAVZ01000016.1 | *Paenibacillus pini JCM 16418* | JCM 16418 |
| NZ_BAZT01000016.1 | *Paenibacillus pini JCM 16418* | JCM 16418 |
| NZ_WHNY01000009.1 | *Paenibacillus plantarum* | LMG 31461 |
| NZ_ALJV01000043.1 | *Paenibacillus polymyxa* | WLY78 |
| NZ_CP011420.1 | *Paenibacillus polymyxa* | ATCC 15970 |
| NZ_CP017968.3 | *Paenibacillus polymyxa* | YC0573 |
| NZ_CP109848.1 | *Paenibacillus polymyxa* | K16 |
| NZ_JAEHFQ010000006.1 | *Paenibacillus polymyxa* | LMG 27872 |
| NZ_JAFJXZ010000017.1 | *Paenibacillus polymyxa* | Hob6 |
| NZ_JAWPHF010000012.1 | *Paenibacillus polymyxa* | CAPE238 |
| NZ_LYMX01000138.1 | *Paenibacillus polymyxa* | CFSAN034341 |
| NZ_LYMY01000168.1 | *Paenibacillus polymyxa* | CFSAN034342 |
| NZ_LYND01000033.1 | *Paenibacillus polymyxa* | CFSAN034343 |
| NZ_OXKC02000007.1 | *Paenibacillus polymyxa* | DSM 292 |
| NC_023037.2 | *Paenibacillus polymyxa CR1* | CR1 |
| NZ_SADY01000015.1 | *Paenibacillus popilliae* | SDF0028 |
| NZ_BMFU01000011.1 | *Paenibacillus silvae* | CGMCC 1.12770 |
| NZ_JAFICR010000019.1 | *Paenibacillus sp. 1182* | 1182 |
| NZ_JAWDJI010000213.1 | *Paenibacillus sp. 3LSP* | 3LSP |
| NZ_JANAVE010000001.1 | *Paenibacillus sp. A3M_27_13* | A3M_27_13 |
| NZ_CP159992.1 | *Paenibacillus sp. AN1007* | AN1007 |
| NZ_JAIVEM010000167.1 | *Paenibacillus sp. BJ-4* | BJ-4 |
| NZ_FOYG01000003.1 | *Paenibacillus sp. cl130* | CL130 |
| NZ_JAAMNU010000014.1 | *Paenibacillus sp. EKM205P* | EKM205P |
| NZ_JAAMNQ010000017.1 | *Paenibacillus sp. EKM206P* | EKM206P |
| NZ_BCPS01000058.1 | *Paenibacillus sp. EPM92* | EPM92 |
| NZ_CP151963.1 | *Paenibacillus sp. FSL H7-0442* | FSL H7-0442 |
| NZ_JBBOPF010000001.1 | *Paenibacillus sp. FSL H7-0703* | FSL H7-0703 |
| NZ_CP151948.1 | *Paenibacillus sp. FSL H8-0168* | FSL H8-0168 |
| NZ_CP152049.1 | *Paenibacillus sp. FSL H8-0548* | FSL H8-0548 |
| NZ_MRTK01000016.1 | *Paenibacillus sp. FSL H8-0548* | FSL H8-0548 |
| NZ_ASPV01000126.1 | *Paenibacillus sp. FSL H8-237* | FSL H8-237 |
| NZ_JBBOUW010000001.1 | *Paenibacillus sp. FSL L8-0641* | FSL L8-0641 |
| NZ_JBBOYN010000001.1 | *Paenibacillus sp. FSL L8-0644* | FSL L8-0644 |
| NZ_JBBOYM010000001.1 | *Paenibacillus sp. FSL L8-0663* | FSL L8-0663 |
| NZ_JBBOXV010000001.1 | *Paenibacillus sp. FSL M7-0134* | FSL M7-0134 |
| NZ_JBBOSD010000001.1 | *Paenibacillus sp. FSL M7-0802* | FSL M7-0802 |
| NZ_JBBOXA010000001.1 | *Paenibacillus sp. FSL M8-0228* | FSL M8-0228 |
| NZ_CP150243.1 | *Paenibacillus sp. FSL P2-0322* |  |
| NZ_JBBOYJ010000001.1 | *Paenibacillus sp. FSL P4-0288* | FSL P4-0288 |
| NZ_JBBOBN010000001.1 | *Paenibacillus sp. FSL R10-2736* | FSL R10-2736 |
| NZ_MRTQ01000006.1 | *Paenibacillus sp. FSL R5-0490* | FSL R5-0490 |
| NZ_CP150225.1 | *Paenibacillus sp. FSL R5-0928* |  |
| NZ_JBBOCC010000002.1 | *Paenibacillus sp. FSL R7-0179* | FSL R7-0179 |
| NZ_CP152001.1 | *Paenibacillus sp. FSL R7-0652* | FSL R7-0652 |
| NZ_ASPX01000025.1 | *Paenibacillus sp. FSL R7-277* | FSL R7-277 |
| NZ_CP150181.1 | *Paenibacillus sp. FSL W8-1187* |  |
| NZ_BOSH01000005.1 | *Paenibacillus sp. J2TS4* | J2TS4 |
| NZ_SGWF01000011.1 | *Paenibacillus sp. JMULE4* | JMULE4 |
| NZ_JAMAWB010000026.1 | *Paenibacillus sp. MER TA 81-3* | MER TA 81-3 |
| NZ_JAHLPR010000060.1 | *Paenibacillus sp. MSJ-34* | MSJ-34 |
| NZ_JANAVJ010000057.1 | *Paenibacillus sp. MZ04-78.2* | MZ04-78.2 |
| NZ_CP093336.1 | *Paenibacillus sp. N3/727* | N3/727 |
| NZ_WUDQ01000624.1 | *Paenibacillus sp. OT2-17* | OT2-17 |
| NZ_JAMBDM010000008.1 | *Paenibacillus sp. p3-SID867* | p3-SID867 |
| NZ_VIDW01000034.1 | *Paenibacillus sp. tmac-D7* | tmac-D7 |
| NZ_JMLR01000009.1 | *Paenibacillus sp. UNCCL52* | UNCCL52 |
| NZ_QMFA01000056.1 | *Paenibacillus sp. YN15* | YN15 |
| NZ_JAKSXN010000027.1 | *Paenibacillus timonensis* | DSM 16943 |
| NZ_JAQDEO010000012.1 | *Paenibacillus timonensis* | AF90-26SAA |
| NZ_JARTHJ010000028.1 | *Paenibacillus validus* | NRS-626 |
| NZ_JARTHK010000013.1 | *Paenibacillus validus* | NRS-625 |
| NZ_BIMH01000028.1 | *Paenibacillus validus NBRC 15382* | NBRC 15382 |
| NZ_CATNVD010000016.1 | *Parablautia sp. Marseille-Q6255* | Marseille-Q6255 |
| NZ_QFFZ01000005.1 | *Pelotomaculum propionicicum* | MGP |
| NZ_QFGB01000079.1 | *Pelotomaculum sp. FP* | FP |
| NZ_JBBPIW010000007.1 | *Priestia aryabhattai* | FSL H7-0740 |
| NZ_CP120965.1 | *Proteiniclasticum sp. QWL-01* | QWL-01 |
| NZ_FOQF01000032.1 | *Pseudobutyrivibrio sp. OR37* | OR37 |
| NZ_WQOJ01000005.1 | *Pseudoflavonifractor sp. MCC625* | MCC625 |
| NZ_CABKRK010000004.1 | *Robertmurraya massiliosenegalensis* |  |
| NZ_CVRR01000082.1 | *Roseburia faecis* | M72 |
| NZ_CYXV01000012.1 | *Roseburia faecis* | 2789STDY5608863 |
| NZ_JADMYF010000005.1 | *Roseburia faecis* | D54t1_190329_D3 |
| NZ_JADNAB010000013.1 | *Roseburia faecis* | 1001713B170207_170306_F11 |
| NZ_JADNLZ010000034.1 | *Roseburia faecis* | BSD2780061689_150309_B4 |
| NZ_JADNPM010000008.1 | *Roseburia faecis* | 1001271B_151109_E4 |
| NZ_JADPAX010000001.1 | *Roseburia faecis* | 1001175B_160314_E3 |
| NZ_JADPDS010000012.1 | *Roseburia faecis* | D53t1_180928_E1 |
| NZ_JAJBML010000007.1 | *Roseburia faecis* | MSK.6.3 |
| NZ_JAJCJO010000006.1 | *Roseburia faecis* | DFI.7.37A |
| NZ_JBBNFX010000004.1 | *Roseburia faecis* | CLA-AA-H164 |
| NZ_JBCIZW010000003.1 | *Roseburia faecis* | 1033st1_F10_1033SCRN_220408 |
| NZ_JBCOAS010000032.1 | *Roseburia faecis* | RTP31139st1_E6_RTP31139_211217 |
| NZ_SPHF01000001.1 | *Roseburia hominis* | 1001175st1_E3 |
| NZ_QRID01000009.1 | *Roseburia intestinalis* | AM22-21LB |
| NZ_QSFP01000006.1 | *Roseburia intestinalis* | AM43-11 |
| NZ_QSHO01000013.1 | *Roseburia intestinalis* | AM37-1AC |
| NC_021012.1 | *Roseburia intestinalis XB6B4* | XB6B4 |
| NZ_CABJFX010000010.1 | *Roseburia inulinivorans* |  |
| NZ_CVRS01000014.1 | *Roseburia inulinivorans* | L1-83 |
| NZ_CYXX01000005.1 | *Roseburia inulinivorans* | 2789STDY5608887 |
| NZ_CYYR01000007.1 | *Roseburia inulinivorans* | 2789STDY5608835 |
| NZ_JBBNFR010000003.1 | *Roseburia inulinivorans* | CLA-AA-H149 |
| NZ_JBBNGM010000007.1 | *Roseburia inulinivorans* | CLA-AA-H186 |
| NZ_QRHP01000012.1 | *Roseburia inulinivorans* | AM23-23AC |
| NZ_QRTF01000014.1 | *Roseburia inulinivorans* | AF28-15 |
| NZ_QRUN01000029.1 | *Roseburia inulinivorans* | AF24-4 |
| NZ_QRVS01000033.1 | *Roseburia inulinivorans* | AF21-31 |
| NZ_QSFX01000010.1 | *Roseburia inulinivorans* | AM42-1AC |
| NZ_QSIQ01000045.1 | *Roseburia inulinivorans* | AM32-8LB |
| NZ_QSKW01000033.1 | *Roseburia inulinivorans* | AM27-11 |
| NZ_WQNS01000023.1 | *Roseburia inulinivorans* | MCC698 |
| NZ_ACFY01000021.1 | *Roseburia inulinivorans DSM 16841* | DSM 16841 |
| NZ_JAJFOJ010000001.1 | *Roseburia inulinivorans DSM 16841* | FDAARGOS_1587 |
| NZ_CP092473.1 | *Roseburia rectibacter* | NSJ-69 |
| NZ_QRHS01000002.1 | *Roseburia sp. AM23-20* | AM23-20 |
| NZ_JAHLQD010000008.1 | *Roseburia sp. MSJ-14* | MSJ-14 |
| NZ_QSCK01000001.1 | *Roseburia sp. OF03-24* | OF03-24 |
| NZ_AORV01000026.1 | *Ruminiclostridium cellobioparum* | CT1112 |
| NZ_FOAT01000020.1 | *Ruminococcus albus* | KH2T6 |
| NZ_JADMNX010000001.1 | *Ruminococcus bicirculans* | D59t2_181005_B8 |
| NZ_JAQMLS010000001.1 | *Ruminococcus bicirculans* | D59st1_B8_D59t2_181005 |
| NZ_JAQMLT010000002.1 | *Ruminococcus bicirculans* | D59st1_B5_D59t2_181005 |
| NZ_JACOPE010000001.1 | *Ruminococcus hominis* | NSJ-13 |
| NZ_QTVE01000007.1 | *Ruminococcus sp. AF37-20* | AF37-20 |
| NZ_RBZR01000027.1 | *Ruminococcus sp. B05* | B05 |
| NZ_RBZR01000033.1 | *Ruminococcus sp. B05* | B05 |
| NZ_JAJEQX010000001.1 | *Ruminococcus turbiniformis* | CLA-AA-H200 |
| NZ_LT891959.1 | *Selenomonas felix* | Marseille-P3560 |
| NZ_LT985755.1 | *Selenomonas massiliensis* | Marseille-P4036 |
| NZ_ALKG01000062.1 | *Selenomonas sp. FOBRC6* | FOBRC6 |
| NZ_CP016201.1 | *Selenomonas sp. oral taxon 126* | W7667 |
| NZ_KB290930.1 | *Selenomonas sp. oral taxon 138 str. F0429* | F0429 |
| NZ_GL397087.1 | *Selenomonas sp. oral taxon 149 str. 67H29BP* | 67H29BP |
| NZ_CP012071.1 | *Selenomonas sp. oral taxon 478* | F0592 |
| NZ_CP060204.1 | *Selenomonas timonae* | Marseille-Q3039 |
| NZ_VUNM01000007.1 | *Sharpea porci* | CA-Schmier-601-WT-3 |
| NZ_JAGSND010000003.1 | *Sinanaerobacter chloroacetimidivorans* | BAD-6 |
| NZ_JFZC01000084.1 | *Sporolactobacillus terrae DSM 11697* | DSM 11697 |
| NZ_JAOQKK010000013.1 | *Suonthocola fibrivorans* | Sanger_33 |
| NZ_VTEV01000005.1 | *Sutcliffiella horikoshii* | CH28_1T |
| NZ_JAGGLG010000022.1 | *Symbiobacterium terraclitae* | DSM 27138 |
| NZ_FRAE01000056.1 | *Tepidibacter formicigenes DSM 15518* | DSM 15518 |
| NZ_WNHB01000001.1 | *Terrilactibacillus tamarindi* | BCM23-1 |
| NC_018870.1 | *Thermacetogenium phaeum DSM 12270* | DSM 12270 |
| NZ_KI912609.1 | *Thermacetogenium phaeum DSM 12270* | DSM 12270 |
| NZ_CP045798.1 | *Thermanaerosceptrum fracticalcis* | DRI-13 |
| NC_014152.1 | *Thermincola potens JR* | JR |
| NZ_JACEOL010000010.1 | *Thermoactinomyces mirandus* | AMNI-1 |
| NZ_CAJRAY010000080.1 | *Thermobacillus xylanilyticus* | XE |
| NZ_FQZP01000073.1 | *Thermoclostridium caenicola* | DSM 19027 |
| NZ_LOHZ01000023.1 | *Thermovenabulum gondwanense* | R270 |
| NZ_JMIR01000060.1 | *Tumebacillus flagellatus* | GST4 |
| NZ_OBMQ01000016.1 | *Ureibacillus xyleni* | JC22 |
| NZ_JBBMFJ010000041.1 | *Ventrimonas faecis* | CLA-AP-H27 |
| NZ_JAFBEA010000001.1 | *Virgibacillus halotolerans* | DSM 25060 |
| NZ_CP085524.1 | *Zhaonella formicivorans* | K32 |
| NZ_JACRYT010000010.1 | *Zhenpiania hominis* | BX12 |
